# Integrating local and landscape management promotes biodiversity, multifunctionality and yields in tropical agroforests

**DOI:** 10.1038/s43247-026-04141-3

**Published:** 2026-10-30

**Authors:** Pablo Aycart-Lazo, Blanca Ivañez-Ballesteros, Abed Alvino-Rosas, Daniel Ochterbeck, Melvin Opolka, Oscar Cabezas Huayllas, Stefan Dullinger, Evert Thomas, Teja Tscharntke, Ingolf Steffan-Dewenter, Carolina Ocampo-Ariza, Bea Maas

**Affiliations:** 1 Department of Botany and Biodiversity Research, University of Vienna, Vienna, Austria; 2 Department of Animal Ecology and Tropical Biology, Biocenter, University of Würzburg, Würzburg, Germany; 3 Universidad Nacional Agraria de la Selva (UNAS), Huanuco, Peru; 4 Agroecology and Functional Agrobiodiversity, Department of Crop Sciences, University of Göttingen, Göttingen, Germany; 5 Department of Conservation Biology, University of Göttingen, Göttingen, Germany; 6 Animal Population Ecology, Bayreuth Center for Ecology and Environmental Research (BayCEER), University of Bayreuth, Bayreuth, Germany; 7 Bioversity International, Lima office, Lima, Peru; 8 Ecological Networks, Technische Universität Darmstadt, Darmstadt, Germany; 9 Institute of Zoology, Department of Ecosystem Management, Climate and Biodiversity, BOKU University, Vienna, Austria

**Keywords:** Agroecology, Community ecology, Ecosystem services, Tropical ecology

## Abstract

Reconciling food production and biodiversity conservation requires socio-ecologically viable agroecosystems. To meet this goal, tropical agroforestry must balance yield-biodiversity trade-offs through local and landscape-level management. By integrating assessments of multi-taxa diversity, biocontrol, pollination, crop pests, soil fertility, microclimate, and yield in Amazonian cacao agroforests, we identified ecological-economic synergies. Yield gains were not associated with species loss. Instead, agroforests with high spatial and vertical shade-tree heterogeneity embedded in landscapes combining 30–35% forest cover and 40–45% of other arboreal habitats (e.g., orchards/agroforests) achieved the highest biodiversity and yields. In these landscapes, flying-vertebrate-mediated arthropod suppression doubled cacao yields. Increasing agroforest shade-tree cover from ≈15% to ≈80% yielded a four-fold rise in cacao fruit-set, tripled soil potassium and enhanced thermal buffering. But light limitation, rather than pest pressure, reduced harvestable yields above 30% shade-tree cover. Our findings demonstrate that combined agroforest-level and landscape-scale management can align biodiversity conservation and economic returns in tropical landscapes.

## Abstract (web summary)

Amazonian cacao agroforests can jointly sustain biodiversity and yields when shade-tree structure and surrounding tree cover are optimized, with vertebrate-mediated pest control doubling yields, based on integrated assessments of biodiversity, ecosystem services, and production.

## Introduction

Over the past decades, the rapid expansion and intensification of agriculture have led to major species declines in tropical regions^1,2^. Agroforestry—the integration of trees into cropping systems (i.e., shade trees)—offers promising opportunities to reconcile food production and biodiversity conservation in tropical agricultural landscapes, while reducing deforestation and enhancing soil health, carbon sequestration, climate adaptation, and food sovereignty^3–7^. Recognizing these benefits, megadiverse countries such as Peru are increasingly promoting agroforestry practices through national agricultural development plans^8^. However, the socio-ecological benefits of agroforestry systems depend on their plot- and landscape-level management, with more intensive agricultural practices typically resulting in yield-biodiversity trade-offs^4,9,10^. Identifying agroforestry management practices and landscape designs optimizing biodiversity conservation and economic returns is thus essential for informing emerging policies and ensuring the long-term sustainability of tropical agricultural landscapes^11^.

Agroforest productivity depends on the ecosystem services and disservices provided by multiple animal groups. Flying arthropods and vertebrates increase cacao and coffee yields in agroforests through crop pollination and phytophagous arthropod suppression, respectively^12–15^. Conversely, fruit predation, arthropod pests, and plant pathogens limit harvestable yields in agroforests^14,16,17^. Although pests and diseases can cause losses of 20–40% of annual global cacao and coffee harvests^17,18^, the economic returns from pollination and biocontrol services in agroforests usually outweigh such losses^14,16^. Both ecosystem services and disservices are influenced by agroforest shade-tree structure, as well as the broader landscape context^16,19–22^. Shade reduction may increase short-term yields, but weakens pest control and pollination services, soil fertility, and agroforest climate-buffering capacity, leading to yield declines over time^23,24^. Similarly, landscape simplification drives decreases in the richness and abundance of pollinators and pest controllers and concomitant productivity declines^25^.

The economic viability of agroforestry systems is also strongly influenced by climate variability, as demonstrated by the weather-related production shortfalls in West Africa and Brazil, which resulted in a nearly 200% increase in global cacao prices between 2022 and 2024^26^. As climate change increasingly affects agroforestry products worldwide, such market fluctuations are likely to become more frequent and severe in the coming decades^27–30^. Optimizing ecosystem service-disservice trade-offs and the climate-buffering capacity of agroforests is thus essential for their long-term stability and preventing further forest conversion^23^. However, we lack comprehensive studies analyzing how agroforest-level and landscape-scale management jointly affect all these factors shaping agroforest productivity and biodiversity conservation in tropical agricultural areas.

Here, we used an integrative, multi-scale approach to test how shade-tree structure and landscape complexity jointly influence biodiversity, ecosystem services, pest and disease incidence, microclimate, soil fertility, and yield in cacao agroforests. By integrating all these variables, we aimed to identify agroforestry management strategies and landscape designs that are both biodiversity-friendly and profitable. We focused on organic agroforests growing cacao (*Theobroma cacao* L.), a major tropical tree crop that sustains the livelihoods of over five million smallholder farmers worldwide^31^. Our study was conducted along the Andean foothills of the Peruvian Amazon, one of the world’s most biodiverse regions, yet increasingly threatened by agriculture-driven deforestation^32^. As the center of origin for cacao, the Western Amazon supports the production of fine-flavor native varieties, providing a high-value socio-ecological and genetic alternative to the intensive bulk-cacao monocultures prevalent in major cacao-producing regions such as Southeast Asia^33^.

Previous studies have found yield-biodiversity trade-offs at increasing canopy closure in cacao agroforests^4,10,34^. However, the effects of other aspects of agroforest canopy structure, such as shade-tree vertical heterogeneity and spatial distribution within the agroforest, and landscape complexity on agroforest multifunctionality and yield relationships remain to be understood. By combining multi-taxa diversity assessments, bird and bat exclusion experiments, evaluations of pollination success, pest and disease incidence, assessments of soil fertility and microclimatic conditions, and cacao yield, we try to disentangle the combined ecological and economic impact of agroforest- and landscape-scale management (Table 1). Drawing on these highly comprehensive assessments, we demonstrate that enhancing the vertical and spatial heterogeneity of shade trees, combined with maintaining forest cover across landscapes, yields tangible win-win outcomes for biodiversity and farmer livelihoods. Our integrated analysis can inform tailored sustainable development strategies for tropical production landscapes^11^, explicitly addressing evidence-based conservation strategies^35^ and higher socio-ecological standards^6^.

**Table 1:** Environmental predictors, response variables, and methods used to assess the influence of shade-tree and landscape management on biodiversity, multifunctionality, and yield in cacao agroforests

Variable		Method/source	Research question
**Environmental predictors**	Agroforest **shade-tree cover**	Drone mapping of agroforests	
	Agroforest **shade-tree** **cover heterogeneity**		
	Agroforest **shade-tree height**		
	Agroforest **shade-tree** **height heterogeneity**		
	**Landscape tree cover**	Published raster layer^82^	
	**Landscape forest cover**	Satellite images	
**Response variables**	**Bird** species richness	Bird point counts	How do shade-tree and landscape management influence **biodiversity** in agroforests?
	**Bat** species richness	Bat recordings	
	**Ant** species richness	Sugar and tuna baits	
	**Butterfly** species richness	Van Someren-Rydon traps	
	**Spider** species richness	Canopy fogging of cacao trees and DNA metabarcoding	
	**Hemiptera** species richness		
	**Beetle** species richness		
	**Multidiversity**	Average standardized richness	
	∆Yield (proxy of **pest control**)	Exclusion experiments (control - exclusion yields)	How do shade-tree and landscape management influence **ecosystem services** in agroforests?
	Fruit-set (proxy of **pollination**)	Monitoring of control trees	
	***Carmenta*** **spp**. incidence	Monitoring of control trees	How do shade-tree and landscape management influence **pests and diseases** in agroforests?
	***Moniliophthora roreri*** incidence		
	Soil **pH, K, P, total N, and organic C**	Soil composition analyses	How does shade-tree management influence agroforest **soil fertility**?
	**Soil moisture**	Soil and understory data loggers	How does shade-tree management influence agroforest **microclimate** across seasons?
	Average and maximum **soil temperatures**		
	Average and maximum **air temperatures**		
	Number of **cacao flowers**	Monitoring of control trees	How do shade-tree and landscape management influence c**acao flower production** in agroforests?
	Kg of dry **cacao beans per tree**	Monitoring of exclusion and control trees	How do shade-tree and landscape management influence **cacao yield** in agroforests?

## Results and discussion

### Win-win shade-tree management for biodiversity and farmers

The conservation value of agroforests is closely linked to their economic performance, as low productivity can drive further forest conversion and biodiversity loss^23^. To identify win-win scenarios for biodiversity and farmer livelihoods, we surveyed birds, bats, ants, butterflies, spiders, Hemiptera species, and beetles in 28 organic cacao agroforests in the northern Peruvian Amazon (see “Methods” section). By participating in multiple ecosystem services and disservices, such as biological pest control and crop damage, the selected taxa serve as valuable ecological indicators of agroforest multifunctionality^36–38^. Additionally, we monitored the annual yield of 128 cacao trees across 16 of the sampled agroforests. Half of the trees were enclosed in bamboo-and-fishing net cages that excluded birds and bats but allowed arthropod access (exclusion trees, four per agroforest), while the other half were untreated (control trees, four per agroforest—following ref. ^39^). We used mixed-effects models to test the single and combined effects of agroforest shade-tree structure and landscape complexity on taxon-specific richness and agroforest multidiversity (i.e., the averaged standardized richness of the seven taxa). Shade-tree structure was represented by four different variables: shade-tree cover, cover heterogeneity (i.e., shade-tree cover standard deviation [sd]), mean height, and height heterogeneity (i.e., shade-tree height sd). Landscape complexity in the surroundings of the agroforest was estimated based on the forest and tree cover in 500-m buffers^40^, the latter including both forests and additional arboreal habitats. Therefore, ‘shade-tree’ variables refer to local, agroforest-level predictors (shade-tree cover, cover heterogeneity, height, and height heterogeneity), landscape forest cover refers to the percentage of the 500-m buffers occupied by forested habitat, and landscape tree cover refers to the percentage of the buffers occupied by trees, including forested habitats, tree crops, and other woody vegetation such as scattered trees. In addition to analyzing how these four shade-tree variables and two landscape predictors affected agroforest biodiversity, we also assessed their influence on cacao yield in control and exclusion trees, biocontrol and pollination services, and pest and disease incidence in the agroforests.

**Fig. 1:**
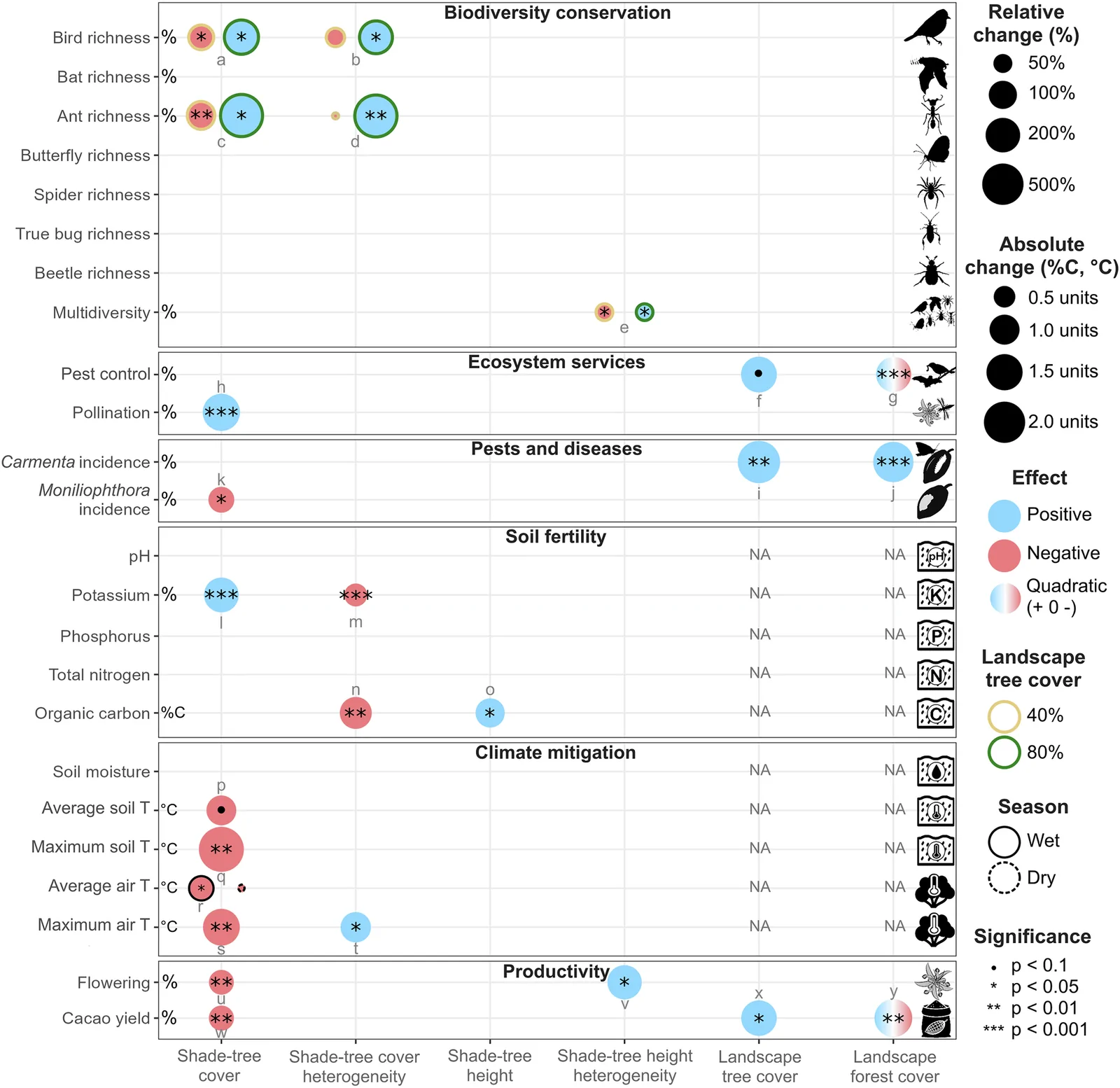
Shade-tree and landscape drivers of agroforest multifunctionality. Circles represent the effects of local shade-tree or landscape predictors named at the bottom of the plot on the response variables named on the left side. Only significant effects or interactions are shown. Circles’ color represents the direction of the effect (positive: blue; negative: red), while their size represents the predicted relative (%, log-scale) or absolute change (in units of %C or °C) in the response variable along the shade-tree or landscape gradient. For quadratic effects, the diameter of the bubble represents the difference between the lowest and highest predicted values of the response variable. For significant interactions between shade-tree predictors and landscape tree cover, evaluated in the biodiversity models only, we represent shade-tree effects at 40% (mean [60%] − one sd [20%]; yellow outline) and 80% (mean + one sd, green outline) landscape tree cover. For significant interaction between temperature (*T*) variables and season, we represent shade-tree and landscape effects in the wet (solid black outline) and dry (dashed black outline) seasons. Landscape effects on agroforest soil fertility and microclimate were not assessed (NA) to avoid correlations between landscape predictors and agroforest elevation. Letters indicate the exact *p*-values of the interactions (int.; generated using two-sided Wald *χ*^2^ tests) or the main linear or quadratic (qua.) effects (generated using two-sided Wald *z*-tests), adjusted for multiple testing using the Bonferroni correction when necessary (see “Methods” section). *P*-values: *a*(int.) = 0.03; *b*(int.) = 0.031; *c*(int.) = 0.002; *d*(int.) = 0.013; *e*(int.) = 0.025; *f* = 0.067; *g*(qua.) = 0.001; *h* < 0.001; *i* = 0.001; *j* < 0.001; *k* = 0.025; *l* < 0.001; *m* = 0.001; *n* = 0.001; *o* = 0.017; *p* = 0.059; *q* = 0.005; *r* = 0.013; *s* = 0.005; *t* = 0.046; *u* = 0.003; *v* = 0.023; *w* = 0.003; *x* = 0.021; *y*(qua.) = 0.007.

**Fig. 2:**
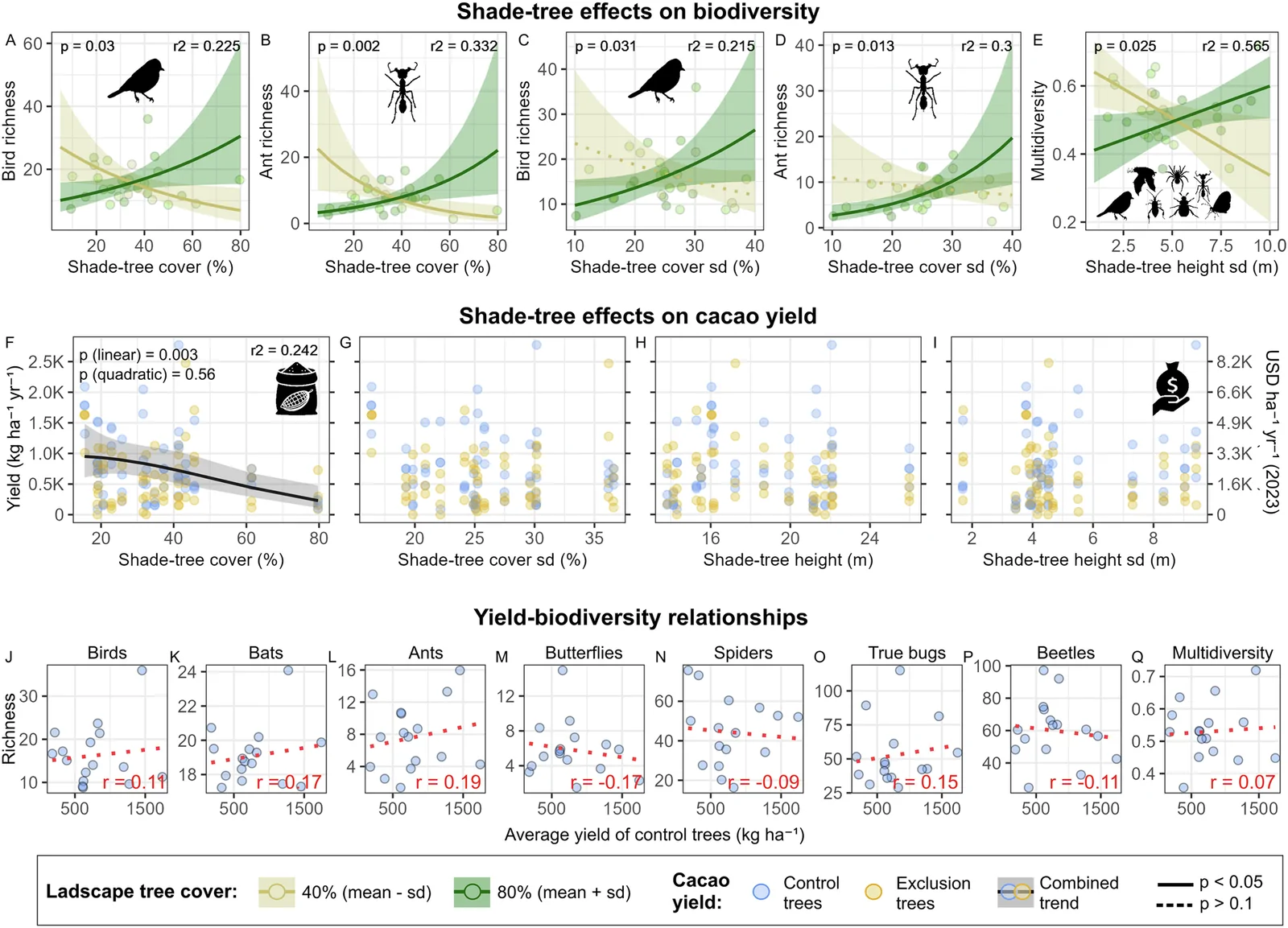
Promoting shade-tree spatial and vertical heterogeneity decouples enhanced biodiversity from yield loss. We only found significant effects of shade-tree structure on bird and ant richness and multidiversity. These effects varied depending on the tree cover in the 500 m surrounding the agroforests (Supplementary Table 1). Therefore, we report biodiversity trends at 40% (mean [60%] – sd [20%]; yellow lines and CIs) and 80% (mean + sd; green lines and CIs) landscape tree cover and the *p*-value of the interaction (generated using Wald *χ*^2^ tests and adjusted for multiple testing using the Bonferroni correction). Raw values and predicted trends with 95% CIs of bird and ant richness along the gradients in shade-tree cover (**A**, **B**) and shade-tree cover heterogeneity (sd; **C**, **D**). **E** Raw values and trends of multidiversity along the shade-tree height heterogeneity (sd) gradient. Biodiversity-environment relationships were measured in 28 agroforests and analyzed using GLMMs. Non-significant biodiversity trends can be found in Supplementary Fig. 2. The results of the biodiversity models can be found in Supplementary Table 2. **F** Raw values and predicted trends with 95% CIs of cacao yield (annual production of dry cacao beans) in 128 trees distributed in 16 agroforests following a shade-tree structure (64 control trees, four per agroforest, blue dots; 64 exclusion trees, four per agroforest, yellow dots). *P*-values in (**F**) were generated using two-sided Wald *z*-tests and adjusted with the Bonferroni correction. Raw values of cacao yield (*n* = 128 trees) along the shade-tree cover heterogeneity (**G**), shade-tree height (**H**), and shade-tree height heterogeneity (**I**), not included in the best-fitting models (Supplementary Table 3). Relationships between the species richness of birds (**J**), bats (**K**), ants (**L**), butterflies (**M**), spiders (**N**), Hemiptera (**O**), beetles (**P**), and multidiversity (**Q**), and the average yield of control trees in agroforests (*n* = 16 agroforests). We represent linear relationships between biodiversity variables and yield (red dotted lines) and their Pearson correlation coefficient (*r*). Tree-level yield values were multiplied by 1100 to scale them to hectares, assuming 3 × 3 m spacing between cacao trees.

Effects of agroforest shade-tree structure on bird and ant richness depended on surrounding landscape complexity, with landscape tree cover having a greater influence on shade-tree effects than landscape forest cover alone (Supplementary Table 1). Conversely, the richness of the other taxa showed no detectable responses to local management or landscape complexity (Fig. 1), consistent with earlier reports for butterflies, beetles, and spiders^41–43^. In simplified landscapes with relatively low tree cover (≈40%; mean tree cover [60%] − one sd [20%]), bird and ant richness declined by 72% and 90%, respectively, as shade-tree cover increased from 10% to 80% (Fig. 2A, B). In contrast, in complex, tree-rich landscapes (≈80% tree cover; mean + one sd), bird richness increased by 180%, and ant richness by 495% along the same gradient. These divergent responses along the shade-tree cover gradient likely display the different community compositions in agroforests located in landscapes with low or high tree cover. Open area generalists, which we expect to be more dominant in landscapes with low tree cover, are likely favored by low-shade management, while forest-associated species whose presence relies on a high tree cover in the landscape require dense shade-tree cover^41,44^. Although these findings indicate that higher shade-tree cover may boost bird and ant richness in agroforests embedded in complex landscapes, increasing shade-tree cover from 15% to 80% reduced cacao yields by 76% (Fig. 2F). However, despite this localized yield-biodiversity trade-off, all biodiversity metrics were weakly correlated with agroforest yield (|Pearson’s *r* | <0.2; Fig. 2J–Q).

On the other hand, our results suggest that enhancing shade-tree spatial and vertical heterogeneity can boost agroforest biodiversity without yield reductions. Increasing shade-tree cover heterogeneity resulted in a 172% increase in bird richness and a 632% increase in ant richness in complex landscapes with 80% tree cover, but contrary to shade-tree cover, it did not affect species richness in landscapes with only 40% tree cover (Fig. 2C, D) nor cacao yields (Fig. 2G–I). Moreover, agroforest multidiversity rose by 46% with increasing shade-tree height heterogeneity from ≈1 to 10 m in complex landscapes with high tree cover, while a similar decrease was observed in the simpler ones (Fig. 2E). Conversely, landscape forest cover did not influence shade-tree effects on multidiversity (Supplementary Table 1). This further indicates that the total landscape tree cover is a stronger moderator of agroforest management effects than forest cover alone, likely because most agroforest species are not forest specialists and may also benefit from alternative arboreal habitats that enhance landscape connectivity^45^.

Vertically continuous shade-tree canopies may preserve microclimatic stability within the understory, facilitating arthropod persistence and fostering multidiversity in simplified landscapes exposed to elevated temperatures and wind^46,47^. In contrast, agroforests combining clusters of shade trees of different heights and open areas likely provide greater niche diversity than those with similar shade-tree cover but composed of uniformly distributed trees of similar heights^48^. This increased environmental heterogeneity seems to favor biodiversity in agroforests located in more complex landscapes, suggesting that high shade-tree cover is not essential to preserve species richness in agroforests if their canopies maintain high vertical and spatial heterogeneity. Doubling shade-tree cover from 15% to 30%—the minimum required to maximize the spatial heterogeneity of shade trees within the agroforests (Supplementary Fig. 1)—is expected to reduce cacao yields by only 10% (Fig. 2F). This yield loss, equivalent to 312 USD ha^−^^1^ yr^−1^ according to 2023 prices ($3.28 USD kg^−^^1^)^26^, may be offset by supplementary income from shade-tree products such as timber and fruits^49^. Together, these findings highlight the combination of spatially and vertically heterogeneous shade-tree canopies in agroforests with extensive landscape-level tree cover as a promising win-win strategy to reconcile biodiversity conservation with farmer livelihoods in tropical agricultural regions.

While tropical agroforests can sustain faunal species richness comparable to adjacent forests, their conservation value is also determined by their capacity to preserve forest-associated species. Previous studies have shown that agroforests typically harbor simplified species assemblages characterized by a decrease in forest specialists and a dominance of generalist species, with implications for shade-tree and landscape management^41,50^. For instance, lower shade-tree cover was related to a decrease in the species richness of forest-associated ants and butterflies in Indonesian and Peruvian agroforests, respectively^41,43^. Additionally, increasing distance to the forest and local management intensity are usually associated with lower richness and abundance of forest birds in cacao agroforests^51,52^. Regarding bats, the sole use of ultrasonic recorders to estimate their species richness prevented us from analyzing shade-tree and landscape management effects on phyllostomid bats (family Phyllostomidae)^53^, many of which are understory and forest-dependent species that do not thrive in simplified landscapes^54^. Consequently, agroforests must serve as a complement to, rather than a replacement for, remnant forests within tropical agricultural landscapes^55^.

### Managing ecosystem services and disservices

**Fig. 3:**
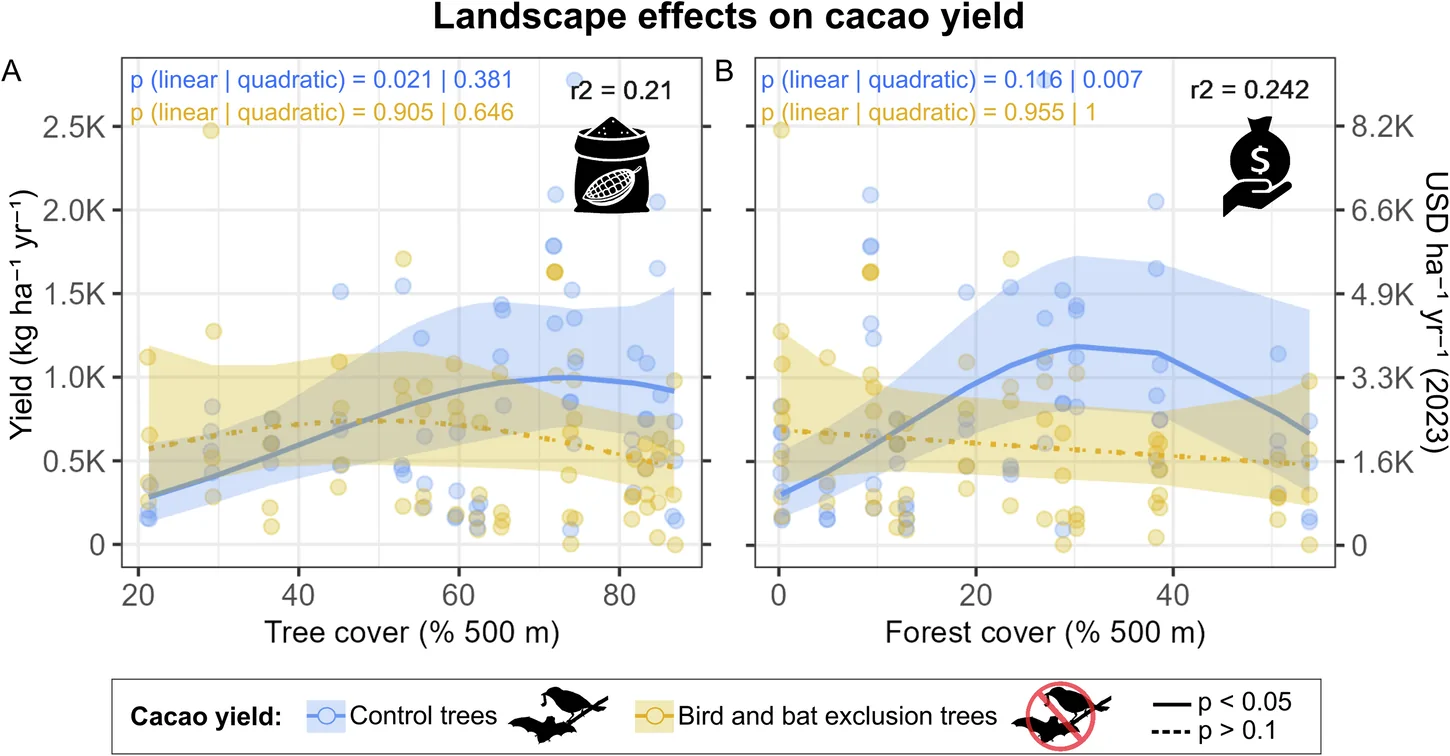
Landscape tree and forest cover shape cacao yields. Raw values and predicted trends with 95% CIs of annual dry cacao bean production in control trees (64 trees, blue dots and lines) and exclusion trees (64 trees, yellow dots and lines) across gradients of tree (**A**) and forest cover (**B**) in the landscape surrounding the agroforest. Tree-level yield values were multiplied by 1100 to scale them to hectares, assuming 3 × 3 m spacing between cacao trees. The monetary values in (B) represent the economic returns of the cacao yields according to global cacao prices in 2023 ($3.28 USD kg^−^^1^). *P*-values were generated using two-sided Wald *z*-tests and adjusted for multiple testing using the Bonferroni correction. The results of the yield models can be found in Supplementary Table 3.

Apart from moderating the shade-tree effects on biodiversity, landscape complexity boosted agroforest productivity. The predicted yield of open control trees increased from 265 kg ha^−^^1^ yr^−1^ at ≈20% tree cover in the surrounding landscape to ≈1000 kg ha^−1^ yr^−1^ at 70–80% (277% increase; Fig. 3A). Landscape forest cover had a unimodal relationship with yield outside exclusions, which peaked at 30–35% forest cover (≈1190 kg ha^−1^ yr^−1^; Fig. 3B). Conversely, cacao yield in exclusion trees did not significantly vary along the landscape gradients, indicating that landscape tree and forest cover increased cacao yields by enhancing bird- and bat-mediated pest control. We quantified these pest control services by calculating the difference between the average yields of control and exclusion trees in each agroforest and analyzed how this difference varied along the landscape gradients of tree and forest cover.

**Fig. 4:**
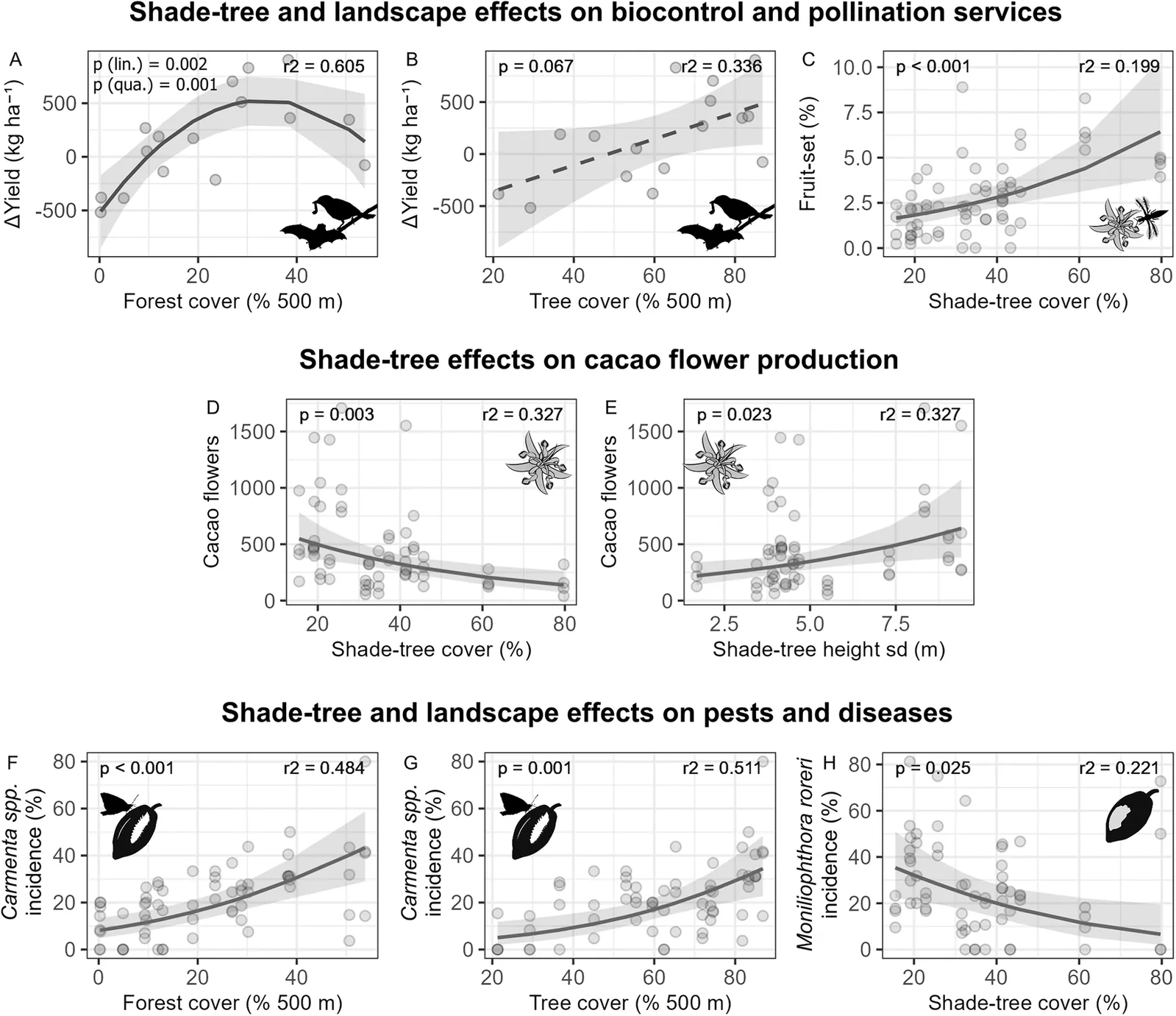
Shade-tree structure and landscape complexity moderate agroforest multifunctionality. We only represent trends and 95% CIs of significant (adjusted-*p* < 0.05; solid lines) or marginally significant (adjusted-*p* < 0.1; dashed lines) effects of shade-tree predictors included in the best-fitting GLMMs (Supplementary Tables 4 and 5). Changes in bird and bat contributions to cacao yield (difference between the average yield of control and exclusion trees; ∆yield) along landscape forest (**A**) and tree (**B**) cover gradients (*n* = 16 agroforests). Tree-level yield values were multiplied by 1100 to scale them to hectares, assuming 3 × 3 m spacing between cacao trees. Shade-tree cover effects on cacao fruit-set (used as a proxy of pollination services (**C**) and flower production (**D**) in the 64 control trees. **E** Shade-tree height heterogeneity (sd) effects on flower production in the 64 control trees. Landscape forest (**F**) and tree (**G**) effects on the proportion of cacao fruits affected by *Carmenta* spp. in the 64 control trees. **H** Shade-tree cover effects on the proportion of fruits affected by *Moniliophthora roreri* in the 64 control trees. *P*-values were generated using two-sided Wald *z*-tests and adjusted for multiple testing using the Bonferroni correction. Non-significant trends can be found in Supplementary Fig. 3.

Previous studies have reported positive effects of landscape forest cover on biological pest control and yields for agroforestry crops such as cacao^14^, coffee^56^, or macadamia^16^. We found that, in landscapes with no forest cover, the average yields were higher in exclusion than in control trees, with access of flying vertebrates to cacao trees resulting in a predicted loss of ≈500 kg ha^−^^1^ yr^−1^ (Fig. 4A). In deforested landscapes, pod predation by generalist frugivores in control trees—mainly parakeets, woodpeckers, and monkeys (according to local farmers’ knowledge)—and arthropod mesopredation in exclusion trees^19,42^ might explain the higher yields in the latter. As landscape-level forest cover increases, non-cacao food availability in the matrix rises, potentially reducing pod predation. Notably, average yields in control and exclusion trees converged at just 10% landscape forest cover, indicating that these ecosystem disservices can be offset with minimal forest conservation/restoration efforts. Higher forest cover may also facilitate the spillover of gleaning insectivorous birds and bats—many of which are forest-associated species^57,58^—into agroforests, ultimately enhancing arthropod suppression^59^. Indeed, previous studies in our study area demonstrate a progressive increase in the dominance of forest-associated insectivorous birds toward forested landscapes and lower feeding activity of aerial insectivorous bats with increasing prevalence of monocultures in the landscape^44,60^. This spillover of insectivores likely contributed to increasing yields in control trees, which nearly doubled those of exclusion trees in landscapes with 30–35% forest cover (≈97% increase; ≈500 kg ha^−1^ yr^−1^). Such bird and bat contributions to cacao yield, slightly lower than estimates in other Neotropical cacao agroforests^14^, represent a $1640 USD ha^−1^ annual boost to smallholder incomes^26^. However, as landscape forest cover exceeded 40%, bird and bat contributions to cacao yield declined. This trend may reflect a habitat dilution effect, where gleaning insectivores spread across the broader forest matrix rather than concentrating in agroforests^61^, potentially explaining the observed decrease in agroforest yield gains above 40% landscape forest cover. Therefore, these findings suggest that 30–35% forest cover in the landscape maximizes the economic contributions of birds and bats in cacao agroforests. Because these biocontrol services were independent of agroforest shade-tree structure (Fig. 2F–I), semi-forested landscapes may also benefit a wider variety of crops in the region. Disentangling the effects of landscape forest cover on the population dynamics of service and disservice providers, and their spillover into croplands, would advance our mechanistic understanding of these patterns and the optimization of trade-offs between ecosystem service and disservice in tropical landscapes.

Cacao fruit-set—quantified as the proportion of flowers that develop fruits and used as a proxy of the effectiveness of pollination services in agroforests^14^—was unaffected by landscape complexity but increased from 1.6% at 15% shade-tree cover to 6.4% at 80% (Fig. 4C), probably because of higher flower visitor abundance^22^. However, instead of boosting cacao yields, which declined by 76% along the shade-tree cover gradient (Fig. 2F), enhanced pollination in highly shaded agroforests helped compensate for the 75% reduction in flower production with increasing shade-tree cover (from 548 flowers at 15% cover to 139 at 80%; Fig. 4D). These contrasting patterns of fruit-set and flower production resulted in a net decrease of only 1.6% in the predicted number of flowers developing fruits in highly shaded agroforests compared to open ones. Therefore, the observed 76% yield decline with increasing shade-tree cover was most likely driven by light competition leading to fruit abortion during pod development^4^, as shaded conditions did not increase pest or disease incidence and had no negative effects on soil fertility and moisture (Fig. 1). Additionally, cacao flower production tripled with increasing shade-tree height heterogeneity (Fig. 4E). Combining shade trees of different heights creates vertical gaps in the agroforest canopy, allowing sunlight to reach the understory. This may help offset the negative effects of high shade-tree cover on flower production, highlighting further benefits of maintaining structurally heterogeneous canopies for enhanced biodiversity and productivity in agroforests.

To assess how shade-tree structure and landscape complexity influenced pest and disease incidence in control trees, we calculated the annual damage by *Carmenta* spp. (mainly *C. foraseminis*, Lepidoptera:Sessidae) and *Moniliophthora roreri* (Agaricales), two of the most important causes of cacao fruit losses in the Andean countries and the Neotropics, respectively^38^. *Carmenta* incidence rose from 5% of fruits damaged in simple landscapes (0% forest cover and 15% tree cover) to 35–45% in the most complex ones (55% forest cover and 85% tree cover; Fig. 4F, G). In contrast, *Moniliophthora* incidence was unaffected by landscape complexity but declined from 35% incidence at 15% shade-tree cover in the agroforests to 6.6% incidence at 80% cover (Fig. 4H). In both cases, the maximum predicted incidences were similar to those reported in other Neotropical regions^62–64^.

Because cacao yield was highest under low agroforest shade-tree cover and high landscape complexity, both patterns are consistent with the ‘resource concentration hypothesis’, predicting higher pest and disease pressure with increasing resource densities^65^. Similar positive relationships between cacao productivity and pest and disease incidence have been previously reported in our study area^66^. *Carmenta* species are lepidopterans that likely benefit from higher tree cover at the landscape scale for dispersal, including cacao plantations and alternative host trees, leading to greater impacts in complex landscapes. However, cacao yields were also highest in these landscapes, indicating that enhanced bird and bat arthropod suppression services outweighed the yield losses due to the increased *Carmenta* incidence.

On the other hand, greater tree cover may reduce wind flow and *Moniliophthora* dispersal^63^, potentially explaining why increased pod production in complex landscapes did not lead to higher *Moniliophthora* incidences. Although both species are currently restricted to the Neotropics, similar Lepidoptera (e.g., *Conopomorpha cramerella)* and fungi species (e.g., *Ceratobasidium theobromae*) pose major threats to cacao production in West Africa and Southeast Asia^17^. Thus, despite being locally derived, our findings may hold broader relevance for other tropical regions.

### Enhancing soil fertility and climate adaptation in agroforests

**Fig. 5:**
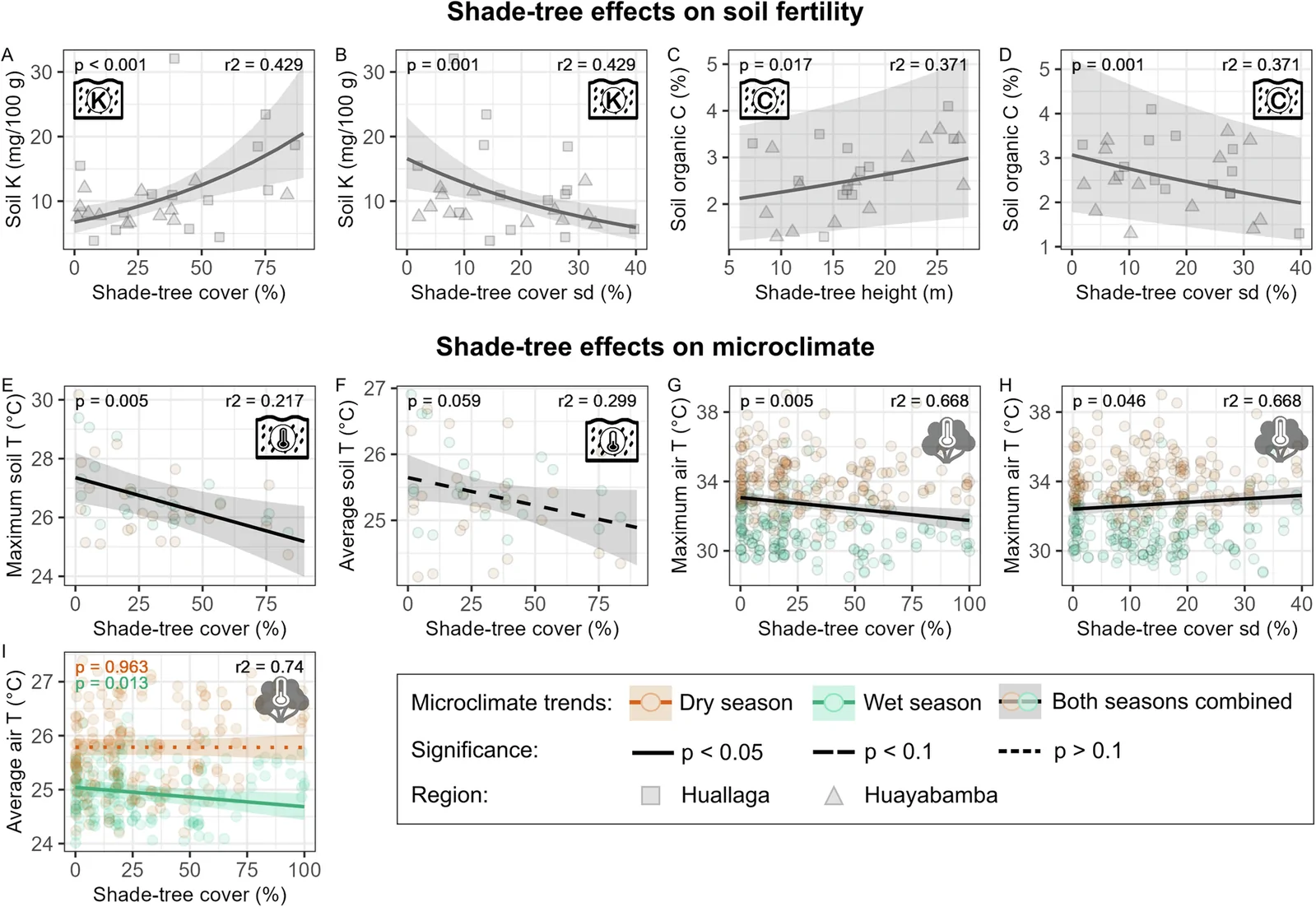
Shade trees influence soil fertility and microclimate. We represent trends and 95% CIs of significant (adjusted*-p* < 0.05) or marginally significant (adjusted*-p* < 0.1) effects of shade-tree predictors included in the best-fitting GLMMs (Supplementary Tables 6–8). Shade-tree predictors were measured in 20 × 20 m squares centered at the soil collection site, soil logger, or air temperature logger. To visualize potential geological differences between regions influencing shade-tree effects on soil fertility, we use squares to represent agroforests in the Huallaga River valley and triangles to represent those in the Huayabamba River valley. In case of significant interactions between microclimatic variables and season, we represent shade-tree effects in the dry (orange trends and CIs) and wet (green trends and CIs) seasons. Changes in agroforest soil potassium (K) content along the shade-tree cover (**A**) and shade-tree cover heterogeneity (sd; **B**) gradients. Changes in the proportion of organic carbon (C) in agroforest soils along the gradients in shade-tree height (**C**) and shade-tree cover heterogeneity (**D**). Soil composition was measured in 27 agroforests (one soil sample per agroforest). Variations in the daily maximum (**E**) and average (**F**) soil temperatures along the shade-tree cover gradient. Soil temperature was recorded in 25 agroforests. Seasonal changes in the daily maximum understory (air) temperatures along the shade-tree cover gradient (**G**) and with increasing shade-tree cover heterogeneity (**H**) in the dry season (August 2023; orange trends and points; *n* = 178 temperature records) and wet season (March 2023; green trends and points; *n* = 166 temperature records). **I** Variations in the daily average understory temperatures along the gradient in agroforest shade-tree cover. *P*-values were generated using two-sided Wald *z*-tests. Non-significant trends and elevation effects are shown in Supplementary Figs. 4 and 5.

It is often assumed that yield reductions in shaded agroforests are partly offset by improvements in soil fertility^24^. To assess shade-tree effects on soil fertility, we analyzed how agroforest shade-tree cover, cover heterogeneity, mean shade-tree height, and height heterogeneity influenced the pH and potassium, phosphorus, total nitrogen, and organic carbon contents in their soils. In line with previous studies reporting limited effects of shade trees on soil fertility^4,10^, pH and phosphorus and nitrogen contents were not affected by shade-tree structure (Fig. 1). Conversely, soil potassium content tripled with greater shade-tree cover (Fig. 5A), which is particularly relevant given potassium’s essential role in cacao nutrition and its typical scarcity in agroforest soils^67^. At higher shade-tree covers, there is enhanced input of nutrients from shade-tree leaf litter decomposition and potentially greater leaching of potassium from live leaf tissue of the shade trees, which may explain the increased potassium levels under closed-canopy conditions^67,68^. However, increases in shade-tree cover are paired with a decrease in heterogeneity as a result of more uniform canopies (Supplementary Fig. 1), potentially explaining the negative effects of shade-tree cover heterogeneity on soil potassium contents (Fig. 5B). Additionally, soil organic carbon rose from 2% to 3% with increasing shade-tree height (Fig. 5C), suggesting that maintaining tall shade trees in agroforests can potentially enhance carbon sequestration both above and below ground^3^. Tall shade trees typically have wide crowns and also produce more uniform canopies, which explains the negative relationship between carbon on the soil and shade-tree cover heterogeneity (Fig. 5D). Nevertheless, as localized shade-tree effects on soil composition are not necessarily translated into agroforest-level fertility gains^69^, these findings should be interpreted with caution.

Rising temperatures pose an important threat to agroforest sustainability worldwide^28–30^, and particularly in the Western Amazon^27^. As found in previous studies, our results show that shade trees moderate extreme temperatures in agroforests^4,66^. While higher shade-tree cover weakly reduced the average soil and understory temperatures (Fig. 5F–I), it decreased soil and understory maximum temperatures by 2.12 °C and 1.3 °C, respectively, in both the dry and wet seasons (Fig. 5E–G). Conversely, the maximum air temperatures rose by 0.8 °C with increasing cover heterogeneity, likely because gaps in the canopy allow sunlight to reach the agroforest understory, increasing its temperature (Fig. 5H).

Shaded conditions reduced agroforest temperatures but also cacao yields, highlighting an inherent trade-off for cacao production. The threshold at which the climatic benefits of shade begin to outweigh its negative effects on productivity is likely climate-dependent. In regions where heat stress is not yet a major constraint for cacao production (e.g., at higher elevations^27^), dense shade-tree cover currently offers limited climate-buffering advantage and primarily depresses yields. However, as temperatures rise under climate change, the adverse effects of low-shade conditions intensify^70^ progressively, making denser canopies more beneficial for maintaining both crop performance and microclimatic stability. Identifying how these interactive shade-tree and microclimate effects influence agroforest productivity across elevational and latitudinal gradients would help further define tailored management strategies to sustain tropical agroforests under climate change.

### Integrated agroforestry and landscape management

**Fig. 6:**
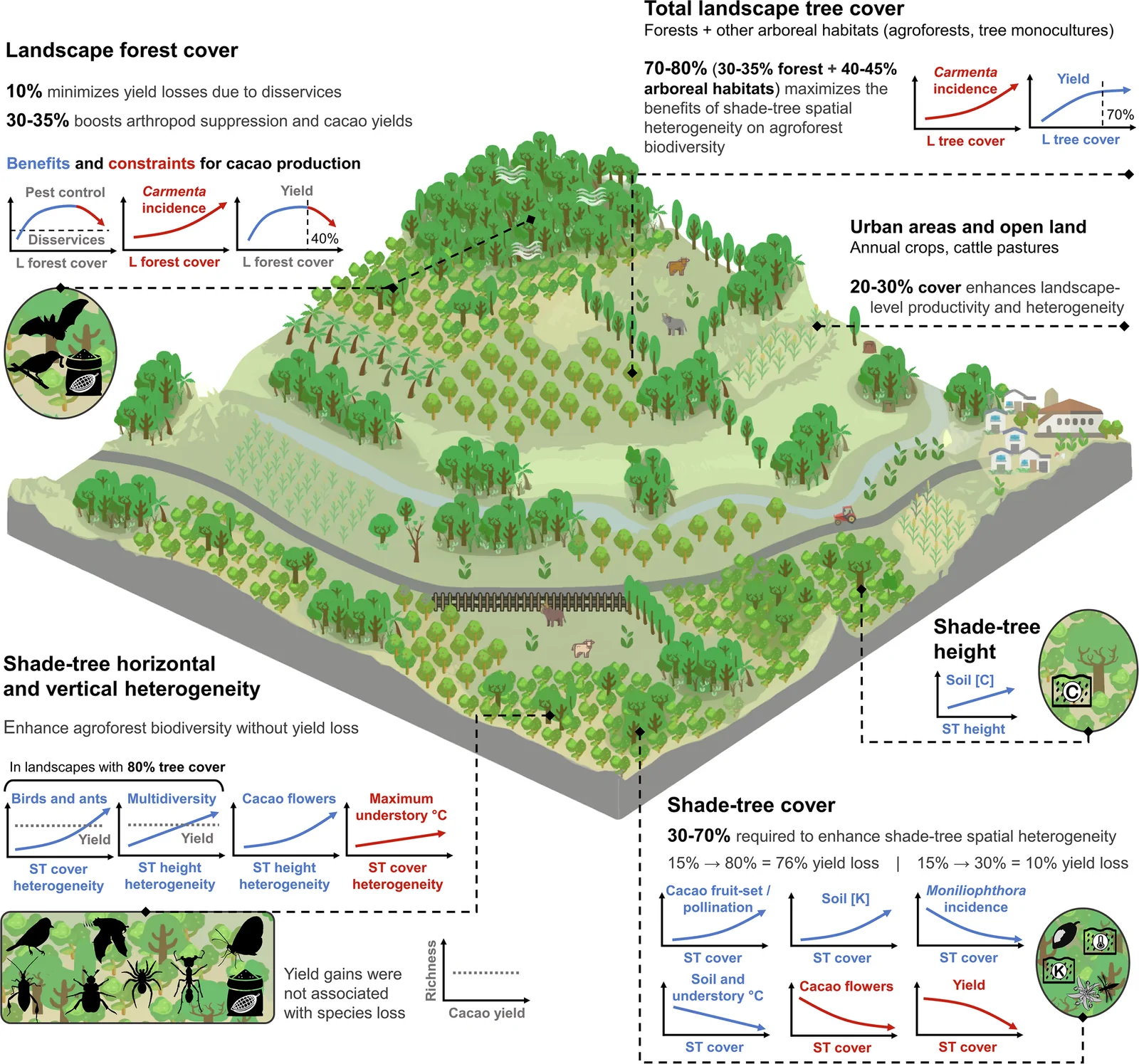
Integrated shade-tree and landscape management delivers biodiversity and yield win-wins. Maintaining a 30–35% forest cover in agricultural landscapes maximizes the contributions of birds and bats to cacao yields through their arthropod suppression services. An additional 40–45% cover of arboreal habitats other than forest (e.g., agroforestry systems and orchards; 70–80% total tree cover in the landscape) enhances landscape connectivity and the benefits of shade-tree spatial and vertical heterogeneity on biodiversity in agroforests. Depending on population density, dedicating the remaining 20–30% of the landscape to more productive land uses and urban areas would further increase landscape productivity and heterogeneity. At the agroforest level, promoting shade-tree spatial and vertical heterogeneity (e.g., combining clusters of shade trees of different heights and open areas within the agroforest) at 30% shade-tree cover enhances biodiversity with minimal yield losses. Additionally, maintaining tall shade trees promotes carbon sequestration and its storage in agroforest soils.

Ensuring the long-term sustainability of agroforestry systems and their benefits for biodiversity conservation, ecosystem service provision, and climate mitigation in tropical agricultural areas requires integrated management from the agroforest to the landscape^71^. Combining the protection of 30–35% of the forest cover with an additional 40–45% cover of other arboreal habitats (e.g., agroforests, orchards and scattered trees; 70–80% total tree cover) in the landscape could boost cacao yields due to enhanced arthropod suppression by flying vertebrates (Fig. 6). Dedicating the remaining 20–30% of the landscape to settlements and small patches with more intensive land uses (e.g., cattle pastures or annual crops) would further increase landscape productivity and heterogeneity, likely enhancing landscape-level species diversity^72,73^. Within this landscape structure, maintaining 30% local cover of shade trees organized in clusters with heterogeneous tree heights would maximize agroforestry benefits for biodiversity while sustaining their productivity. Moreover, this 30% shade-tree cover is not expected to substantially reduce soil moisture or limit the capacity of agroforests to adapt to rising temperatures^4^. Although we did not directly quantify carbon sequestration, previous studies have reported strong positive correlations with shade-tree cover in cacao agroforests^4^. Based on their results, maintaining 30% shade-tree cover would increase aboveground carbon by ≈200 t ha^−^^1^ relative to unshaded monocultures, further enhancing agroforest multifunctionality^3^.

Preserving the ecological and economic benefits of heterogeneous agroforest canopies over the long term requires active shade-tree replanting to offset losses from timber harvesting and natural mortality. Targeted replanting also offers opportunities for shade-tree diversification, which could help mitigate phytophagous insect damage through resource dilution while simultaneously promoting pest control and biodiversity conservation in agroforests by benefiting forest-associated insectivorous birds and bats^44,50,74^. Decision-support tools, such as diversityforagroforestry.org and cacaodiversity.org^75^, enable farmers and practitioners to identify optimal shade-tree species and cover levels tailored to specific management goals and local environmental conditions, facilitating shade-tree renewal and diversification.

Previous research^76^ suggests that, in regions requiring forest restoration, allocating 25% of the forest in a single large patch and distributing the remaining 75% across smaller, spatially dispersed patches could benefit forest-associated species and promote their spillover into croplands (Fig. 6). The above-mentioned landscape configuration sustains farmers’ livelihoods, wildlife, and locally valued forest resources such as wild meat and medicinal plants, crucial aspects for meeting several Sustainable Development Goals^11,77,78^. Additionally, such high landscape-scale tree and forest cover would also help mitigate climate change impacts by reducing land surface temperatures through evaporative cooling and enhancing carbon sequestration in agricultural landscapes^3,46^. However, our study focused on sparsely populated rural landscapes where built-up areas accounted for a maximum of 6.1% within 500 m buffers. While these landscapes are representative of many tropical cacao-growing regions, the proposed landscape designs may face spatial constraints in more densely populated areas or lose feasibility as rural communities undergo further development. In these cases, prioritizing smallholder arboreal crops over industrial monocultures would advance progress toward safeguarding ecosystem services and regional food security^6,11^.

Our study on Amazonian cacao agroforestry is a prime example of how promoting shade-tree spatial and vertical heterogeneity and landscape-scale forest protection can effectively reconcile biodiversity conservation and food production in tropical regions. This socio-ecological approach provides a promising pathway for the long-term sustainability of agroforestry in one of the world’s most biodiverse regions^6^. As megadiverse nations such as Peru transition toward more sustainable agricultural models, there is a growing need for robust empirical evidence to inform these emerging policy frameworks^8,11^. Incorporating this evidence into tailored environmental policies and collaboratively developed management protocols—engaging scientists, stakeholders, and local farmers—would support progress toward the Kunming–Montreal Global Biodiversity Framework and UN restoration targets.

## Materials and methods

### Study area

**Fig. 7:**
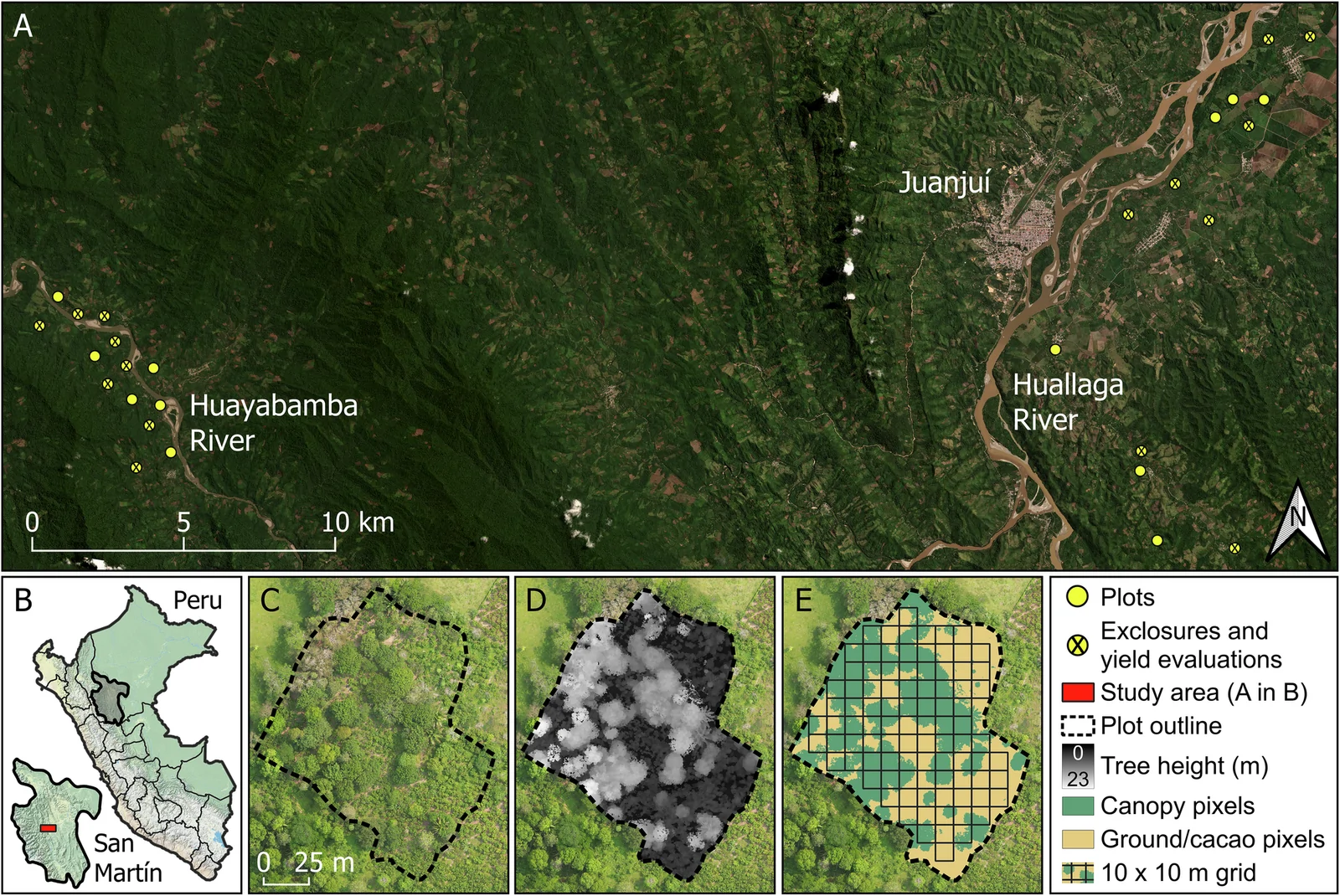
Map of the study area. Location of the 28 study agroforests in the northern Peruvian Amazon (**A**, **B**) and an example of a drone-acquired orthomosaic image (**C**) used to calculate local shade-tree height (**D**) and cover (**E**) of the agroforests. We calculated shade-tree cover heterogeneity as the standard deviation of shade-tree cover values in 10 × 10 m squares (**E**). The satellite image (**A**) corresponds to a PlanetScope image for August 2022 and was accessed via Planet Explorer^108^ (© Planet Labs PBC 2026. All rights reserved). The maps of Peru and San Martín (**B**) were built in QGIS 3.28.1 based on the ESRI World Physical Map (ESRI Inc.). Orthomosaics and digital elevation models (**C**–**E**) were built using Agisoft Metashape Pro v.2.0.0 (Agisoft LLC) by blending georeferenced drone images taken at 100 m above ground.

We developed, to our knowledge, the first integrated measurement of multi-taxa diversity, ecosystem services, pests and diseases, soil fertility, microclimate, and yield across 28 organic cacao agroforests in the northern Peruvian Amazon (San Martín Department; Fig. 7). The San Martín Department, responsible for 40% of the cacao production in Peru, has undergone a profound economic transformation over the past 30 years driven by alternative development policies aimed at replacing illicit coca cultivation with high-value agroforestry crops such as cacao and coffee^8^. The expansion of robust, legal agricultural markets and the development of farmer associations have successfully integrated smallholders into global value chains, catalyzing regional infrastructure development, improved public safety, and substantial rural poverty reduction^79^.

San Martín has two marked seasons: a dry season from April to September (24 mm of minimum monthly average precipitation) and a wet season from October to March (92 mm of minimum monthly average precipitation; data extracted from https://weatherspark.com). Our 28 agroforests were evenly distributed between the central Huallaga River valley and the middle-upper Huayabamba River valley (Supplementary Fig. 1). Both regions have similar mean annual temperatures (26 °C) and precipitation levels (1200–1400 mm), but differ in the agricultural intensity of the predominant land-use types and the characteristics of the remaining secondary forest. The Huallaga River valley has a greater proportion of intensive monocultures (e.g., rice and papaya), and its forest cover is mainly composed of seasonally dry tropical forests. On the other hand, the Huayabamba River valley—approximately 30 km away—is dominated by smallholder agroforests and banana plantations, with its forest cover composed of lowland tropical moist forests. Soils in both regions are classified as Orthic Acrisols, characterized by high acidity and low nutrient retention capacity^80^.

In each region, we selected agroforests following gradients of shade-tree canopy openness and landscape complexity, represented by the proportion of tree and forest cover. Elevations ranged from 256 to 358 m.a.s.l. in the Huallaga valley and from 330 to 436 m.a.s.l. in the Huayabamba. Agroforests varied from 0.3 to 2.3 ha in size, with mean areas of 1.29 ± 0.63 ha in the Huallaga valley and 1.29 ± 0.67 ha in the Huayabamba. The minimum distance between agroforests was 407 m, and all but three of them were spaced at least 500 m apart (mean distance to the nearest agroforests: 1093 m). All cacao agroforests were managed by smallholders affiliated with organic farmer cooperatives—the Cooperativa Agraria Cacaotera Acopagro Ltda. in the Huallaga valley and the Cooperativa Agraria Cacaotera Choba Choba Ltda. in the Huayabamba—both of which adhere to stringent organic and fair-trade standards^81^. Shade trees in the agroforests were dominated by native timber species planted by farmers, including mahogany (*Swietenia macrophylla*), capirona (*Calycophyllum spruceanum*), and bolaina (*Guazuma crinita*). Fruit trees such as avocado (*Persea americana*), papaya (*Carica papaya*), and *Citrus* spp., as well as non-native timber species like *Tectona grandis*, were also present but generally less abundant. Although shade timber trees are regularly harvested and used as a secondary income source in our study area, agroforest owners committed to abstaining from harvesting them to maintain a consistent agroforest canopy structure throughout the study period.

### Environmental variables

We used four variables to characterize agroforest canopy structure: shade-tree cover, cover heterogeneity, height, and height heterogeneity. For each agroforest, we built digital elevation models (DEMs) from drone images and measured shade-tree cover as the percentage of the agroforest covered by shade trees (pixels >7 m; Supplementary Fig. 1C–E). To calculate shade-tree cover heterogeneity, we divided canopy cover maps into 10 × 10 m sections (Supplementary Fig. 1E), measured the percentage of shade-tree cover in each section, and then calculated the standard deviation of shade-tree cover values across sections. Shade-tree height and height heterogeneity were measured as the average and standard deviation, respectively, of the maximum shade-tree height values in each 10 × 10 m section containing shade trees. DEMs were corrected to account for agroforest slopes before calculating shade-tree variables.

We estimated landscape complexity by calculating the percentages of landscape tree and forest cover in 500 m buffers from the agroforest perimeter. We extracted data on landscape tree cover using a 10 × 10 m resolution raster layer from ref. ^82^. The cover of mature, secondary, and riparian forests was estimated using satellite images extracted from PlanetScope for September 2022 on QGIS v3.28.1 and corrected through ground-truthing and drone images. Because dense agroforests are difficult to distinguish from forests in satellite imagery, the forest cover in this study may partially include highly shaded agroforests. Therefore, the main difference between the two landscape variables is the inclusion of scattered trees, tree monocrops, and low-shade agroforests in the landscape tree cover, but not in the forest cover.

We used the Pearson coefficient to calculate pairwise correlations between all environmental variables and additive models to analyze non-linear relationships between shade-tree cover and cover heterogeneity and between landscape tree and forest cover (Supplementary Table 9 and Supplementary Fig. 1).

### Biodiversity surveys and metrics

In each of the 28 cacao agroforests, we assessed the diversity of two vertebrate and five arthropod taxa, namely: birds, aerial insectivorous bats, ants, butterflies, spiders, Hemiptera species, and beetles. As highly mobile organisms, insectivorous birds and bats act as critical top-down regulators of arthropod populations, while frugivorous and nectarivorous species utilize agroforests as transient or complementary habitats, promoting forest regeneration through seed dispersal and pollination^36,58,83^. Additionally, arthropods such as ants and spiders, as well as predatory beetles and Hemiptera species, form a complex mesopredator network that may further regulate herbivore dynamics^37,42,84^. Conversely, specialized herbivores such as butterfly caterpillars, sap-sucking Hemiptera, and leaf-cutter ants represent lower-trophic guilds that can damage cacao tissues, flowers, and developing pods, potentially limiting agroforest productivity^38,74^. Therefore, the selected taxa serve as complementary ecological indicators for assessing how shade-tree configuration and landscape context shape agroforest biodiversity and multifunctionality.

#### Bird surveys

Bird surveys were conducted using 15-min point counts in 2022 (September–November) and 2023 (May–September). Each agroforest was visited eight times between 06:00 and 10:00 a.m., with all birds seen or heard within a 25-m radius from the agroforest center recorded^85^. To minimize temporal bias related to observation time, point counts were alternated between regions and times of day. Birds flying over the agroforests were excluded from the counts. All point counts were carried out by the same observer (P.A-L.).

Bird data was previously published in ref. ^44^. Here, we expand these analyses to assess the influence of understudied aspects of shade-tree spatial and vertical heterogeneity and identify management practices that potentially promote agroforest biodiversity without yield loss.

#### Bat surveys

We surveyed bats using ultrasonic bat detectors (Song Meter Mini Bat, Wildlife Acoustics, USA) positioned approximately 1.5 m above the ground in 2022 (August–November). While acoustic detectors are the standard methodology for surveying aerial insectivorous bats in the Neotropics^86^, they cannot reliably assess phyllostomid species richness due to high structural and frequency overlap in their echolocation calls^53^. Consequently, our acoustic assessments focus exclusively on the aerial insectivorous guild. Each agroforest was sampled over four full nights (17:30-06:30), divided into two rounds of two consecutive nights. To account for spatial heterogeneity within the plots, the detector locations were changed between sampling rounds. Sampling was repeated in cases of heavy rainfall. Bat detectors recorded in full spectrum at a sampling rate of 385 kHz. We programmed them to trigger on ultrasonic signals exceeding a minimum threshold of 12 kHz, with a five-second recording duration and a left-channel gain of 12 dB. Recordings were analyzed using Kaleidoscope Pro v.5.6.6 (Wildlife Acoustics, USA) to identify five-second audio files containing at least two bat echolocation calls with frequencies between 8 and 180 kHz, durations of 2–500 ms, and a maximum inter-pulse interval of 500 ms. All the recordings matching these characteristics were classified manually to the species/sonotype level following reference keys for Amazonian bats^87,88^.

As for birds, the data obtained from the bat surveys were previously published in ref. ^44^ and re-analyzed here to assess the potential of promoting shade-tree spatial and vertical heterogeneity in agroforests as a win-win strategy for biodiversity and farmers.

#### Ant surveys

We assessed tree-dwelling ants in six cacao trees in each agroforest using baits in 2023 (March–June). The baits, two pieces of tuna of about two cm^3^ and two small sponge cubes soaked in a 30–40% sugar solution, were placed on a plastic plate (25 cm diameter) fixed on a cacao tree at about 1.5 m height^89^. The baits were active for two hours, during which we collected up to five individuals per morphospecies for later identification. Ant observations were carried out from 06:00 to 18:00. Each tree was sampled twice, once in the morning and once in the afternoon, with visits to the same agroforests spaced two or three weeks apart. We treated these two-hour observations per tree as our sampling unit when estimating ant species richness (see “Species richness and multidiversity calculation” section). This made a total of 12 ant samples per agroforest, except for one that only had ten samples.

Previous comprehensive assessments of ant diversity in primary Amazonian rainforests revealed marked differences in assemblage composition between the forest canopy and ground^90^. Consequently, as we sampled ants only on cacao trees, our conclusions about shade-tree and landscape management effects on ant richness are restricted to this subset of the ant assemblage.

#### Butterfly surveys

Butterfly species richness in agroforests was assessed in 2022 (August to November) using Van Someren-Rydon traps baited with bananas fermented for ca. 48 h prior to deployment^91^. Each agroforest was sampled twice using four understory traps (ca. 80 cm above ground). We used two additional canopy traps (ca. 8 m above ground) during the first visit to account for possible differences in butterfly communities between vertical strata. Visits to the same agroforest were spaced by at least two weeks. Traps were at least 30 m apart from the nearest one and remained active for 24 h. Two agroforests were sampled for 48 h on the second visit due to continuous rain during the first 24-h period of the second visit. Captured butterflies were photographed for identification. Specimens not easily identifiable were collected for later identification.

#### Spider, Hemiptera, and beetle surveys

We used pyrethrum knockdown via canopy fogging to sample free-living arthropods from three pairs of mature cacao trees in each agroforest, following the procedure described in ref. ^92^. Sampling occurred twice—once in September–October 2022 and again in 2023. A Swingfog SN-50 fogging machine (Swingtec GmbH, Germany) was used with a 1% pyrethrum suspension in petroleum white oil to produce a thermal fog that dispersed the insecticide throughout the tree crowns. Fogging was performed for 5–10 min per tree pair, from 18:00 to 21:00 under still air conditions. Arthropods were collected on plastic sheets placed beneath the tree crowns and left in place for 30 min. The collected specimens were preserved in 96% ethanol for later identification.

We used CO1-5P (mitochondrial cytochrome oxidase 1) DNA metabarcoding to identify spider, Hemiptera, and beetle Operational Taxonomic Units (OTUs) in the canopy fogging samples. Sample preparation (homogenization of bulk samples), laboratory work (DNA extraction from homogenized samples, amplification and sequencing), and bioinformatic analyses (OTU identification) were performed by Advanced Identification Methods GmbH (AIM; Leipzig, Germany; https://www.metabarcoding.com) following the protocols described in ref. ^93^ and ref. ^94^. We used a geographically filtered South American Barcode of Life Data Systems (BOLD) reference database curated by AIM to identify our OTUs to the order level. This database was compiled by filtering the full public BOLD dataset (downloaded on July 4, 2025) to retain valid barcode records restricted to South American countries and geographic coordinates between −58° and 17° latitude, and −86° and −30° longitude. Records from Costa Rica were additionally integrated into the reference database due to its high density of barcode sequences within the BOLD repository. We compared OTUs identified in the samples with this geo-delimited dataset and kept those with a BOLD Grade ID of at least 85%, the minimum required to identify OTUs at the order level^93^. We then selected OTUs belonging to the orders Araneae (spiders), Hemiptera, and Coleoptera (beetles) and summed the number of reads per OTU and agroforest.

#### Species richness and multidiversity calculation

To correct for differences in sample completeness between agroforests (Supplementary Table 10), we pooled species records to estimate agroforest-level species richness for an equal abundance-based sample coverage between agroforests using the R package *iNEXT* ^95^. We refer to these estimated values as “species richness” for simplicity. Bird and bat data were pooled by taking the maximum number of individuals per species recorded during each visit to prevent overestimation of species abundances from repeated counts. For ants, we used sampling-unit-based incidence frequency data due to the lack of abundance data. Butterfly records were pooled by summing the total number of captures of each species in each agroforest. For spiders, Hemiptera species, and beetles, we summed the number of reads per OTU and agroforest and then reduced the number of singletons (OTUs with only one read) before estimating species richness values, following ref. ^93^. Reference sample coverages for the estimations were defined as the minimum sample coverage across all agroforests extrapolated to twice the sample sizes^96^ (Supplementary Table 2). Finally, we calculated agroforest-level multidiversity as the average of the scaled richness values of each taxon, obtained by dividing the estimated richness of each taxon in the agroforest by the maximum estimated richness of each taxon across all agroforests^97^.

### Exclusion experiments and cacao yield evaluations

To estimate bird and bat contributions to cacao yield, we set up exclusion experiments in 16 of the 28 agroforests (eight per region) selected to represent the gradients in shade-tree canopy structure and landscape complexity realized in each of the two regions. In each agroforest, we selected two pairs of contiguous cacao trees and built one exclosure for each pair. Therefore, each of the two exclosures covered two trees at the same time. Exclosures consisted of bamboo frames (6 m length, 2.5 m width, and 3.5 m height) covered with commercial fishing nets made of 0.40 mm invisible monofilament thread with a 2.5 cm mesh size. Exclosures were designed to block access by birds and bats to the treatment trees while permitting the entry of plant pathogens and arthropods, including potential insect pests^39^. In each agroforest, we selected two additional pairs of contiguous, non-enclosed trees as controls (*n* = 128 trees in total). By excluding bird and bat insectivores, we expected an increase in phytophagous arthropod damage to cacao leaves, flowers, and developing fruits in the exclusion trees, ultimately resulting in reduced harvestable yields compared to control trees^39^.

We monitored exclusion and control trees every two weeks from September 2022 to September 2023. In each monitoring visit, we (1) noted the number of healthy flowers (both open and developing flower buds) and developing fruits less than one cm long (ca. less than 15 days old) in a one-meter section of the trunk, (2) noted the number of fruits at different stages of development affected by different pests and diseases in the whole tree, (3) noted the number of healthy fruits at different stages of development in the whole tree; and (4) harvested all ripe healthy fruits. Healthy developing fruits were marked with plastic rings to avoid repeated counts, while those affected by pests and diseases were removed if they could no longer be harvested.

To transform the annual number of harvested pods per tree into weight measurements, we calculated the average dry weight of cacao beans in pods of different varieties. During the harvesting peak (June–August 2023), we (1) harvested 10 cacao pods from the most dominant cacao varieties in each agroforest, (2) fermented and sun-dried the contents of all pods of each variety separately, following the protocols of each cacao cooperative (6–7 days of fermentation and 2–3 days of drying), and (3) divided the total weight of the dry cacao beans by the number of pods in the sample. The most dominant varieties in the studied agroforests were: (1) Hybrid Huallaga, a mixture of the native Huallaga variety with commercial clones (60 pods harvested); (2) ICS-95 (40 pods harvested); (3) CYP-99 (40 pods harvested); and (4) mixtures of different native varieties (60 pods harvested^98^). We then multiplied the average dry weight of cacao beans per pod by the total number of pods harvested in each tree to calculate the kilograms of dry cacao produced by each tree throughout the year. The average dry weights of cacao beans per pod were calculated separately for each agroforest and cacao variety to account for genetic and spatial variability (e.g., differences in soil fertility and moisture between agroforests).

### Ecosystem services and pest and disease incidence

To analyze how shade-tree structure and landscape complexity affected ecosystem services in cacao agroforests, we focused on two key services potentially affecting cacao yield: pest control by birds and bats, and pollination^14^. We estimated bird and bat pest control services by assessing their contributions to cacao yield at the agroforest level. To do so, we calculated the difference between the average yield of the control and exclusion trees in each agroforest (∆yield).

Cacao pollination is a complex process involving intricate ecological interactions in which, despite the presumed importance of ceratopogonid midges, the precise identity of the primary pollinators remains a subject of ongoing debate^99,100^. Rather than analyzing the cascading effects of arthropod suppression by birds and bats on cacao pollination, we aimed to identify shade-tree and landscape management strategies promoting pollination under natural conditions. Therefore, we assessed pollination services in the agroforests based on the fruit-set of cacao control trees only. As cacao flowers cannot self-pollinate, cacao fruit-set is a good proxy for pollination services in agroforests^14^. To calculate cacao fruit-set, we divided the total number of developing fruits with less than one cm (i.e., fruits that started developing in the past 15 days) counted in the marked one-meter sections by the total number of flowers counted throughout the year in this same section. Cacao flowers only remain open for 48 h, ensuring that they are not counted twice on different visits. Note that cacao fruit-set might have been underestimated due to the loss of flowers that were successfully pollinated after one visit but lost before they could be counted as developing fruits on the next visit.

To assess shade-tree and landscape effects on cacao pests and diseases, we calculated the incidence of *Moniliophthora roreri* (frosty pod rot, Agaricales) and *Carmenta* spp. (mainly *C. foraseminis*, Lepidoptera:Sessidae). Both incidences were calculated as the total number of fruits damaged by each species divided by the total number of developing fruits counted in each control tree. We only evaluated flower production, fruit-set, and pests and diseases in control trees (*n* = 64) to avoid interference from the exclusion experiments.

### Agroforest soil fertility and microclimate

Soil fertility was assessed based on the pH and potassium, phosphorus, total nitrogen, and organic carbon contents of soil samples extracted at 0–-10 cm depth near the center of the agroforest. To evaluate the localized influence of shade-tree canopy structure on soil fertility, we collected a single soil sample per agroforest rather than a homogenized composite sample. This ensured the soil data remained paired with the canopy metrics quantified directly overhead. The studied agroforests were homogeneously fertilized in each region following advice from each cacao farmers’ cooperative. Soil samples were collected at least two months after applying organic fertilizers to avoid biases. One of the 28 plots was not sampled for soil fertility analyses due to logistical constraints. Composite samples (≈200 g) were dried in paper bags and analyzed at a commercial laboratory (Institut Dr. Nuss GmbH & Co. KG, Bad Kissingen, Germany) following the standard protocols of the Association of German Agricultural Analytic and Research Institutes^101^.

We used soil temperature and moisture loggers (TMS-4; TOMST, Czech Republic) to assess soil microclimatic variables (soil moisture and average and maximum daily temperature). One logger was placed near the center of each agroforest, recording temperature and moisture data every 15 min. Temperature (°C) was recorded at 8-cm depth, while moisture was measured at 0–14 cm depth using the time-domain transmission (TDT) method. Moisture values recorded with this method range from 100 (ambient air) to 3500 (distilled water^102^). Three of the loggers were damaged or lost during the study. Therefore, those agroforests were not included in the analyses of soil moisture and temperature.

Air temperature (°C) was measured using iButton DS1921H data loggers attached to cacao trees at ca. 1.5 m height. Initially, we placed one logger on each tree used in the yield evaluations and in those sampled with canopy fogging, making a total of eight loggers per agroforest in the 16 agroforests with exclusion experiments and 6 loggers per agroforest in the remaining 12 agroforests. However, due to logger loss or malfunction, the final number of loggers used per agroforest varied between four and eight (median = 6.5 loggers per agroforest). Air temperature measurements were taken every two hours.

To analyze how shade-tree management influenced agroforest microclimate across seasons, we calculated the average temperature and the average daily maximum temperature recorded by each logger in March and August 2023, the rainiest and driest months, respectively. For soil moisture, we used the mean records in each month. To relate soil composition and microclimate variables to shade-tree canopy structure, we used the digital elevation models to measure shade-tree cover, cover heterogeneity, height, and height heterogeneity in 20 × 20-m squares above each logger and soil collection site. For paired trees with temperature loggers (e.g., two exclusion trees inside the same bamboo cage), we measured shade-tree structure at the midpoint between the two trees.

### Statistical analyses

We used a multiple-testing approach to analyze shade-tree and landscape effects on biodiversity, ecosystem services, pest and disease incidence, and cacao yield. To assess biodiversity responses to shade-tree and landscape variables, we fitted eight generalized linear mixed models (GLMMs) for each of the eight response variables using Gamma distributions and logarithmic link functions for the seven richness variables, and beta distributions with logit link functions for multidiversity. Four of the eight models included one of the shade-tree structure variables (shade-tree cover, cover heterogeneity, height, or height heterogeneity) and its interaction with landscape tree cover, measured in the 500 m buffers around the agroforests. The remaining four models included each shade-tree variable and its interaction with landscape forest cover in the same buffers. Local and landscape predictors were scaled (mean = 0, sd = 1) before modeling. Region identity was included as a random effect. We compared each model to one with the same local and landscape variables but no interaction, and selected the one with the lowest Akaike Information Criterion corrected for small sample sizes (AICc) as the best-fitting one. Then, we compared the average marginal *R*^2^ of the four final models fitted with landscape tree cover and the average of the four models fitted with forest cover to identify the landscape variable that explained the greatest variability for each species richness response variable and multidiversity. In case of significant interactions between local shade-tree and landscape predictors in the best-fitting models, we projected the model to assess the influence of the shade-tree predictor on the response variable in landscapes with, on average, low tree or forest cover (40% tree cover or 6% forest cover; mean tree and forest cover minus one standard deviation) and in landscapes with high tree or forest cover (80% tree cover or 37% forest cover, mean tree and forest cover plus one standard deviation).

Shade-tree and landscape effects on cacao tree yield were analyzed using GLMMs with Gamma distributions and logarithmic link functions. We fitted two separate full models, each including either landscape tree cover or forest cover as a main predictor. Each model included the four scaled local variables (shade-tree cover, cover heterogeneity, height, and height heterogeneity) and their interactions with the treatment (control or exclusion trees), as well as the scaled landscape variable and its interaction with treatment. Additionally, we included the scaled quadratic effects of shade-tree cover and landscape tree and forest cover, and their two-way interactions with treatment, after preliminary visualizations of yield data distribution. Region was also included as a fixed effect to better represent differences in labor between the two cacao cooperatives. Tree-pair identity, nested within agroforest identity, was included as a random effect to account for spatial dependencies within agroforests. Before modeling, we transformed zeros into positive values by adding 0.0001 (0.1 g) in order to use Gamma distribution models. We removed shade-tree height from the full models after analyzing the multicollinearity between the predictors using the Variance Inflation Factor (VIF) in the R package *performance*^103^. We then used the ‘*dredge’* function in *MuMIn*^104^ to sequentially remove interactions and predictors from the full models, and selected the model with the lowest AICc as the best-fitting one. Quadratic terms were retained if ∆AICc <2 to identify potential shade-tree cover and landscape tree and forest cover thresholds affecting cacao yield^4^. Additionally, we assessed yield-biodiversity relationships by calculating the Pearson correlation coefficient between biodiversity variables and the average yield of the four control trees in each agroforest (64 cacao trees in total) to identify potential trade-offs between biodiversity and yield.

As none of the best-fitting yield models for cacao yield included interactions between shade-tree predictors and treatment (Supplementary Table 3), we only included landscape predictors in the Linear Mixed Models (LMMs) analyzing bird and bat contributions to cacao yield (agroforest-level ∆yield). One LMM included the linear term of landscape tree cover, while the other one included the linear and quadratic terms of landscape forest cover. Both models included region identity as a random effect. We report tree yield and ∆yield results multiplied by 1100 to scale tree-level yield to kilograms per hectare, assuming 1100 trees per hectare at a 3 × 3 m planting grid typical of the studied cacao agroforests^14^.

We analyzed local and landscape effects on cacao fruit-set, number of cacao flowers, and incidences of *Moniliophthora roreri* and *Carmenta* spp. using GLMMs. Models for fruit set and pest and disease incidence were fitted with beta distributions and logit link functions, while models for the number of cacao flowers were fitted using a negative binomial distribution. Before modeling, we transformed zeros into positive values by adding 0.0001 in order to use beta distribution models. We fitted two models for each of these response variables, one including the four shade-tree variables and landscape tree cover, and one with the shade-tree variables and landscape forest cover. Both models included tree-pair identity nested within agroforest and region identity as random effects. *Carmenta* models did not include tree-pair identity as a random effect to avoid model convergence issues derived from low random variance. After checking the collinearity between the predictors, we removed shade-tree height from the full models due to high VIF values (>6; Supplementary Table 11). We then used ‘*dredge*’ to perform model selection and chose the model with the lowest AICc as the best-fitting one.

Because landscape tree and forest cover were correlated with agroforest elevation (*r* = 0.74 for tree cover and 0.86 for forest cover), which can influence agroforest soil composition and microclimate, we did not assess the influence of landscape variables on soil fertility and microclimatic variables. Instead, these models only included the four shade-tree variables and agroforest elevation as a covariate to control for its potential effects. Models for proportions of soil organic carbon and total nitrogen were fitted with beta distributions and logit link functions. Models for soil pH, phosphorus, potassium, and moisture were fitted with Gamma distributions and log link functions, while soil and air temperature models were fitted with Gaussian distributions. Air and soil temperature models, as well as soil moisture models, included two-way interactions between each shade-tree variable and season, and between agroforest elevation and season. All soil composition and microclimate models included region identity as a random effect. Microclimate models also included agroforest identity as a random effect to account for the repeated measurements (one in each season). Additionally, air temperature models included logger and tree-pair identity as random effects. Again, we reduced the full models using ‘*dredge*’ and selected the model with the lowest AICc as the best-fitting one.

To correct for multiple testing, we adjusted the *p*-values of the best-fitting models using the Bonferroni method. For biodiversity models, we compared the AICc values of the four models fitted with landscape tree cover and those of the models fitted with forest cover and selected the landscape predictor with the lowest mean AICc to infer shade-tree and landscape effects on biodiversity variables. Therefore, we multiplied the *p*-values of species richness and multidiversity models by four to report the results. The *p*-values of the models analyzing ecosystem services, pests and diseases, cacao flowering, and yield were multiplied by two to correct for the use of different models to analyze landscape tree and forest cover effects. In this case, we used the model with the lowest AICc of the two to report the *p*-values and estimates of shade-tree effects.

All models were run using the R package *glmmTMB*^105^ in R v4.5.1^106^. Residual distributions were inspected using the package *performance*^103^, while their spatial autocorrelation was tested using Moran’s I test (package *ape*^107^; Supplementary Table 12).

## Supplementary information

Supplementary Information

Transparent Peer Review file

## Data Availability

The datasets generated during the study, as well as the numerical results of the models underlying the graphs and charts presented in Figs. 1–5, are publicly available at: https://doi.org/10.6084/m9.figshare.33173981.

## Code availability

The R code used for the statistical analyses performed in this study is publicly available at: https://doi.org/10.6084/m9.figshare.33173981.
