# Supplementary material for: Integrating local and landscape management promotes biodiversity, multifunctionality and yields in tropical agroforests
Source: Commun Earth Environ. 2026 Oct 30;7(1):840. doi: 10.1038/s43247-026-04141-3 (PMC13722579; doi:10.1038/s43247-026-04141-3)
Supplement: Supplementary file 1 [file 43247_2026_4141_MOESM1_ESM.pdf]

Supplementary Information

Integrating local and landscape management promotes biodiversity, multifunctionality and yields in tropical agroforests

Communications Earth & Environment

Pablo Aycart-Lazo, Blanca Ivañez-Ballesteros, Abed Alvino-Rosas, Daniel Ochterbeck, Melvin Opolka, Oscar Cabezas Huayllas, Stefan Dullinger, Evert Thomas, Teja Tscharntke, Ingolf Steffan-Dewenter, Carolina Ocampo-Ariza, Bea Maas.

Table of Contents

Supplementary Figure 1. Non-linear relationship between environmental variables 3

Supplementary Figure 2. Shade-tree and landscape effects on agroforest biodiversity............ 4

Supplementary Figure 3. Shade-tree and landscape effects on ecosystem services, disservices and cacao flower production in agroforests 5

Supplementary Figure 4. Shade-tree and landscape effects on agroforest soil fertility 6

Supplementary Figure 5. Shade-tree and landscape effects on agroforest microclimate 7

Supplementary Table 1. AICc and R^2 of GLMMs analyzing the effects of shade-tree and landscape variables on biodiversity 8

Supplementary Table 2. Results of the best-fitting models analyzing shade-tree and landscape effects on biodiversity............ 10

Supplementary Table 3. Results of the best-fitting models analyzing shade-tree and landscape effects on cacao yield............ 18

Supplementary Table 4. Results of the best-fitting models analyzing shade-tree and landscape effects on ecosystem services and cacao flowering in agroforests. 19

Supplementary Table 5. Results of the best-fitting models analyzing shade-tree and landscape effects on pest and disease incidence 20

Supplementary Table 6. Results of the best-fitting models analyzing shade-tree and landscape effects on soil fertility 21

Supplementary Table 7. Results of the best-fitting models analyzing shade-tree and landscape effects on agroforest microclimate (soil moisture, soil average temperature and soil maximum temperature). 22

Supplementary Table 8. Results of the best-fitting models analyzing the effects of shade-tree variables, agroforest elevation and season on agroforest microclimate 23

Supplementary Table 9. Pairwise Pearson correlation coefficients of environmental variables 24

Supplementary Table 10. Summary of biodiversity surveys 25

Supplementary Table 11. VIF values of the best-fitting models 26

Supplementary Table 12. Results of Moran´s I tests analyzing the spatial autocorrelation of the residuals of the best-fitting biodiversity models. 29

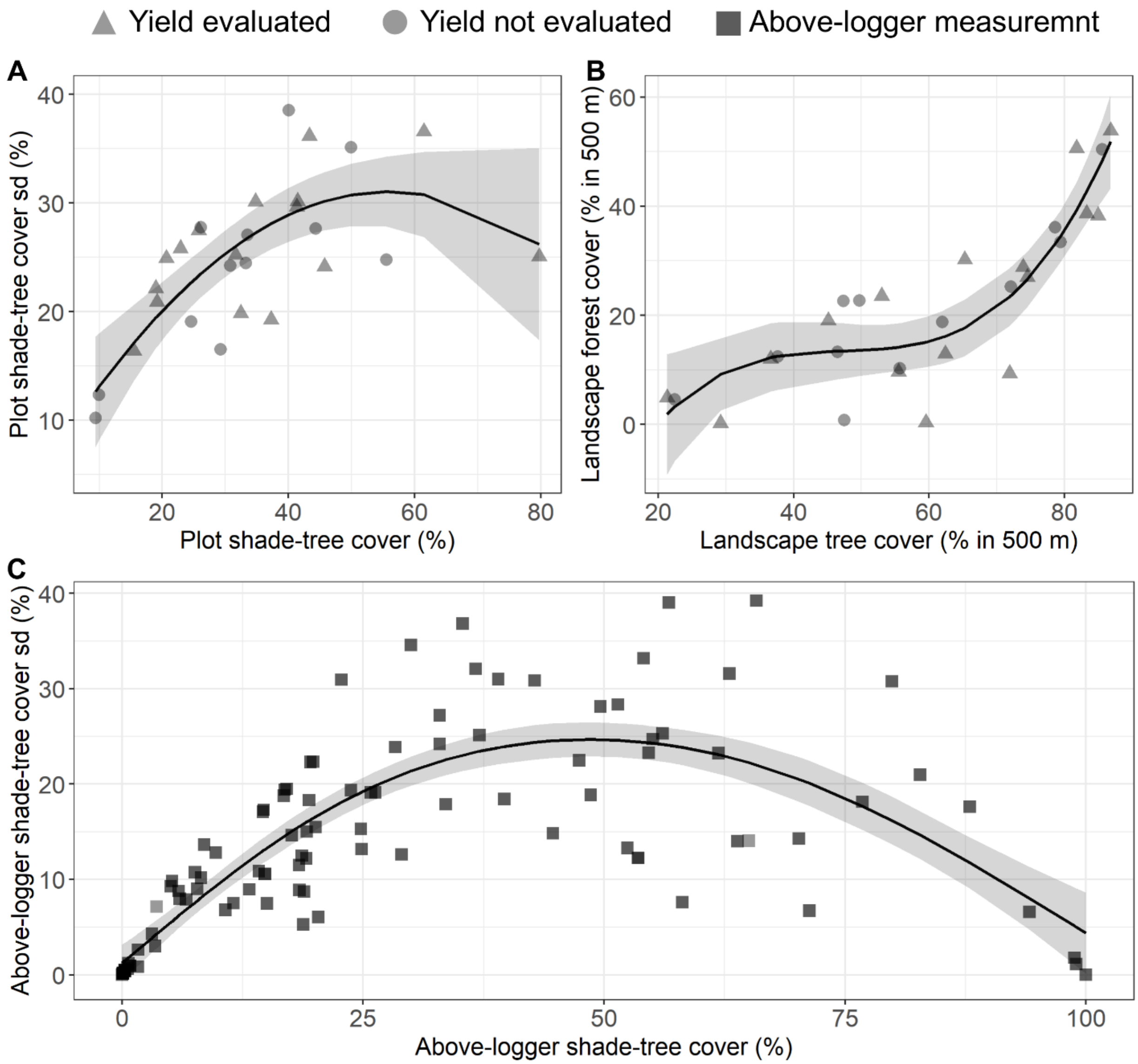

Supplementary Figure 1. Non-linear relationship between environmental variables**.** (A) Relationships between agroforest-level shade-tree cover and cover heterogeneity (sd). (B) Relationships between landscape tree and forest cover. (C) Relationships between shade-tree cover and cover heterogeneity in the 20 x 20 m squares used to measure agroforest shade-tree structure above the air temperature loggers.

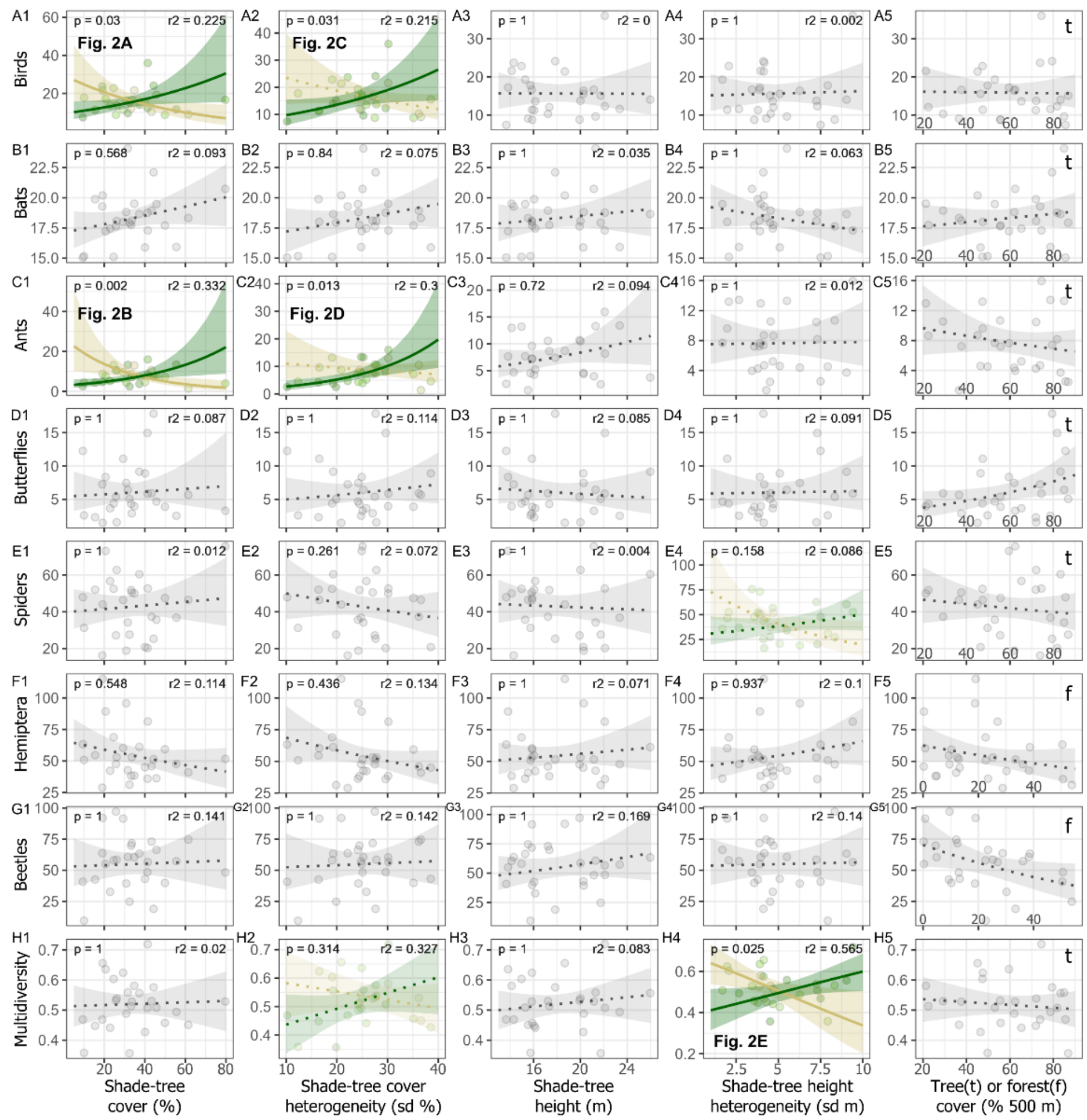

Supplementary Figure 2. Shade-tree and landscape effects on agroforest biodiversity. Trends of bird (A), bat (B), ant (C), butterfly (D), spider (E), Hemiptera (F) and beetle (G) species richness, and multidiversity (H) in cacao agroforests along shade-tree and landscape gradients. For biodiversity-environment relationships (measured in 28 plots), we represent the trends and 95% confidence intervals (CIs) predicted by the final models fitted with the landscape variable (either tree or forest cover) that resulted in the best model fit based on AICc values. Only models fitted with landscape tree cover included interactions between shade-tree and landscape variables in the final model (Supplementary Table 3). In these cases, we report the effects of the shade-tree variables at 40% (mean [60%] – one sd [20%]; yellow lines and CIs) and 80% (mean + one sd; green lines and CIs) tree cover. When the final model did not include this interaction, we report the effects of the shade-tree variables at the mean values of the landscape predictor (60% tree cover for birds, bats, ants, butterflies, spiders, and multidiversity; 20% forest cover for Hemiptera species and beetles; gray lines and CIs). Biodiversity trends along landscape gradients are the average of the four shade-tree models. We report p-values generated using two-sided Wald χ2 tests and adjusted for multiple testing using the Bonferroni correction. Figures showing significant trends are also included in the main text. Model results can be found in the Supplementary Table 2.

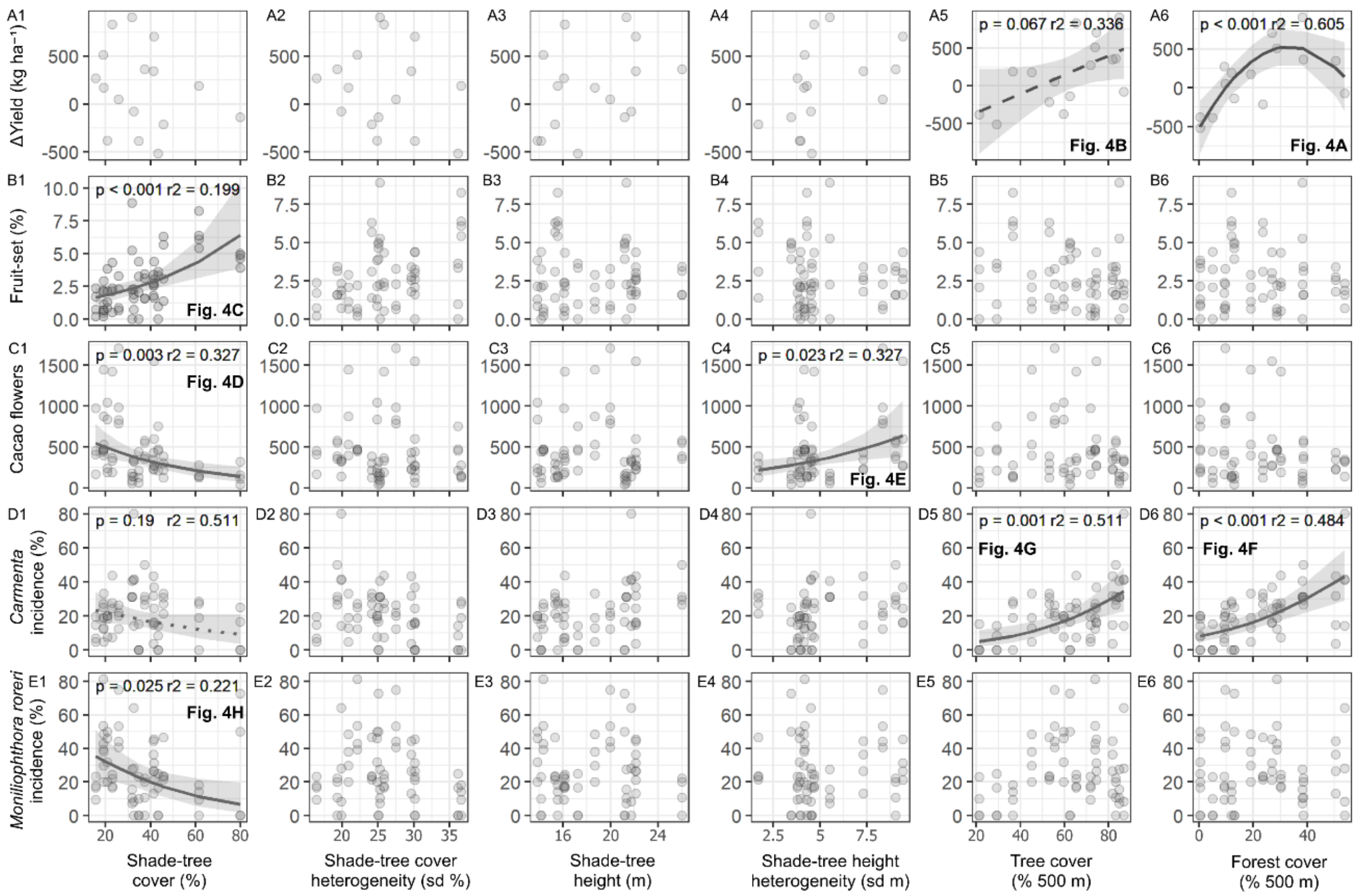

Supplementary Figure 3. Shade-tree and landscape effects on ecosystem services, disservices and cacao flower production in agroforests. Raw values and trends of ecosystem services, cacao flowering and pest and disease incidence in agroforests following gradients of shade-tree structure and landscape complexity. We only represent trends and 95% confidence intervals of the effects included in the best-fitting models. (A) Bird and bat contributions to cacao yield through arthropod suppression services (ΔYield). (B) Cacao fruit-set (proxy of pollination services). (C) Number of cacao flowers. (D) Percentage of cacao pods affected by *Carmenta* spp. (E) Percentage of cacao pods affected by *Moniliophthora roreri*. Solid lines indicate significant trends (adjusted-p < 0.05), dashed lines indicate marginally significant trends (adjusted-p < 0.1), and dotted lines indicate non-significant trends (adjusted-p > 0.1). P-values were generated using two-sided Wald z-tests and adjusted for multiple testing using the Bonferroni correction. Figures showing significant trends are also included in the main text. Model results can be found in the Supplementary Tables 4 and 5.

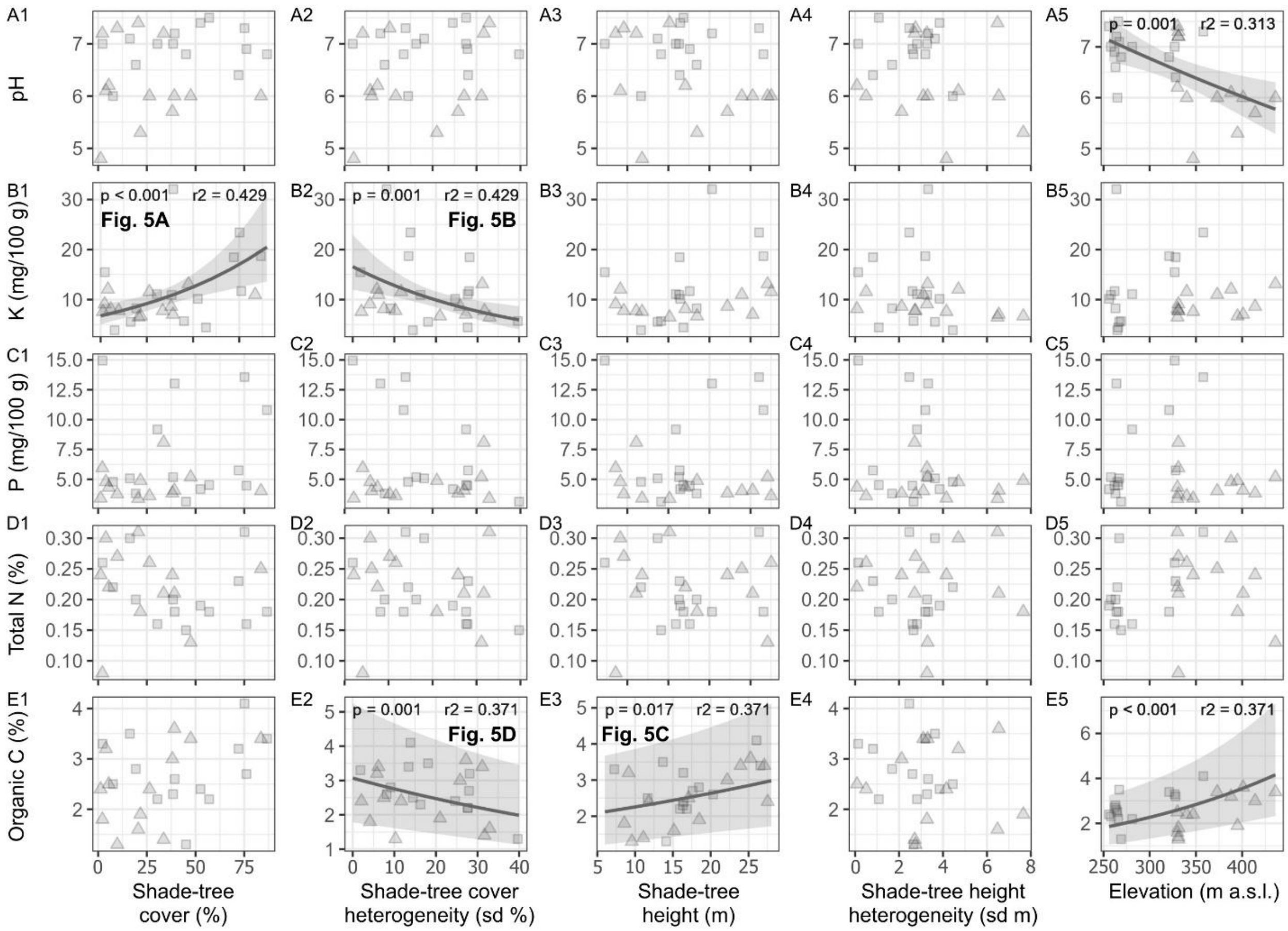

Supplementary Figure 4. Shade-tree and landscape effects on agroforest soil fertility. Raw values and trends of soil fertility indicators in agroforests following gradients of shade-tree structure and landscape complexity. Agroforest elevation was included as a predictor to control for its potential effects. To visualize potential geological differences between regions influencing shade-tree effects on soil fertility, we use squares to represent agroforests in the Huallaga River valley and triangles to represent those in the Huayabamba River valley. We only represent trends and 95% confidence intervals of the effects included in the best-fitting models. (A) Soil pH. (B) Soil potassium (K) content. (C) Soil phosphorus (P) content. (D) Soil total nitrogen (N) content. (E) Soil organic carbon (C) content. Solid lines indicate significant trends (adjusted-$p < 0.05$). *P*-values were generated using two-sided Wald z-tests and adjusted for multiple testing using the Bonferroni correction. Figures showing significant trends are also included in the main text. Model results can be found in the Supplementary Table 6.

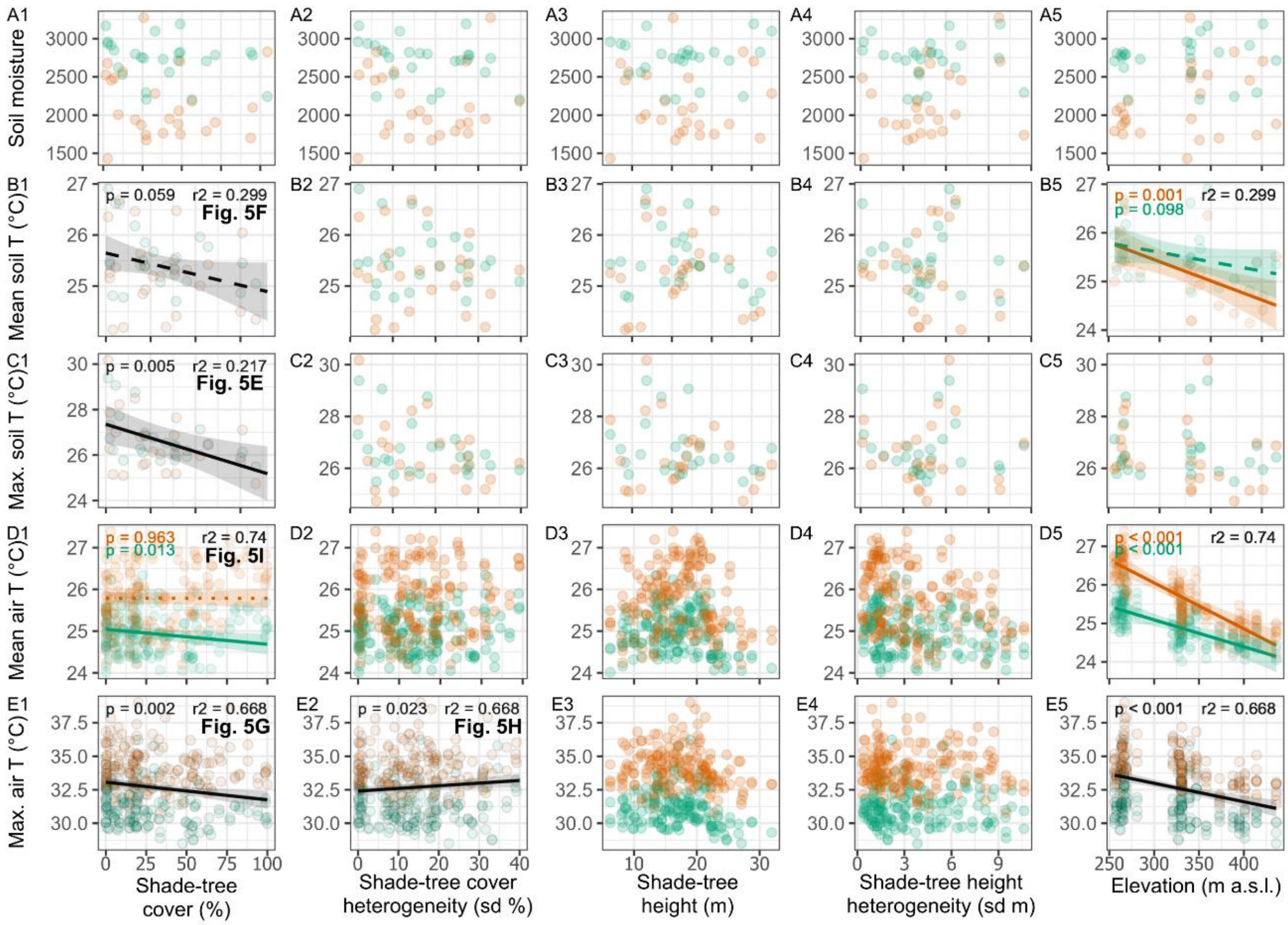

Supplementary Figure 5. Shade-tree and landscape effects on agroforest microclimate. Raw values and trends of microclimatic variables in agroforests following gradients of shade-tree structure and landscape complexity. Agroforest elevation was included as a predictor to control for its potential effects. We only represent trends and 95% confidence intervals of the effects included in the best-fitting models. Green points and trends represent microclimatic variables measured in the wet season (March 2023), while orange points and trends represent the values in the dry season (August 2023). (A) Soil moisture measured using the time-domain transmission (TDT) method. (B) Average soil temperature (T). (C) Maximum soil temperature. (D) Average understory (air) temperature. (E) Maximum understory (air) temperature. Solid lines indicate significant trends (adjusted-p < 0.05), dashed lines indicate marginally significant trends (adjusted-p < 0.1), and dotted lines indicate non-significant trends (adjusted-p > 0.1). P-values were generated using two-sided Wald z-tests and adjusted for multiple testing using the Bonferroni correction. Figures showing significant trends are also included in the main text. Model results can be found in the Supplementary Tables 7 and 8.

Supplementary Table 1. Values of Akaike Information Criterion corrected for small sample sizes (AICc) and marginal (marg.) R^2 of Generalized Linear Mixed Models (GLMMs) analyzing the effects of shade-tree and landscape variables on bird, bat, ant, and butterfly richness in cacao agroforests. We compared models with and without the two-way interactions between shade-tree and landscape variables and selected the one with lowest AICc (marked in bold). We report the results of the GLMMs fitted with the landscape variable that explained the greatest amount of variation (highest averaged R^2) for each response variable. Shade-tree cover sd and height sd represent shade-tree cover and height heterogeneities, respectively.

Response variable	Shade-tree variable	Two-way interaction	Landscape tree cover			Landscape forest cover		
			AICc	ΔAICc	R^2 (marg.)	AICc	ΔAICc	R^2 (marg.)
Bird richness	Shade-tree cover	no	187.43	3.56		**187.38**	**0**	**0.003**
		yes	**183.87**	**0**	**0.225**	188.57	1.19	
	Shade-tree cover sd	no	187.44	3.47		187.39	0.46	
		yes	**183.97**	**0**	**0.215**	**186.93**	**0**	**0.144**
	Shade-tree height	no	**187.44**	**0**	**0.000**	**187.38**	**0**	**0.003**
		yes	190.41	2.97		190.63	3.25	
	Shade-tree height sd	no	**187.39**	**0**	**0.002**	**187.36**	**0**	**0.003**
		yes	190.23	2.85		189.59	2.22	
Bat richness	Shade-tree cover	no	**126.79**	**0**	**0.093**	**127.64**	**0**	**0.067**
		yes	130.07	3.27		130.14	2.50	
	Shade-tree cover sd	no	**127.43**	**0**	**0.075**	128.72	0.42	
		yes	130.22	2.79		**128.30**	**0**	**0.146**
	Shade-tree height	no	**128.66**	**0**	**0.035**	**128.55**	**0**	**0.040**
		yes	131.48	2.83		131.45	2.90	
	Shade-tree height sd	no	**127.78**	**0**	**0.063**	**128.97**	**0**	**0.026**
		yes	131.02	3.24		132.24	3.27	
Ant richness	Shade-tree cover	no	164.64	6.12		164.45	3.13	
		yes	**158.52**	**0**	**0.332**	**161.33**	**0**	**0.249**
	Shade-tree cover sd	no	163.47	4.41		163.36	1.14	
		yes	**159.07**	**0**	**0.300**	**162.22**	**0**	**0.226**
	Shade-tree height	no	**162.82**	**0**	**0.094**	**162.68**	**0**	**0.102**
		yes	164.45	1.64		165.37	2.68	
	Shade-tree height sd	no	**164.70**	**0**	**0.012**	**164.53**	**0**	**0.023**
		yes	167.60	2.90		167.13	2.60	
Butterfly richness	Shade-tree cover	no	**153.53**	**0**	**0.087**	**154.95**	**0**	**0.024**
		yes	156.19	2.66		158.11	3.16	
	Shade-tree cover sd	no	**153.01**	**0**	**0.114**	**154.74**	**0**	**0.033**
		yes	155.97	2.96		157.96	3.22	
	Shade-tree height	no	**153.44**	**0**	**0.085**	**154.88**	**0**	**0.031**
		yes	156.68	3.24		157.92	3.04	
	Shade-tree height sd	no	**153.68**	**0**	**0.091**	154.97	1.53	**0.128**
		yes	154.87	1.19		**153.44**	**0**	

Supplementary Table 1 (continuation). Values AICc and marginal R^2 of Generalized Linear Mixed Models (GLMMs) analyzing the effects of shade-tree and landscape variables on the species richness of spiders, Hemiptera, beetles, and multidiversity in cacao agroforests.

Response variable	Shade-tree variable	Two-way interaction	Landscape tree cover			Landscape forest cover		
			AICc	ΔAICc	R^2 (marg.)	AICc	ΔAICc	R^2 (marg.)
Spider richness	Shade-tree cover	no	**242.83**	**0**	**0.012**	**242.79**	**0**	**0.007**
		yes	246.10	3.27		244.55	1.76	
	Shade-tree cover sd	no	**239.93**	**0**	**0.072**	**239.62**	**0**	**0.075**
		yes	243.15	3.22		242.37	2.76	
	Shade-tree height	no	**242.93**	**0**	**0.004**	**242.87**	**0**	**0.001**
		yes	245.56	2.63		246.14	3.27	
	Shade-tree height sd	no	242.94	1.20		242.70	0.53	
		yes	**241.74**	**0**	**0.086**	**242.17**	**0**	**0.094**
Hemiptera richness	Shade-tree cover	no	**250.13**	**0**	**0.060**	**248.46**	**0**	**0.114**
		yes	251.32	1.19		251.68	3.22	
	Shade-tree cover sd	no	**250.56**	**0**	**0.049**	**248.03**	**0**	**0.134**
		yes	252.39	1.83		251.28	3.25	
	Shade-tree height	no	**252.02**	**0**	**0.002**	**250.29**	**0**	**0.071**
		yes	252.89	0.87		252.28	1.99	
	Shade-tree height sd	no	**251.51**	**0**	**0.019**	**249.22**	**0**	**0.100**
		yes	254.07	2.56		251.84	2.62	
Beetle richness	Shade-tree cover	no	**262.45**	**0**	**0.117**	**261.74**	**0**	**0.141**
		yes	265.69	3.25		264.87	3.13	
	Shade-tree cover sd	no	**262.44**	**0**	**0.117**	**261.73**	**0**	**0.142**
		yes	265.66	3.22		264.86	3.13	
	Shade-tree height	no	**261.27**	**0**	**0.152**	**260.70**	**0**	**0.169**
		yes	264.52	3.24		263.97	3.27	
	Shade-tree height sd	no	**262.43**	**0**	**0.119**	**261.77**	**0**	**0.140**
		yes	263.24	0.81		264.77	3.00	
Multi-diversity	Shade-tree cover	no	**-48.07**	**0**	**0.020**	**-49.57**	**0**	**0.217**
		yes	-47.42	0.65		-48.16	1.41	
	Shade-tree cover sd	no	-48.09	0		**-49.55**	**0**	**0.215**
		yes	**-48.19**	**0.10**	**0.327**	-48.68	0.87	
	Shade-tree height	no	**-48.54**	**0**	**0.083**	**-50.75**	**0**	**0.319**
		yes	-45.50	3.04		-47.57	3.19	
	Shade-tree height sd	no	-48.07	4.36		**-49.84**	**0**	**0.242**
		yes	**-52.43**	**0**	**0.565**	-48.45	1.38	

Supplementary Table 2. Results of the best-fitting Generalized Linear Mixed Models (GLMMs) analyzing the effects of shade-tree variables and landscape tree cover on bird richness in cacao agroforests. We fitted one separate model for each shade-tree variable: shade-tree cover, cover heterogeneity, height and height heterogeneity. P-values were generated using two-sided Wald z-tests and adjusted for multiple testing using the Bonferroni correction. Shade-tree cover sd and height sd represent shade-tree cover and height heterogeneities, respectively. CI = 95% confidence interval.

		Predictors	Estimates	CI	p	adjusted-p
Bird richness	Shade-tree cover	(Intercept)	15.76	13.82 – 17.96	<0.001	<0.001
		Shade-tree cover	0.97	0.85 – 1.11	0.697	1
		Tree cover 500m	0.99	0.87 – 1.13	0.884	1
		Shade-tree cover — Tree cover 500m	1.29	1.07 – 1.56	0.008	0.032
		**Random Effects**				
		σ2	0.12			
		τ00 Region	0			
	Shade-tree cover sd	**Predictors**	**Estimates**	**CI**	**p**	**adjusted-p**
		(Intercept)	16.42	14.30 – 18.85	<0.001	<0.001
		Shade-tree cover sd	1.04	0.90 – 1.20	0.618	1
		Tree cover 500m	0.98	0.85 – 1.13	0.781	1
		Shade-tree cover sd — Tree cover 500m	1.21	1.05 – 1.40	0.008	0.032
		**Random Effects**				
		σ2	0.12			
		τ00 Region	0			
	Shade-tree height	**Predictors**	**Estimates**	**CI**	**p**	**adjusted-p**
		(Intercept)	15.64	13.55 – 18.06	<0.001	<0.001
		Shade-tree height	1	0.84 – 1.18	0.981	1
		Tree cover 500m	1	0.84 – 1.20	0.967	1
		**Random Effects**				
		σ2	0.14			
		τ00 Region	0			
	Shade-tree height sd	**Predictors**	**Estimates**	**CI**	**p**	**adjusted-p**
		(Intercept)	15.64	13.55 – 18.05	<0.001	<0.001
		Shade-tree height sd	1.02	0.88 – 1.18	0.825	1
		Tree cover 500m	1	0.85 – 1.17	0.96	1
		**Random Effects**				
		σ2	0.14			
		τ00 Region	0			

Supplementary Table 2 (continuation). Results of the best-fitting models analyzing the effects of shade-tree variables and landscape tree cover on bat richness in cacao agroforests.

		Predictors	Estimates	CI	p	adjusted-p
Bat richness	Shade-tree cover	(Intercept)	18.31	17.60 – 19.06	<0.001	<0.001
		Shade-tree cover	1.03	0.99 – 1.07	0.142	0.568
		Tree cover 500m	1.02	0.98 – 1.06	0.373	1
		**Random Effects**				
		σ2	0.01			
		τ00 Region	0			
	Shade-tree cover sd	**Predictors**	**Estimates**	**CI**	**p**	**adjusted-p**
		(Intercept)	18.32	17.59 – 19.07	<0.001	<0.001
		Shade-tree cover sd	1.03	0.98 – 1.08	0.21	0.84
		Tree cover 500m	1.03	0.98 – 1.07	0.258	1
		**Random Effects**				
		σ2	0.01			
		τ00 Region	0			
	Shade-tree height	**Predictors**	**Estimates**	**CI**	**p**	**adjusted-p**
		(Intercept)	18.32	17.58 – 19.09	<0.001	<0.001
		Shade-tree height	1.02	0.97 – 1.07	0.514	1
		Tree cover 500m	1.01	0.96 – 1.06	0.746	1
		**Random Effects**				
		σ2	0.01			
		τ00 Region	0			
	Shade-tree height sd	**Predictors**	**Estimates**	**CI**	**p**	**adjusted-p**
		(Intercept)	18.32	17.59 – 19.07	<0.001	<0.001
		Shade-tree height sd	0.97	0.93 – 1.02	0.266	1
		Tree cover 500m	1.02	0.98 – 1.07	0.274	1
		**Random Effects**				
		σ2	0.01			
		τ00 Region	0			

Supplementary Table 2 (continuation). Results of the best-fitting models analyzing the effects of shade-tree variables and landscape tree cover on ant richness in cacao agroforests.

Ant richness	Shade-tree cover	**Predictors**	**Estimates**	**CI**	**p**	**adjusted-p**
		(Intercept)	7.57	5.34 – 10.73	<0.001	<0.001
		Shade-tree cover	0.88	0.69 – 1.13	0.325	1
		Tree cover 500m	0.94	0.78 – 1.14	0.555	1
		Shade-tree cover— Tree cover 500m	1.62	1.23 – 2.11	<0.001	0.002
		**Random Effects**				
		σ2	0.2			
		τ00 Region	0.05			
	Shade-tree cover sd	**Predictors**	**Estimates**	**CI**	**p**	**adjusted-p**
		(Intercept)	8.05	6.66 – 9.75	<0.001	<0.001
		Shade-tree cover sd	1.2	0.97 – 1.47	0.088	0.352
		Tree cover 500m	0.91	0.75 – 1.09	0.307	1
		Shade-tree cover sd — Tree cover 500m	1.32	1.10 – 1.59	0.003	0.012
		**Random Effects**				
		σ2	0.21			
		τ00 Region	0			
	Shade-tree height	**Predictors**	**Estimates**	**CI**	**p**	**adjusted-p**
		(Intercept)	7.58	6.21 – 9.25	<0.001	<0.001
		Shade-tree height	1.19	0.92 – 1.53	0.18	0.72
		Tree cover 500m	0.85	0.66 – 1.08	0.185	0.74
		**Random Effects**				
		σ2	0.25			
		τ00 Region	0			
	Shade-tree height sd	**Predictors**	**Estimates**	**CI**	**p**	**adjusted-p**
		(Intercept)	7.63	6.06 – 9.61	<0.001	<0.001
		Shade-tree height sd	1.01	0.82 – 1.24	0.917	1
		Tree cover 500m	0.94	0.74 – 1.18	0.592	1
		**Random Effects**				
		σ2	0.27			
		τ00 Region	0.01			

Supplementary Table 2 (continuation). Results of the best-fitting models analyzing the effects of shade-tree variables and landscape tree cover on butterfly richness in cacao agroforests.

Response	Model	Predictors	Estimates	CI	p	adjusted-p
Butterfly richness	Shade-tree cover	**Predictors**	**Estimates**	**CI**	**p**	**adjusted-p**
		(Intercept)	5.99	4.33 – 8.28	<0.001	<0.001
		Shade-tree cover	1.07	0.83 – 1.39	0.599	1
		Tree cover 500m	1.2	0.90 – 1.59	0.221	0.884
		**Random Effects**				
		σ2	0.29			
		τ00 Region	0.03			
	Shade-tree cover sd	**Predictors**	**Estimates**	**CI**	**p**	**adjusted-p**
		(Intercept)	5.99	4.56 – 7.87	<0.001	<0.001
		Shade-tree cover sd	1.09	0.88 – 1.35	0.435	1
		Tree cover 500m	1.25	0.92 – 1.70	0.156	0.624
		**Random Effects**				
		σ2	0.28			
		τ00 Region	0.01			
	Shade-tree height	**Predictors**	**Estimates**	**CI**	**p**	**adjusted-p**
		(Intercept)	5.99	4.40 – 8.15	<0.001	<0.001
		Shade-tree height	0.93	0.73 – 1.19	0.574	1
		Tree cover 500m	1.23	0.92 – 1.64	0.17	0.68
		**Random Effects**				
		σ2	0.29			
		τ00 Region	0.03			
	Shade-tree height sd	**Predictors**	**Estimates**	**CI**	**p**	**adjusted-p**
		(Intercept)	6.01	4.53 – 7.97	<0.001	<0.001
		Shade-tree height sd	1	0.77 – 1.30	0.985	1
		Tree cover 500m	1.21	0.90 – 1.62	0.215	0.86
		**Random Effects**				
		σ2	0.29			
		τ00 Region	0.02			

Supplementary Table 2 (continuation). Results of the best-fitting models analyzing the effects of shade-tree variables and landscape tree cover on spider richness in cacao agroforests.

Response	Model	Predictors	Estimates	CI	p	adjusted-p
Spider richness	Shade-tree cover	(Intercept)	42.05	26.61 – 66.46	<0.001	<0.001
		Shade-tree cover	0.96	0.84 – 1.10	0.561	1
		Tree cover 500m	1.03	0.89 – 1.19	0.722	1
		**Random Effects**				
		σ2	0.12			
		τ00 Region	0.1			
	Shade-tree cover sd	**Predictors**	**Estimates**	**CI**	**p**	**adjusted-p**
		(Intercept)	41.79	25.80 – 67.69	<0.001	<0.001
		Shade-tree cover sd	0.87	0.76 – 1.01	0.065	0.26
		Tree cover 500m	0.99	0.86 – 1.14	0.905	1
		**Random Effects**				
		σ2	0.11			
		τ00 Region	0.11			
	Shade-tree height	**Predictors**	**Estimates**	**CI**	**p**	**adjusted-p**
		(Intercept)	42.1	27.99 – 63.31	<0.001	<0.001
		Shade-tree height	0.98	0.85 – 1.13	0.781	1
		Tree cover 500m	1.03	0.87 – 1.22	0.713	1
		**Random Effects**				
		σ2	0.12			
		τ00 Region	0.08			
	Shade-tree height sd	**Predictors**	**Estimates**	**CI**	**p**	**adjusted-p**
		(Intercept)	39.15	26.08 – 58.79	<0.001	<0.001
		Shade-tree height sd	0.96	0.82 – 1.12	0.585	1
		Tree cover 500m	1.02	0.88 – 1.18	0.799	1
		Shade-tree height sd — Tree cover 500m	1.21	1.01 – 1.45	0.04	0.16
		**Random Effects**				
		σ2	0.11			
		τ00 Region	0.08			

Supplementary Table 2 (continuation). Results of the best-fitting models analyzing the effects of shade-tree variables and landscape forest cover on Hemiptera richness in cacao agroforests.

		Predictors	Estimates	CI	p	adjusted-p
Hemiptera richness	Shade-tree cover	(Intercept)	54.29	48.09 – 61.30	<0.001	<0.001
		Shade-tree cover	0.91	0.81 – 1.03	0.137	0.548
		Forest cover 500m	0.92	0.81 – 1.05	0.21	0.84
		**Random Effects**				
		σ2	0.1			
		τ00 Region	0			
	Shade-tree cover sd	**Predictors**	**Estimates**	**CI**	**p**	**adjusted-p**
		(Intercept)	54.25	48.10 – 61.20	<0.001	<0.001
		Shade-tree cover sd	0.9	0.79 – 1.02	0.109	0.436
		Forest cover 500m	0.89	0.78 – 1.02	0.109	0.436
		**Random Effects**				
		σ2	0.1			
		τ00 Region	0			
	Shade-tree height	**Predictors**	**Estimates**	**CI**	**p**	**adjusted-p**
		(Intercept)	54.41	46.93 – 63.09	<0.001	<0.001
		Shade-tree height	1.05	0.92 – 1.20	0.499	1
		Forest cover 500m	0.9	0.75 – 1.09	0.276	1
		**Random Effects**				
		σ2	0.11			
		τ00 Region	0			
	Shade-tree height sd	**Predictors**	**Estimates**	**CI**	**p**	**adjusted-p**
		(Intercept)	54.37	48.08 – 61.48	<0.001	<0.001
		Shade-tree height sd	1.08	0.95 – 1.24	0.234	0.936
		Forest cover 500m	0.9	0.78 – 1.03	0.129	0.516
		**Random Effects**				
		σ2	0.1			
		τ00 Region	0			

Supplementary Table 2 (continuation). Results of the best-fitting models analyzing the effects of shade-tree variables and landscape forest cover on beetle richness in cacao agroforests.

Response	Model	Predictors	Estimates	CI	p	adjusted-p
Beetle richness	Shade-tree cover	(Intercept)	54.89	46.99 – 64.11	<0.001	<0.001
		Shade-tree cover	1.02	0.86 – 1.20	0.837	1
		Forest cover 500m	0.85	0.72 – 0.99	0.038	0.152
		**Random Effects**				
		σ2	0.16			
		τ00 Region	0			
	Shade-tree cover sd	**Predictors**	**Estimates**	**CI**	**p**	**adjusted-p**
		(Intercept)	54.89	46.99 – 64.10	<0.001	<0.001
		Shade-tree cover sd	1.02	0.85 – 1.22	0.818	1
		Forest cover 500m	0.85	0.72 – 1.00	0.055	0.22
		**Random Effects**				
		σ2	0.16			
		τ00 Region	0			
	Shade-tree height	**Predictors**	**Estimates**	**CI**	**p**	**adjusted-p**
		(Intercept)	54.73	46.98 – 63.75	<0.001	<0.001
		Shade-tree height	1.09	0.92 – 1.28	0.318	1
		Forest cover 500m	0.81	0.69 – 0.96	0.017	0.068
		**Random Effects**				
		σ2	0.16			
		τ00 Region	0			
	Shade-tree height sd	**Predictors**	**Estimates**	**CI**	**p**	**adjusted-p**
		(Intercept)	54.89	46.99 – 64.12	<0.001	<0.001
		Shade-tree height sd	1.01	0.86 – 1.19	0.888	1
		Forest cover 500m	0.84	0.71 – 1.00	0.046	0.184
		**Random Effects**				
		σ2	0.16			
		τ00 Region	0			

Supplementary Table 2 (continuation). Results of the best-fitting models analyzing the effects of shade-tree variables and landscape tree cover on multidiversity in cacao agroforests.

Response	Model	Predictors	Estimates	CI	p	adjusted-p
Multidiversity	Shade-tree cover	**Predictors**	**Estimates**	**CI**	**p**	**adjusted-p**
		(Intercept)	1.08	0.93 – 1.26	0.315	1
		Shade-tree cover	1.01	0.86 – 1.17	0.947	1
		Tree cover 500m	0.98	0.84 – 1.14	0.76	1
		**Random Effects**				
		σ2	0.03			
		τ00 Region	0			
	Shade-tree cover sd	**Predictors**	**Estimates**	**CI**	**p**	**adjusted-p**
		(Intercept)	1.13	0.97 – 1.31	0.126	0.504
		Shade-tree cover sd	1.03	0.90 – 1.19	0.643	1
		Tree cover 500m	0.97	0.84 – 1.13	0.708	1
		Shade-tree cover sd — Tree cover 500m	1.13	0.99 – 1.29	0.079	0.316
		**Random Effects**				
		σ2	0.03			
		τ00 Region	0			
	Shade-tree height	**Predictors**	**Estimates**	**CI**	**p**	**adjusted-p**
		(Intercept)	1.08	0.93 – 1.26	0.297	1
		Shade-tree height	1.05	0.91 – 1.22	0.488	1
		Tree cover 500m	0.95	0.81 – 1.12	0.555	1
		**Random Effects**				
		σ2	0.02			
		τ00 Region	0			
	Shade-tree height sd	**Predictors**	**Estimates**	**CI**	**p**	**adjusted-p**
		(Intercept)	1	0.88 – 1.14	0.99	1
		Shade-tree height sd	0.94	0.83 – 1.08	0.4	1
		Tree cover 500m	0.98	0.86 – 1.11	0.732	1
		Shade-tree height sd — Tree cover 500m	1.27	1.07 – 1.50	0.006	0.024
		**Random Effects**				
		σ2	0.02			
		τ00 Region	0			

Supplementary Table 3. Results of the best-fitting GLMMs analyzing the effects of shade-tree variables and landscape tree and forest cover on the annual yield (kg of dry cacao beans) of control and exclusion trees. P-values were generated using two-sided Wald z-tests and adjusted for multiple testing using the Bonferroni correction.

Response	Model	Predictors	Estimates	CI	p	adjusted-p
Cacao yield	Landscape tree cover	(Intercept)	0.81	0.58 – 1.13	0.208	0.416
		poly(Shade-tree cover)[1]	0.74	0.61 – 0.89	0.002	0.004
		poly(Shade-tree cover)[2]	0.85	0.70 – 1.04	0.122	0.244
		poly(Tree cover 500m)[1]	1.37	1.08 – 1.75	0.010	0.020
		poly(Tree cover 500m)[2]	0.84	0.65 – 1.09	0.190	0.380
		Treatment [Exclusion]	0.79	0.60 – 1.05	0.105	0.210
		Region [Huayabamba]	0.61	0.39 – 0.94	0.025	0.050
		poly(Tree cover 500m)[1] — Treatment [Exclusion]	0.66	0.50 – 0.88	0.004	0.008
		poly(Tree cover 500m)[2] — Treatment [Exclusion]	1.05	0.78 – 1.41	0.746	1.492
		**Random Effects**				
		σ2	0.44			
		τ00 Tree_pair:Agroforest_id	0.03			
		τ00 Agroforest_id	0.04			
	Landscape forest cover	**Predictors**	**Estimates**	**CI**	**p**	**adjusted-p**
		(Intercept)	0.83	0.58 – 1.20	0.327	0.654
		poly(Shade-tree cover)[1]	0.71	0.58 – 0.88	0.001	0.002
		poly(Shade-tree cover)[2]	0.9	0.75 – 1.09	0.280	0.56
		poly(Forest cover 500m)[1]	1.34	0.99 – 1.81	0.058	0.116
		poly(Forest cover 500m)[2]	0.71	0.57 – 0.89	0.003	0.006
		Treatment [Exclusion]	0.81	0.62 – 1.06	0.121	0.242
		Region [Huayabamba]	0.55	0.32 – 0.97	0.038	0.076
		poly(Forest cover 500m)[1] — Treatment [Exclusion]	0.67	0.51 – 0.87	0.003	0.006
		poly(Forest cover 500m)[2] — Treatment [Exclusion]	1.4	1.07 – 1.82	0.014	0.028
		**Random Effects**				
		σ2	0.43			
		τ00 Tree_pair: Agroforest_id	0.01			
		τ00 Agroforest_id	0.05			

Supplementary Table 4. Results of the best-fitting GLMMs analyzing the effects of shade-tree variables and landscape tree and forest cover on bird- and bat-mediated pest control (ΔYield), cacao fruit-set (proxy of pollination services), and cacao flowering in agroforests. P-values were generated using two-sided Wald z-tests and adjusted for multiple testing using the Bonferroni correction.

Response	Model	Predictors	Estimates	CI	p	adjusted-p
Bird and bat pest control (ΔYield)	Tree cover	(Intercept)	0.15	-0.03 – 0.33	0.111	0.222
		Tree cover 500m	0.23	0.02 – 0.45	0.034	0.068
		**Random Effects**				
		σ2	0.1			
		τ00 Region	0			
	Forest cover	**Predictors**	**Estimates**	**CI**	**p**	**adjusted-p**
		(Intercept)	0.15	0.02 – 0.28	0.019	0.038
		poly(Forest cover 500m)[1]	0.86	0.35 – 1.36	<0.001	0.002
		poly(Forest cover 500m)[2]	-0.88	-1.38 – -0.38	<0.001	0.001
		**Random Effects**				
		σ2	0.07			
		τ00 Region	0			
Pollination services (Fruit-set)		**Predictors**	**Estimates**	**CI**	**p**	**adjusted-p**
		(Intercept)	0.03	0.02 – 0.03	<0.001	<0.001
		Shade-tree cover	1.44	1.19 – 1.74	<0.001	<0.001
		**Random Effects**				
		σ2	0.39			
		τ00 Tree_pair:Agroforest_id	0.14			
		τ00 Agroforest_id	0			
		τ00 Region	0			
Cacao flowers		**Predictors**	**Estimates**	**CI**	**p**	**adjusted-p**
		(Intercept)	355.22	285.30 – 442.28	<0.001	<0.001
		Shade-tree cover	0.7	0.56 – 0.87	0.002	0.004
		Shade-tree height sd	1.35	1.07 – 1.69	0.011	0.022
		Forest cover 500m	0.82	0.65 – 1.03	0.09	0.18
		**Random Effects**				
		σ2				
		τ00 Tree_pair:Agroforest_id	0.19			
		τ00 Agroforest_id	0.05			
		τ00 Region	0			

Supplementary Table 5. Results of the best-fitting GLMMs analyzing the effects of shade-tree variables and landscape tree and forest cover on the incidence of *Carmenta* spp. and *Moniliophthora roreri* in cacao agroforests. P-values were generated using two-sided Wald z-tests and adjusted for multiple testing using the Bonferroni correction.

Response	Model	Predictors	Estimates	CI	p	adjusted-p
Carmenta spp. incidence	Tree cover	(Intercept)	0.22	0.16 – 0.29	<0.001	<0.001
		Shade-tree cover	0.75	0.54 – 1.05	0.095	0.19
		Tree cover 500m	2	1.34 – 2.98	<0.001	0.001
		**Random Effects**				
		σ2	-0.24			
		τ00 Agroforest_id	0.15			
		τ00 Region	0			
	Forest cover	**Predictors**	**Estimates**	**CI**	**p**	**adjusted-p**
		(Intercept)	0.22	0.16 – 0.30	<0.001	<0.001
		Shade-tree cover	0.68	0.50 – 0.93	0.017	0.034
		Forest cover 500m	1.93	1.44 – 2.60	<0.001	<0.001
		**Random Effects**				
		σ2	-0.24			
		τ00 Agroforest_id	0.19			
		τ00 Region	0			
Moniliophthora roreri incidence		**Predictors**	**Estimates**	**CI**	**p**	**adjusted-p**
		(Intercept)	0.29	0.19 – 0.44	<0.001	<0.001
		Shade-tree cover	0.59	0.39 – 0.89	0.012	0.024
		**Random Effects**				
		σ2	0.54			
		τ00 Tree_pair:Agroforest_id	0			
		τ00 Agroforest_id	0.43			
		τ00 Region	0			

Supplementary Table 6. Results of the best-fitting GLMMs analyzing the effects of shade-tree variables and agroforest elevation on soil fertility response variables (soil pH, soil potassium content and soil organic carbon content). The best-fitting models for soil phosphorus and nitrogen contents did not include any of the predictors analyzed. P-values were generated using two-sided Wald z-tests and adjusted for multiple testing using the Bonferroni correction.

	**Predictors**	**Estimates**	**CI**	**p**
Soil pH	(Intercept)	6.59	6.36 – 6.82	<0.001
	Elevation	0.94	0.91 – 0.97	0.001
		**Random Effects**		
	σ2	0.01		
	τ00 Region	0		
Soil potassium	**Predictors**	**Estimates**	**CI**	**p**
	(Intercept)	10.42	8.99 – 12.07	<0.001
	Shade-tree cover sd	0.76	0.64 – 0.89	0.001
	Shade-tree cover	1.39	1.17 – 1.66	<0.001
	**Random Effects**			
	σ2	0.14		
	τ00 Region	0		
Soil organic carbon	**Predictors**	**Estimates**	**CI**	**p**
	(Intercept)	0.03	0.02 – 0.04	<0.001
	Shade-tree height	1.1	1.02 – 1.19	0.017
	Shade-tree cover sd	0.89	0.82 – 0.95	0.001
	Elevation	1.28	1.14 – 1.44	<0.001
		**Random Effects**		
	σ2	0.03		
	τ00 Region	0.14		

Supplementary Table 7. Results of the best-fitting GLMMs analyzing the effects of shade-tree variables, agroforest elevation and season on agroforest microclimate (soil moisture, soil average temperature and soil maximum temperature). P-values were generated using two-sided Wald z-tests and adjusted for multiple testing using the Bonferroni correction.

	Predictors	Estimates	CI	p
Soil moisture	(Intercept)	2126.07	1877.64 – 2407.37	<0.001
	Season [wet]	1.28	1.18 – 1.39	<0.001
	**Random Effects**			
	σ2	0.02		
	τ00 Agroforest_id	0		
	τ00 Region	0.01		
Average soil temperature	**Predictors**	**Estimates**	**CI**	**p**
	(Intercept)	25.25	25.02 – 25.48	<0.001
	Shade-tree cover	-0.2	-0.41 – 0.01	0.059
	Elevation	-0.4	-0.63 – -0.17	0.001
	Season [wet]	0.27	0.07 – 0.47	0.008
	Elevation — season [wet]	0.2	-0.00 – 0.41	0.05
	**Random Effects**			
	σ2	0.15		
	τ00 Agroforest_id	0.2		
	τ00 Region	0		
Maximum soil temperature	**Predictors**	**Estimates**	**CI**	**p**
	(Intercept)	26.61	25.97 – 27.25	<0.001
	Shade-tree cover	-0.57	-0.97 – -0.18	0.005
	**Random Effects**			
	σ2	0.34		
	τ00 Plot	0.71		
	τ00 Region	0.14		

Supplementary Table 8. Results of the best-fitting GLMMs analyzing the effects of shade-tree variables, agroforest elevation and season on agroforest microclimate (average and maximum air temperature). P-values were generated using two-sided Wald z-tests and adjusted for multiple testing using the Bonferroni correction.

	Predictors	Estimates	CI	p
Average air temperature	(Intercept)	25.78	25.68 – 25.88	<0.001
	Shade-tree cover	0	-0.08 – 0.07	0.963
	Elevation	-0.64	-0.83 – -0.46	<0.001
	Season [wet]	-0.85	-0.91 – -0.79	<0.001
	Shade-tree cover — season[wet]	-0.13	-0.21 – -0.06	0.001
	Elevation — season[wet]	0.27	0.21 – 0.33	<0.001
		**Random Effects**		
	σ2	0.1		
	τ00 Tree_code	0		
	τ00 Tree_pair	0.02		
	τ00 Agroforest_id	0.05		
	τ00 Region	0		
Maximum air temperature	**Predictors**	**Estimates**	**CI**	**p**
	(Intercept)	34.2	33.92 – 34.47	<0.001
	Shade-tree cover	-0.36	-0.59 – -0.13	0.002
	Shade-tree cover sd	0.2	0.03 – 0.38	0.021
	Elevation	-0.74	-1.01 – -0.47	<0.001
	Season [wet]	-3.15	-3.36 – -2.95	<0.001
		**Random Effects**		
	σ2	0.88		
	τ00 Tree_code	0.01		
	τ00 Tree_pair	0.22		
	τ00 Agroforest_id	0.33		
	τ00 Region	0		

Supplementary Table 9. Pairwise Pearson correlation coefficients of environmental variables in the 28 plots and the subset of 16 plots with evaluations of ecosystem services, pests and diseases and cacao yield.

Variable 1	Variable 2	Pearson r (all plots)	Pearson r (yield plots)
Shade-tree cover (%)	Shade-tree cover sd (%)	0.592	0.477
Shade-tree cover (%)	Shade-tree height (m)	0.375	0.255
Shade-tree cover (%)	Shade-tree height sd (m)	-0.254	-0.070
Shade-tree cover (%)	Landscape tree cover (% 500 m)	-0.088	-0.185
Shade-tree cover (%)	Landscape forest cover (% 500 m)	-0.097	-0.049
Shade-tree cover sd (%)	Shade-tree height (m)	0.140	-0.181
Shade-tree cover sd (%)	Shade-tree height sd (m)	-0.054	0.103
Shade-tree cover sd (%)	Landscape tree cover (% 500 m)	-0.336	-0.580
Shade-tree cover sd (%)	Landscape forest cover (% 500 m)	-0.321	-0.327
Shade-tree height (m)	Shade-tree height sd (m)	0.451	0.710
Shade-tree height (m)	Landscape tree cover (% 500 m)	0.477	0.595
Shade-tree height (m)	Landscape forest cover (% 500 m)	0.433	0.611
Shade-tree height sd (m)	Landscape tree cover (% 500 m)	0.348	0.397
Shade-tree height sd (m)	Landscape forest cover (% 500 m)	0.352	0.331
Landscape tree cover (% 500 m)	Landscape forest cover (% 500 m)	0.797	0.773

Supplementary Table 10. Summary of biodiversity surveys, number of species detected and sample coverages for each taxon in the 28 cacao agroforestry systems samples. Reference sample coverages were used to estimate the species richness of each taxon for an equal sample coverage between plots.

Taxon	Sampling method	Visits per plot	Total samples per plot	Total richness	Sample coverage (%; mean & SD)	Reference sample coverage (%)
Birds	15-minute point counts	8	8 point counts	97 species	65.96 (12.07)	53.12
Bats	Full-night ultrasonic recordings	4	4 nights	26 species/ sonotypes	99.7 (0.32)	99.66
Ants	Sugar and tuna baits on cacao trees	2	12* cacao trees	74 species	73.76 (12.96)	63.00
Butterflies	Canopy and understory baited traps	2	10 traps	71 species	78.93 (13.17)	57.53
Spiders	Fogging of cacao trees & metabarcoding	2	12 cacao trees	435 OTUs	99.98 (0.03)	99.98
Hemiptera	Fogging of cacao trees & metabarcoding	2	12 cacao trees	808 OTUs	99.99 (0.01)	99.99
Beetles	Fogging of cacao trees & metabarcoding	2	12 cacao trees	1031 OTUs	99.97 (0.05)	99.93

Supplementary Table 11. Variance Inflation Factor (VIF) values of the final models analyzing shade-tree and landscape effects on bird, bat, ant and butterfly species richness. LTC = landscape tree cover; stc = shade-tree cover; stcsd = shade-tree cover heterogeneity; sth = shade-tree height; sthsd = shade-tree height heterogeneity.

Model	Term	VIF	SE factor
Birds ~ stc : LTC	Shade-tree cover	1.017	1.008
	Tree cover 500m	1.014	1.007
	Shade-tree cover : Tree cover 500m	1.012	1.006
Birds ~ stcsd : LTC	Shade-tree cover sd	1.154	1.074
	Tree cover 500m	1.147	1.071
	Shade-tree cover sd : Tree cover 500m	1.048	1.024
Birds ~ sth + LTC	Shade-tree height	1.366	1.169
	Tree cover 500m	1.366	1.169
Birds ~ sthsd + LTC	Shade-tree height sd	1.154	1.074
	Tree cover 500m	1.154	1.074
Bats ~ stc + LTC	Shade-tree cover	1.008	1.004
	Tree cover 500m	1.008	1.004
Bats ~ stcsd + LTC	Shade-tree cover sd	1.123	1.060
	Tree cover 500m	1.123	1.060
Bats ~ sth + LTC	Shade-tree height	1.316	1.147
	Tree cover 500m	1.316	1.147
Bats ~ sthsd + LTC	Shade-tree height sd	1.141	1.068
	Tree cover 500m	1.141	1.068
Ants ~ stc : LTC	Shade-tree cover	1.111	1.054
	Tree cover 500m	1.028	1.014
	Shade-tree cover : Tree cover 500m	1.088	1.043
Ants ~ stcsd : LTC	Shade-tree cover sd	1.130	1.063
	Tree cover 500m	1.157	1.076
	Shade-tree cover sd : Tree cover 500m	1.049	1.024
Ants ~ sth + LTC	Shade-tree height	1.489	1.220
	Tree cover 500m	1.489	1.220
Ants ~ sthsd + LTC	Shade-tree height sd	1.085	1.042
	Tree cover 500m	1.085	1.042
Butterflies ~ stc + LTC	Shade-tree cover	1.007	1.004
	Tree cover 500m	1.007	1.004
Butterflies ~ stcsd + LTC	Shade-tree cover sd	1.096	1.047
	Tree cover 500m	1.096	1.047
Butterflies ~ sth + LTC	Shade-tree height	1.055	1.027
	Tree cover 500m	1.055	1.027
Butterflies ~ sthsd + LTC	Shade-tree height sd	1.012	1.006
	Tree cover 500m	1.012	1.006

Supplementary Table 11 (continuation). Variance Inflation Factor (VIF) values of the final models analyzing shade-tree and landscape effects on spider, Hemiptera, and beetle species richness and agroforest multidiversity. LTC = landscape tree cover; LFC = landscape forest cover; stc = shade-tree cover; stcsd = shade-tree cover heterogeneity; sth = shade-tree height; sthsd = shade-tree height heterogeneity.

Model	Term	VIF	SE factor
Spiders ~ stc + LTC	Shade-tree cover	1.020	1.010
	Tree cover 500m	1.020	1.010
Spiders ~ stcsd + LTC	Shade-tree cover sd	1.061	1.030
	Tree cover 500m	1.061	1.030
Spiders ~ sth + LTC	Shade-tree height	1.258	1.122
	Tree cover 500m	1.258	1.122
Spiders ~ sthsd : LTC	Shade-tree height sd	1.269	1.126
	Tree cover 500m	1.071	1.035
	Shade-tree height sd : Tree cover 500m	1.192	1.092
Hemiptera ~ stc + LFC	Shade-tree cover	1.007	1.004
	Forest cover 500m	1.007	1.004
Hemiptera ~ stcsd + LFC	Shade-tree cover sd	1.123	1.060
	Forest cover 500m	1.123	1.060
Hemiptera ~ sth + LFC	Shade-tree height	1.124	1.060
	Forest cover 500m	1.124	1.060
Hemiptera ~ sthsd + LFC	Shade-tree height sd	1.183	1.088
	Forest cover 500m	1.183	1.088
Beetles ~ stc + LFC	Shade-tree cover	1.006	1.003
	Forest cover 500m	1.006	1.003
Beetles ~ stcsd + LFC	Shade-tree cover sd	1.105	1.051
	Forest cover 500m	1.105	1.051
Beetles ~ sth + LFC	Shade-tree height	1.205	1.098
	Forest cover 500m	1.205	1.098
Beetles ~ sthsd + LFC	Shade-tree height sd	1.155	1.075
	Forest cover 500m	1.155	1.075
Multidiversity ~ stc + LTC	Shade-tree cover	1.053	1.026
	Tree cover 500m	1.053	1.026
Multidiversity ~ stcsd + LTC	Shade-tree cover sd	1.059	1.029
	Tree cover 500m	1.036	1.018
	Shade-tree cover sd : Tree cover 500m	1.034	1.017
Multidiversity ~ sth + LTC	Shade-tree height	1.204	1.097
	Tree cover 500m	1.204	1.097
Multidiversity ~ sthsd : LTC	Shade-tree height sd	1.325	1.151
	Tree cover 500m	1.145	1.070
	Shade-tree height sd : Tree cover 500m	1.171	1.082

Supplementary Table 11 (continuation). Variance Inflation Factor (VIF) values of the final models analyzing shade-tree and landscape effects on cacao yield, cacao flower production, *Carmenta* spp. incidence, soil fertility and microclimate. Response variables not included in the table had fewer than two predictors in their final models.

Model	Term	VIF	SE factor
Cacao yield (landscape tree cover)	poly(Shade-tree cover, 2)	1.885	1.373
	poly(Tree cover 500m, 2)	4.773	2.185
	Treatment	1.011	1.005
	Region	1.711	1.308
	poly(Tree cover 500m, 2) : Treatment	3.162	1.778
Cacao yield (landscape forest cover)	poly(Shade-tree cover, 2)	1.799	1.341
	poly(Forest cover 500m, 2)	5.347	2.312
	Treatment	1.003	1.002
	Region	2.726	1.651
	poly(Forest cover 500m, 2) : Treatment	2.665	1.632
Cacao flowers	Shade-tree cover	1.006	1.003
	Shade-tree height sd	1.120	1.058
	Forest cover 500m	1.117	1.057
Carmenta spp. Incidence (landscape tree cover)	Shade-tree cover	1.079	1.039
	Tree cover 500m	1.079	1.039
Carmenta spp. Incidence (landscape forest cover)	Shade-tree cover	1.000	1.000
	Forest cover 500m	1.000	1.000
Soil K	Shade-tree cover	1.234	1.111
	Shade-tree cover sd	1.234	1.111
Soil organic C	Shade-tree cover sd	1.035	1.017
	Shade-tree height	1.348	1.161
	Elevation	1.313	1.146
Soil mean temperature	Shade-tree cover	1.012	1.006
	Elevation	1.254	1.120
	Season	1.000	1.000
	Elevation : Season	1.243	1.115
Understory mean temperature	Shade-tree cover	1.172	1.083
	Season	1.002	1.001
	Elevation	1.037	1.018
	Shade-tree cover : Season	1.257	1.121
	Season : Elevation	1.124	1.060
Understory maximum temperature	Shade-tree cover	1.165	1.080
	Shade-tree cover sd	1.109	1.053
	Elevation	1.054	1.027
	Season	1.000	1.000

Supplementary Table 12. Results of the two-sided Moran´s I tests analyzing the spatial autocorrelation of the residuals of the best-fitting biodiversity models.

Response variable	Shade-tree variable	Observed	Expected	sd	p-value
Bird richness	Shade-tree cover sd	-0.007	-0.037	0.038	0.425
	Shade-tree cover	-0.006	-0.037	0.036	0.392
	Shade-tree height sd	-0.010	-0.037	0.037	0.464
	Shade-tree height	-0.010	-0.037	0.037	0.461
Bat richness	Shade-tree cover sd	-0.008	-0.037	0.038	0.452
	Shade-tree cover	-0.006	-0.037	0.038	0.407
	Shade-tree height sd	-0.008	-0.037	0.038	0.446
	Shade-tree height	-0.014	-0.037	0.038	0.548
Ant richness	Shade-tree cover sd	-0.039	-0.037	0.039	0.957
	Shade-tree cover	-0.017	-0.037	0.037	0.601
	Shade-tree height sd	-0.043	-0.037	0.039	0.877
	Shade-tree height	-0.047	-0.037	0.039	0.791
Butterfly richness	Shade-tree cover sd	-0.023	-0.037	0.038	0.712
	Shade-tree cover	-0.020	-0.037	0.038	0.662
	Shade-tree height sd	-0.023	-0.037	0.038	0.709
	Shade-tree height	-0.022	-0.037	0.038	0.692
Spider richness	Shade-tree cover sd	-0.010	-0.037	0.039	0.490
	Shade-tree cover	-0.010	-0.037	0.039	0.486
	Shade-tree height sd	-0.013	-0.037	0.038	0.527
	Shade-tree height	-0.009	-0.037	0.039	0.476
Hemiptera richness	Shade-tree cover sd	-0.018	-0.037	0.037	0.610
	Shade-tree cover	-0.010	-0.037	0.038	0.474
	Shade-tree height sd	-0.024	-0.037	0.036	0.723
	Shade-tree height	-0.021	-0.037	0.037	0.659
Beetle richness	Shade-tree cover sd	-0.008	-0.037	0.038	0.455
	Shade-tree cover	-0.007	-0.037	0.038	0.442
	Shade-tree height sd	-0.009	-0.037	0.038	0.472
	Shade-tree height	-0.008	-0.037	0.039	0.444
Multidiversity	Shade-tree cover sd	-0.025	-0.037	0.038	0.745
	Shade-tree cover	-0.024	-0.037	0.037	0.736
	Shade-tree height sd	-0.028	-0.037	0.038	0.816
	Shade-tree height	-0.026	-0.037	0.038	0.781

Supplementary Table 12 (continuation). Results of the two-sided Moran´s I tests analyzing the spatial autocorrelation of the residuals of the best-fitting models for cacao yield, bird and bat contributions to yield (ΔYield), cacao fruit-set, number of cacao flowers, *Carmenta* spp. and *Moniliophthora roreri* incidences, soil fertility and microclimatic variables. The best-fitting models for soil phosphorus and nitrogen contents and moisture did not include any shade-tree variables and were therefore excluded from this analysis.

Response variable	Landscape variable	Observed	Expected	sd	p-value
Cacao yield	Tree cover 500m	-0.007	-0.067	0.066	0.365
	Forest cover 500m	-0.011	-0.067	0.065	0.389
Δyield	Tree cover 500m	-0.047	-0.067	0.068	0.770
	Forest cover 500m	-0.038	-0.067	0.065	0.657
Fruit-set	None	-0.027	-0.067	0.066	0.547
Cacao flowers	None	-0.023	-0.067	0.061	0.477
Carmenta spp. incidence	Tree cover 500m	-0.068	-0.067	0.060	0.982
	Forest cover 500m	-0.181	-0.067	0.062	0.065
Moniliophthora roreri incidence	None	-0.032	-0.067	0.049	0.474
Soil potassium	None	-0.012	-0.038	0.039	0.489
Soil pH	None	-0.030	-0.038	0.037	0.819
Soil organic carbon	None	-0.011	-0.038	0.039	0.480
Soil moisture	None	-0.007	-0.042	0.044	0.428
Average soil temperature	None	-0.006	-0.042	0.045	0.422
Maximum soil temperature	None	-0.016	-0.042	0.043	0.545
Average air temperature	None	-0.027	-0.037	0.038	0.800
Maximum soil temperature	None	-0.008	-0.037	0.031	0.347
