# Supplementary material for: Integrating local and landscape management promotes biodiversity, multifunctionality and yields in tropical agroforests
Source: Commun Earth Environ. 2026 Oct 30;7(1):840. doi: 10.1038/s43247-026-04141-3 (PMC13722579; doi:10.1038/s43247-026-04141-3)
Supplement: Supplementary file 2 [file 43247_2026_4141_MOESM2_ESM.pdf]

nature portfolio

# Peer Review File

# Integrating local and landscape management promotes biodiversity, multifunctionality and yields in tropical agroforests

Corresponding Author: Dr Pablo Aycart Lazo

**This file contains all reviewer reports in order by version, followed by all author rebuttals in order by version.**

Version 0:

Reviewer comments:

Reviewer #1

(Remarks to the Author)
This is an interesting work where the authors comprehensively assess the ecosystem services in cacao agroforests. including biodiversity, pest and disease incidence protection, microclimate control, soil fertility and cacao yield. I found it very complete and engaging for a complex topic and a good integrative multi-scale design. I have some suggestions mainly to improve the readability of the paper.
My main suggestion is to include some text, probably in supplementary material, about the social history of the 2 cooperatives in order to qualify claims of the type "Yield gains were not associated with species loss" which occur under current social and economic constraints. Which are even mentioned in the text (ln 613) but which are not explained in any part. We need to know more about the farmers organization and integration with the market. Which factors have influenced current socio-ecological trajectory? I understand those areas were hit by armed conflict and narcotrafic, how those facts have influenced in the cohesion of the cooperatives?.
****************************************
METHODS
The methods used are sophisticated and complex, I commend the authors for a well design and complete presentation of the methods section but still it is a little confusing, I found it difficult to follow and have to reread and look for the sections of which method or question was being answered, with the many options used. It will be good if you present a flowchart or diagram for the different methods and questions answered, for a better and faster understanding in a glance.

Do the native timber species are regularly harvested, with a cycling of shade trees?
Have you survey the shade trees? Density, size?
Explain better the difference of tree cover and forest cover? IT is confusing also that you have shade-tree
Pg 27 ln 497: Clarify what do you refer by true bugs, are you referring solely to Hemiptera? If yes, use that name

Pg 27 ln 297, so you extracted DNA from each specimen? How was this done?
Ln 531 Exclusion experiments: So you selected 4 trees for each agroforests? Why you talk about 2 pairs, do they were paired in some way? Do the exclosure cover both trees at the same time?
What was expected in the exclusion experiment? That pest effect were larger in the excluded trees but also higher pollination rates? Which are the main pollinators of Cacao flowers?
Ln 581: How do you compare between the exclusion and the control if you did not took data on the exclusion trees? What did you evaluated in the excluded trees? Fruit set or only final yield? Which data was included in Supplementary Table 3? How did you calculate Cacao yield? Was the commercial yield reported by the cooperatives?
Ln 620. Is it not 10 GLMM? 4 with each shade-tree structure variables x 2 landscape variable=8 models + 2 with the multidiversity
Ln 653: Supplementary Table 5. Is this table the correct one?
******************
RESULTS
Figure 1: Where do the numbers 40 & 80% landscape tree cover came. It is less than 40% and less than 80%? Need to explain in the Methods section
Fig 2: Why the diversity in the 40% is larger at 20% shade-tree cover. Can be an artifact?

Why the multidiversity decrease faster than the others in isolation.
Supplementary fig 4: Why cacao flowers production decrease with shade-tree cover but fruit set increase? Should not both be consistent? How are you calculating fruit set
Ln172 Instead saying “simple landscapes” would be better to say “low heterogeneity landscapes” or more homogeneous landscapes. There may be other unmeasured variables like forest composition or terrain, closeness to forest whxihc may be influencing.

***************************************

Minor corrections
Pg 28 ln 511: clarify this sentence: “...South American BOLD reference database curated by AIM… explaining the abbreviations. Maybe it is too obvious for the specialists but it is very confusing for the reader, try to explian in simpler terms and cite a relevant reference, or add a supplementary text with the options selected in the database.
Supplementary Figure 5. Can be interesting to distinguish between the two sites, use a different symbol for each site, seems lower sites have more acidic soils. Since each sampling site in a different basin can be high geological differences which may be influencing the soil characteristics
Supplementary Table 4 Check for typos, “poy?” “bird”

Reviewer #2

(Remarks to the Author)
Integrating local and landscape management promotes biodiversity, multifunctionality and yields in tropical agroforests

Aycart-Lazo study the biodiversity and ecosystem services and disservices in a Cacao agroforestry landscape in Western Amazonia. They show that tree cover and tree cover and height heterogeneity are strong drivers of biodiversity in this agroforestry system. Conversely, yield – biodiversity trade-offs were weak (and mostly non-significant). They showed that pest control by birds and bats showed a humped shaped relationship with tree cover and forest cover within 500 m, peaking at around 75% tree cover and 35% forest cover. Shade tree cover also had a positive effect on soil fertility and on reducing air temperature. The study is well conducted and the paper well written. The dataset is exceptional. What is particularly valuable is the characterization of services and disservices, and the role of habitat properties aside from just tree cover, including tree height and variance in tree cover and height. Their measurement of yields in bird and bat exclusion trees demonstrates the interacting roles of shade (and other disservices) and pest control services.

I have just a few comments:
1. Biodiversity in agroforests – including shade tree systems – is usually substantially lower than in natural forests (Gibson et al. 2011). The biodiversity measurements reported were only from agroforests, so it is difficult to assess the implications of the reported percentage increases in biodiversity. Moreover, not all species are equal, with agroforests tending to support disturbance tolerant species. Even if data are not available from forest locations, it would benefit the paper to discuss the role of these agroforestry habitats in harboring forest interior species and the implications for the conservation of biodiversity. It is perhaps a little surprising that the authors is not measure shade tree diversity (or plant diversity) in the plots, especially as diversity in other groups, such as invertebrates, is often strongly correlated to plant diversity.

2. We are not provided with a site map or any figures of the spatial distribution of habitat and biodiversity variables. In addition, the statistical analysis section describes only GLMMs and does not mention the inclusion of spatial variables. As land use decisions are not random, there is often strong covariance between landcover types. For example, a not unusual pattern is to find that agroforests become simplified as distance to natural forests increases. If this is the case, then variables such as shade tree cover and height might be strongly correlated with distance to forest, and if birds and bats are spilling over from natural forests they might less likely in these simpler systems as a consequence of distance effects. In short, the authors should explain how they tested for and / or controlled for spatial effects.

3. Shade agroforestry systems often suffer from a slow deforestation, as the natural shade trees die or are harvested. This means that older plots often have lower tree cover and less diverse tree cover. To maintain shade tree diversity and shade tree cover in the system requires deliberate planting and management of diverse shade tree species. Is this being practiced? How might it affect the long term maintenance of biodiversity in the system?

Rhett Harrison

Gibson, L., Lee, T. M., Koh, L. P., Brook, B. W., Gardner, T., Barlow, J., Peres, C. A., Bradshaw, C. J. A., Laurance, W. F., Lovejoy, T. E., & Sodhi, N. S. (2011). Primary forests are irreplaceable for sustaining tropical biodiversity. Nature, 478(7369), 378–381. https://doi.org/10.1038/nature10425

Reviewer #3

(Remarks to the Author)
Integrating local and landscape management promotes biodiversity, multifunctionality and yields in tropical agroforests
by Pablo Aycart-Lazo et al.

Where agroforestry systems represent a technological change, to be accepted by local people, at a minimum they have to be an economically favorable alternative. For them to meet the needs of stakeholders interested in sustainable land use that promotes biodiversity, they minimally must show biodiversity benefits as compared to agriculture without trees or limited tree

cover.

This study assessed multifunctionality of cacao agroforests in the northern Amazonian tropical forest at a dry-moist forest border. The authors assessed 28 agroforests split between two blocks 30 km distant. I greatly appreciate their inclusion of two distinct sites to avoid pseudo replication. The distance between agroforests is appropriate, as is their subsampling of agroforests with replicate trees per agroforest to sample for yield with and without exclosures. I have a few questions about and would like some clarifications with regards to some of the sampling for biodiversity. Overall, I find this study to be well done, the manuscript to be well written, and the conclusions that circle back to cacao yields timely and important. I enjoyed reading this manuscript, particularly how different management affects yields as determined by enclosures, fruit set, and disease prevalence.

Specific comments:
Lines 107-126, please see and address detailed comments on biodiversity inventories below. Please revise as appropriate.

Figures 1 and 2. As above

Line 116 and paragraph beyond. For several taxa assessed, one should be able to determine if the species are forest associated species and look at species composition across strata. One could not identify specific species for the work done by molecular analysis but should be for birds, bats, butterflies, and ants. For molecular analysis one would compare species composition by looking at community differences in OTUs. I would like to see or at least understand why community composition comparisons across land use strata (agroforest structures, land context, etc) were not made in addition to species richness/diversity, I think some discussion of this needs to be made or at least an acknowledgement of the limitation of the study.

Lines 186, 187. I presume you have inventory data for timber species. Can you estimate wood volumes and values?

Lines 234-237. This is an important point. A mechanistic understanding would certainly advance our understanding of tradeoffs. You have inventoried birds and bats (with methods sufficient to estimate insectivores) and should have data on community composition differences. I do not completely understand why these data are not included in the analysis and discussion. It would greatly enhance the manuscript.

Lines 253 and beyond. I really like this analysis and discussion.

Do lines 256 and 259 contradict each other with respect to yields?
Lines 291-292. I agree completely.

Lines 295 and 296. Please see my concerns about soil fertility sampling. I would like to see a justification for the limited sampling per agroforest. Please explain the mechanism by which the treatments could change soil potassium and provide a citation. Presumably it is included in citation 24.

The study suffers from the fact that no data are presented nor is there a description of soil classification. Without background information, how can one be sure that any differences that may be present are not due to background heterogeneity? I would like some reassurance.

As I look at the discussion of soil carbon in relation to fertility, I am struck by the fact that no analysis was made of aboveground carbon sequestration, a very important ecosystem service. Perhaps this could be included in a revision or at least discussed in reference to other studies. This is not a requirement for me. I see it is referenced in line 385. However, it is an important component of multifunctionality.

Line 426, can you give a citation for the stringent organic fair-trade standards?
Section starting with line 432, can you confirm there was no bias with percent slope and measured variables? Can slope affect yield, for example?

Line 456, Biodiversity surveys and metrics

Overall comments on biodiversity:
The study assessed seven taxonomic groups as indicators for comparisons of biodiversity. This section combines them and assesses them as an abundance-based comparison. I don't have any objection to the analysis pe se, but I have not seen a justification as to why these seven taxonomic groups were chosen. Many are the "usual suspects" in terms of biodiversity indicators, but others are perhaps not, at least for me (and this is not my area of expertise). I would like to see somewhere in the Methods and noted in the main text why these species were chosen and how they represent multifunctionality in terms of biodiversity. I understand, for example, that some bats and even some birds and butterflies are pollinators, but they do not pollinate cacao, and they are not responsible for the majority of pollination in tropical forest tree species (I understand bees are). I would like to see the choice of taxon surveyed justified. I acknowledge that this was a huge amount of work and is very impressive!

The manuscript focusses on species richness but not community composition. Raw numbers of species are not the best assessment for understanding the effects of land management on biodiversity from a conservation perspective. Rare species and, in this instance, forest dependent species would be of most interest. I would appreciate a discussion of this as

limitations of the study with respect to biodiversity as the analysis of species richness may be misleading with respect to understanding the conservation value of the different study units.

Line 460, bird surveys appear to have included sufficient replication. Can you provide a methods citation?

Line 467, bat surveys. Can you provide a citation for details on bioacoustics analyses. Are the data used in this manuscript the same data they published elsewhere? That can be fine, but you should be clear and note how the data are used differently here.

My understanding is that acoustic methods for sampling bats can do a good job of capturing the diversity of aerial insectivores but do not do a good job of differentiating between understory bat species. For this, mist nets are preferred. If this is true, then only one group of species was adequately sampled and that the sampling methods may not have adequately captured the bat diversity, particularly the understory species or the species that one might believe to be favored by more forest cover. Please discuss this in your sampling intensity in your response and adjust your manuscript as needed. I refer you to:

Meyer, C.F.J. et al. 2011. Accounting for detectability improves estimates of species richness in tropical bat surveys. Journal of Applied Ecology 48, 777–787.

Meyer, C.F.J. et al. 2010. Long-term monitoring of tropical bats for anthropogenic impact assessment: Gauging the statistical power to detect population change. Biological Conservation 143, 2797–2807.

Line 474, ant surveys. Can you provide a citation to justify sampling efforts and methods and also discuss limitations? My understanding is that the methods are appropriate for sampling arboreal ants but would not account for ground dwelling species. I admittedly do not know the expected percent difference in species but as for bats one might consider the species of ants found on the forest floor of interest in biodiversity surveys. Please discuss in response to reviewers and adjust manuscript as needed.

Line 384, butterfly surveys. It is interesting that you note that you assessed assemblages here. As I am sure you know, species composition is different from species richness, the metric reported in the manuscript. This overall observation of the biodiversity assessment was made above. With respect to butterflies, please provide a methods citation and discuss how the difference in these metrics affects your conclusions.

Line 493, Spider, true bug and beetle surveys. The sampling seems appropriate but, as with other surveys, a methods and sampling citation would be helpful and appreciated.

Line 511, species richness and multiversity calculation.
Please see overarching comments on biodiversity sampling above. I acknowledge that this was a huge amount of work and is very impressive!

Line 527 exclusion experiments and cacao yield evaluations
This is a huge and impressive amount of work. Very interesting.

Line, 566, my understanding is that cacao is pollinated by flies or midges. Is this true? This line suggests more generalist pollinators.

Line 580, agroforest soil fertility and microclimate
One sample per agroforest at 0-10 cm seems insufficient in terms of soil sampling. Typically, one would take multiple samples per agroforest, bulk them and then subsample if funds were limited (and one could not afford to analyze all samples individually). The sampling needs to be justified and perhaps these data should be excluded from the analysis as any given sample (or multiple single samples) could be idiosyncratic.

Line 611, statistical analyses
The statistical analyses appear to be well done to me but I defer to other reviewers with stronger analytical abilities. My concerns (which may be too strong of a word) are rather with the data that go into the analyses as noted above.

Version 1:

Reviewer comments:

Reviewer #1

(Remarks to the Author)
I am satified with the revisions. Congratulations! a nice paper.

Reviewer #2

(Remarks to the Author)
I believe the authors have addressed the reviewers' comments appropriately. I do not have any further comments.

**Open Access** This Peer Review File is licensed under a Creative Commons Attribution 4.0 International License, which permits use, sharing, adaptation, distribution and reproduction in any medium or format, as long as you give appropriate credit to the original author(s) and the source, provide a link to the Creative Commons license, and indicate if changes were made.
In cases where reviewers are anonymous, credit should be given to 'Anonymous Referee' and the source.
The images or other third party material in this Peer Review File are included in the article's Creative Commons license, unless indicated otherwise in a credit line to the material. If material is not included in the article's Creative Commons license and your intended use is not permitted by statutory regulation or exceeds the permitted use, you will need to obtain permission directly from the copyright holder.
To view a copy of this license, visit https://creativecommons.org/licenses/by/4.0/

Dear Dr. Soto-Caro,

We would like to thank you and the referees for your positive feedback and valuable comments on our manuscript "Integrating local and landscape management promotes biodiversity, multifunctionality and yields in tropical agroforests" (submission ID: COMMSENV-26-2598). We have answered all comments in our point-by-point response below (responses marked in blue) and adapted our manuscript accordingly. In addition, we now include in the data availability statement a link to the Figshare repository where we have uploaded the data used in the paper.

We believe these revisions have further strengthened our manuscript, and we are grateful for the opportunity to move it forward towards publication in *Communications Earth & Environment*.

Sincerely yours,

Pablo Aycart Lazo, on behalf of all coauthors.

**Reviewer #1 (Remarks to the Author):**

This is an interesting work where the authors comprehensively assess the ecosystem services in cacao agroforests. including biodiversity, pest and disease incidence protection, microclimate control, soil fertility and cacao yield. I found it very complete and engaging for a complex topic and a good integrative multi-scale design. I have some suggestions mainly to improve the readability of the paper.

We would like to sincerely thank the editor and reviewers for their constructive comments and thoughtful suggestions regarding our manuscript. The feedback provided was highly valuable in improving the overall clarity and quality of our work. Please find our responses to the specific comments below.

My main suggestion is to include some text, probably in supplementary material, about the social history of the 2 cooperatives in order to qualify claims of the type "Yield gains were not associated with species loss" which occur under current social and economic constraints. Which are even mentioned in the text (ln 613) but which are not explained in any part. We need to know more about the farmers organization and integration with the market. Which factors have influenced current socio-ecological trajectory? I understand those areas were hit by armed conflict and narcotrafic, how those facts have influenced in the cohesion of the cooperatives?.

We appreciate the suggestion regarding the social history of the two cooperatives and have included additional information on the socio-economic context of the San Martín department in section 3.1 (see below). However, we prefer not to elaborate further on the impacts of drug trafficking and armed conflicts on local cooperatives as this has limited relevance to our primary research questions, which do not address the impacts of land-use legacy but are limited to the effects of current shade-tree and landscape structure.

"*The San Martín department, responsible for 40% of the cacao production in Peru, has undergone a profound economic transformation over the past 30 years driven by alternative development policies*

*aimed at replacing illicit coca cultivation with high-value agroforestry crops such as cacao and coffee[8]. The expansion of robust, legal agricultural markets and the development of farmer associations have successfully integrated smallholders into global value chains, catalyzing regional infrastructure development, improved public safety, and substantial rural poverty reduction[80]*" (L 472-475).

Added references:

80. Cabieses, H. The 'Miracle of San Martín' and Symptoms of 'Alternative Development' in Peru. https://www.tni.org/en/publication/the-miracle-of-san-martin-and-symptoms-of-alternative-development-in-peru-0 (2010).

****************************************

METHODS

The methods used are sophisticated and complex, I commend the authors for a well design and complete presentation of the methods section but still it is a little confusing, I found it difficult to follow and have to reread and look for the sections of which method or question was being answered, with the many options used. It will be good if you present a flowchart or diagram for the different methods and questions answered, for a better and faster understanding in a glance.

Thank you for the suggestion. We have included a table (Table 1) summarizing the environmental predictors and response variables, the methods used to estimate them, and the research questions addressed in the study at the end of the introduction of the revised version. However, we leave it up to the editor to decide whether to move this table to the supplementary materials, if necessary.

Do the native timber species are regularly harvested, with a cycling of shade trees?

Both native and non-native timber trees are harvested and replaced by new ones in long-term cycles (15-30 years, depending on the shade-tree species). However, all cacao producers participating in the study agreed not to harvest shade trees during the data collection period. We now clarify this in Section 3.1: "*Although shade timber trees are regularly harvested and used as a secondary income source in our study area, agroforest owners committed to abstaining from harvesting them to maintain a consistent agroforest canopy structure throughout the study period*" (L 513-516).

Have you survey the shade trees? Density, size?

We surveyed shade-tree density, which was correlated with shade-tree cover (r = 0.62), and measured their DBH. However, we prefer to include only drone-derived canopy structure metrics in the study to maintain consistency across variables.

Explain better the difference of tree cover and forest cover? IT is confusing also that you have shade-tree

We now clarify the difference between shade-tree variables, landscape tree cover and landscape forest cover: "*Landscape complexity in the surroundings of the agroforest was estimated based on the forest and tree cover in 500-m buffers*[41]*, the latter including both forests and additional arboreal habitats. Therefore, 'shade-tree' variables refer to local, agroforest-level predictors (shade-tree cover, cover heterogeneity, height and height heterogeneity), landscape forest cover refers to the percentage of the 500-m buffers occupied by forested habitat, and landscape tree cover refers to the percentage of the buffers occupied by trees, including forested habitats, tree crops and other woody vegetation such as scattered trees*" (L 129-135).

Pg 27 ln 497: Clarify what do you refer by true bugs, are you referring solely to Hemiptera? If yes, use that name

Yes, we refer solely to Hemiptera. We have changed the term in the revised version to avoid confusion. Thank you for the suggestion!

Pg 27 ln 297, so you extracted DNA from each specimen? How was this done?

We did not extract DNA from each specimen individually, but from the bulk sample obtained via canopy fogging. We now provide additional information on the procedure to obtain and identify OTUs in these samples: "*Sample preparation (homogenization of bulk samples), laboratory work (DNA extraction from homogenized samples, amplification and sequencing) and bioinformatic analyses (OTU identification) were performed by Advanced Identification Methods GmbH (AIM; Leipzig, Germany; https://www.metabarcoding.com) following the protocols described in*[94] *and*[96]*. We used a geographically filtered South American Barcode of Life Data Systems (BOLD) reference database curated by AIM to identify our OTUs to the order level. This database was compiled by filtering the full public BOLD dataset (downloaded on July 4, 2025) to retain valid barcode records restricted to South American countries and geographic coordinates between -58° and 17° latitude, and -86° and -30° longitude. Records from Costa Rica were additionally integrated into the reference database due to its high density of barcode sequences within the BOLD repository. We compared OTUs identified in the samples with this geo-delimited dataset and kept those with a BOLD Grade ID of at least 85%, the minimum required to identify OTUs at the order level*[94]*. We then selected OTUs belonging to the orders Araneae (spiders), Hemiptera (true bugs) and Coleoptera (beetles) and summed the number of reads per OTU and agroforest*" (L 619-632).

Ln 531 Exclusion experiments: So you selected 4 trees for each agroforests? Why you talk about 2 pairs, do they were paired in some way? Do the exclosure cover both trees at the same time?

Yes, we built two exclosures per agroforest, each of them covering two contiguous cacao trees. We now clarify this: "*In each agroforest, we selected two pairs of contiguous cacao trees and built one exclosure for each pair. Therefore, each of the two exclosures covered two trees at the same time*" (L 652-654).

What was expected in the exclusion experiment? That pest effect were larger in the excluded trees but also higher pollination rates? Which are the main pollinators of Cacao flowers?

We expected lower cacao yield in the exclusion trees due to higher pest incidence and damage by phytophagous arthropods: "*By excluding bird and bat insectivores, we expected an increase in phytophagous arthropod damage to cacao leaves, flowers and developing fruits in the exclusion trees, ultimately resulting in reduced harvestable yields compared to control trees*[40]" (L 659-661).

Cacao pollination is a complex, not yet fully understood process in which the identity of the main pollinators remains unclear, although midges of the family Ceratopogonidae are usually suggested to be important pollinators. In our study, we aimed to identify local and landscape management practices potentially enhancing cacao pollination under natural conditions, not under bird and bat exclusion. Therefore, we measured cacao fruit-set (as a proxy of pollination rates) only in control trees, as analyzing the cascading effects of bird and bat exclusion, and their interactions with shade-tree canopy management and landscape context, on pollination rates was beyond the scope of this study. We now clarify this in section 3.5: "*Cacao pollination is a complex process involving intricate ecological interactions in which, despite the presumed importance of ceratopogonid midges, the precise identity of the primary pollinators remains a subject of ongoing debate*[100,101]*. Rather than analyzing the cascading effects of arthropod suppression by birds and bats on cacao pollination, we aimed to identify shade-tree and landscape management strategies promoting pollination under natural conditions. Therefore, we assessed pollination services in the agroforests based on the fruit-set of cacao control trees only. As cacao flowers cannot self-pollinate, cacao fruit-set is a good proxy for pollination services in agroforests*[14]" (L 690-696).

Added references:

100. Van de Sande, E. et al. Revisiting cacao pollination: new behavioural and pollen-based evidence for the key role of Ceratopogonidae. *Basic Appl. Ecol*. 94, 24–33 (2026).

101. Toledo-Hernández, M., Wanger, T. C. & Tscharntke, T. Neglected pollinators: Can enhanced pollination services improve cocoa yields? A review. *Agric. Ecosyst. Environ*. 247, 137–148 (2017).

Ln 581: How do you compare between the exclusion and the control if you did not took data on the exclusion trees? What did you evaluated in the excluded trees? Fruit set or only final yield? Which data was included in Supplementary Table 3? How did you calculate Cacao yield? Was the commercial yield reported by the cooperatives?

We appreciate the opportunity to clarify these aspects regarding our exclusion experiment and yield evaluations.

As noted in our response to the previous comment, we did not compare cacao fruit-set between exclusion and control treatments. Instead, we analyzed the effects of local shade-tree management and landscape tree and forest cover on fruit-set in control trees only. Within the exclusion trees, our evaluation was limited to annual cacao yield. We compared these values against control yields to calculate

the yield difference, which serves as our proxy for the economic contribution of bird- and bat-mediated pest control.

Supplementary Table 3 presents the parameter estimates of the best-fitting GLMMs analyzing the influence of shade-tree and landscape metrics on annual cacao yield across both treatments. Consequently, this model integrates data from all 128 monitored cacao trees (64 control trees and 64 exclusion trees). To clarify this, we have updated the caption of the Supplementary Table 3 as follows: "*Supplementary Table 3. Results of the best-fitting GLMMs analyzing the effects of shade-tree variables and landscape tree and forest cover on the annual yield (kg of dry cacao beans) of control and exclusion trees*".

Finally, to ensure standardization, annual cacao yields were calculated at the individual tree level. We monitored pod production across all 128 trees over a full year (September 2022 to September 2023) and multiplied this by the average dry bean mass per pod (detailed in Section 3.4, paragraphs 2 and 3). We could not use the cacao cooperative records for this analysis because cooperative databases only register the aggregate wet bean mass delivered by each farmer. Because the number of farms managed by each member of the cooperative, as well as their sizes, varies widely among farmers, cooperative records are not standardized and therefore cannot be used to model local and landscape-level effects on cacao yield.

Ln 620. Is it not 10 GLMM? 4 with each shade-tree structure variables x 2 landscape variable=8 models + 2 with the multidiversity

Multidiversity was used as a response variable in our analyses. Therefore, we had 8 response variables (bird, bat, ant, butterfly, spider, Hemiptera and beetle richness + multidiversity) and fitted 8 models (4 with each shade-tree structure variables x 2 landscape variable) for each of them. We now explicitly mention that we fitted eight models for each of the eight response variables to clarify this: "*To assess biodiversity responses to shade-tree and landscape variables, we fitted eight generalized linear mixed models (GLMMs) for each of the eight response variables using Gamma distributions and logarithmic link functions for the seven richness variables, and beta distributions with logit link functions for multidiversity*" (L 745-748).

Ln 653: Supplementary Table 5. Is this table the correct one?

The correct table is Supplementary Table 3. Sorry for the confusion, and thanks for noticing!

We have corrected the reference in the revised version of the manuscript and clarified that we refer to the best-fitting models analyzing shade-tree and landscape effects on cacao yield: "*As none of the best-fitting yield models for cacao yield included interactions between shade-tree predictors and treatment (Supplementary Table 3), we only included landscape predictors in the Linear Mixed Models (LMMs) analyzing bird and bat contributions to cacao yield (agroforest-level Δyield)*" (L 783-785).

******************

RESULTS

Figure 1: Where do the numbers 40 & 80% landscape tree cover came. It is less than 40% and less than 80%? Need to explain in the Methods section

We now clarify in section 3.7 that these values correspond to the average tree cover minus/plus one standard deviation: "*In case of significant interactions between local shade-tree and landscape predictors in the best-fitting models, we projected the model to assess the influence of the shade-tree predictor on the response variable in landscapes with, on average, low tree or forest cover (40% tree cover or 6% forest cover; mean tree and forest cover minus one standard deviation) and in landscapes with high tree or forest cover (80% tree cover or 37% forest cover, mean tree and forest cover plus one standard deviation)*" (L 758-763).

Fig 2: Why the diversity in the 40% is larger at 20% shade-tree cover. Can be an artifact?

Rather than being the result of an artifact, we argue that the contrasting responses of birds and ants to shade-tree cover at different levels of landscape tree cover reflect a replacement of open-area generalists by forest-associated species towards landscapes with higher tree cover: "*In simplified landscapes with relatively low tree cover (≈40%; mean tree cover [60%] - one sd [20%]), bird and ant richness declined by 72% and 90%, respectively, as shade-tree cover increased from 10% to 80% (Fig. 2A-B). In contrast, in complex, tree-rich landscapes (≈80% tree cover; mean + one sd), bird richness increased by 180%, and ant richness by 495% along the same gradient. These divergent responses along the shade tree cover gradient likely display the different community compositions in agroforests located in landscapes with low or high tree cover. Open area generalists, which we expect to be more dominant in landscapes with low tree cover, are likely favored by low-shade management, while forest-associated species whose presence relies on a high tree cover in the landscape require dense shade-tree cover*[42,45]" (L 143-151).

Why the multidiversity decrease faster than the others in isolation.

We now provide a potential explanation for the observed decrease in agroforest multidiversity: "*Vertically continuous shade-tree canopies may preserve microclimatic stability within the understory, facilitating arthropod persistence and fostering multidiversity in simplified landscapes exposed to elevated temperatures and wind*[47,48]" (L 209-211).

Added references:

48. Brandon-Mong, G.-J. et al. Temporal changes in arthropod activity in tropical anthropogenic forests. *Bull. Entomol. Res*. 108, 792–799 (2018).

Supplementary fig 4: Why cacao flowers production decrease with shade-tree cover but fruit set increase? Should not both be consistent? How are you calculating fruit set

Cacao fruit-set was quantified as the proportion of cacao flowers that developed fruits. Therefore, fruit-set and flower production can follow opposite trends if the amount of flowers produced decreases, but there is a greater proportion of these flowers that end up developing fruits. We now explicitly inform the readers about the calculation of cacao fruit-set in the main text: "*Cacao fruit-set—quantified as the proportion of flowers that develop fruits and used as a proxy of the effectiveness of pollination services in agroforests*[14]*—was unaffected by landscape complexity but increased from 1.6% at 15% shade-tree cover to 6.4% at 80% (Fig. 4C), probably because of higher flower visitor abundance*[22]" (L 303-306).

Ln172 Instead saying "simple landscapes" would be better to say "low heterogeneity landscapes" or more homogeneous landscapes. There may be other unmeasured variables like forest composition or terrain, closeness to forest whxihc may be influencing.

Thank you for the suggestion. To be more specific, we have changed the reference to 'simple landscapes' to 'landscapes with only 40% tree cover': "*Increasing shade-tree cover heterogeneity resulted in a 172% increase in bird richness and a 632% increase in ant richness in complex landscapes with 80% tree cover, but contrary to shade-tree cover, it did not affect species richness in landscapes with only 40% tree cover (Fig. 2C-D) nor cacao yields (Fig. 2G-I)*" (L 199-202).

***************************************

Minor corrections

Pg 28 ln 511: clarify this sentence: "...South American BOLD reference database curated by AIM… explaining the abbreviations. Maybe it is too obvious for the specialists but it is very confusing for the reader, try to explian in simpler terms and cite a relevant reference, or add a supplementary text with the options selected in the database.

Thank you for the suggestion. We have adapted the paragraph in the revised version to clarify the abbreviations and include additional information on the procedure followed to create the reference database used in the bioinformatic analyses (L 604-618; see our response to the comment on Pg 27 ln 297 above).

Supplementary Figure 5. Can be interesting to distinguish between the two sites, use a different symbol for each site, seems lower sites have more acidic soils. Since each sampling site in a different basin can be high geological differences which may be influencing the soil characteristics

Many thanks for the suggestion. We now use squares and triangles to differentiate between agroforests located in different valleys in the figures representing shade-tree structure and elevation effects on soil composition (Figure 5A-D and Supplementary Figure 4).

Supplementary Table 4 Check for typos, "poy?" "bird"

Corrected. Thank you for noticing!

**Reviewer #2 (Remarks to the Author):**

Integrating local and landscape management promotes biodiversity, multifunctionality and yields in tropical agroforests

Aycart-Lazo study the biodiversity and ecosystem services and disservices in a Cacao agroforestry landscape in Western Amazonia. They show that tree cover and tree cover and height heterogeneity are strong drivers of biodiversity in this agroforestry system. Conversely, yield – biodiversity trade-offs were weak (and mostly non-significant). They showed that pest control by birds and bats showed a humped shaped relationship with tree cover and forest cover within 500 m, peaking at around 75% tree cover and 35% forest cover. Shade tree cover also had a positive effect on soil fertility and on reducing air temperature. The study is well conducted and the paper well written. The dataset is exceptional. What is particularly valuable is the characterization of services and disservices, and the role of habitat properties aside from just tree cover, including tree height and variance in tree cover and height. Their measurement of yields in bird and bat exclusion trees demonstrates the interacting roles of shade (and other disservices) and pest control services.

We thank the reviewers for their time, positive feedback, and insightful recommendations. Please find our response to the specific comments below.

I have just a few comments:

1. Biodiversity in agroforests – including shade tree systems – is usually substantially lower than in natural forests (Gibson et al. 2011). The biodiversity measurements reported were only from agroforests, so it is difficult to assess the implications of the reported percentage increases in biodiversity. Moreover, not all species are equal, with agroforests tending to support disturbance tolerant species. Even if data are not available from forest locations, it would benefit the paper to discuss the role of these agroforestry habitats in harboring forest interior species and the implications for the conservation of biodiversity. It is perhaps a little surprising that the authors is not measure shade tree diversity (or plant diversity) in the plots, especially as diversity in other groups, such as invertebrates, is often strongly correlated to plant diversity.

Thank you for bringing attention to this important point. We now discuss these implications of agroforestry for the conservation of forest-associated species and agroforest multifunctionality in sections 2.1, 2.2 and 2.4:

"*In simplified landscapes with relatively low tree cover (≈40%; mean tree cover [60%] - one sd [20%]), bird and ant richness declined by 72% and 90%, respectively, as shade-tree cover increased from 10% to 80% (Fig. 2A-B). In contrast, in complex, tree-rich landscapes (≈80% tree cover; mean + one sd), bird richness increased by 180%, and ant richness by 495% along the same gradient. These divergent responses along the shade-tree cover gradient likely display the different community compositions in agroforests located in landscapes with low or high tree cover. Open area generalists, which we expect to be more dominant in*

*landscapes with low tree cover, are likely favored by low-shade management, while forest-associated species whose presence relies on a high tree cover in the landscape require dense shade-tree cover*[42,45]" (L 143-151).

"*While tropical agroforests can sustain faunal species richness comparable to adjacent forests, their conservation value is also determined by their capacity to preserve forest-associated species. Previous studies have shown that agroforests typically harbor simplified species assemblages characterized by a decrease in forest specialists and a dominance of generalist species, with implications for shade-tree and landscape management*[42,51]*. For instance, lower shade-tree cover was related to a decrease in the species richness of forest-associated ants and butterflies in Indonesian and Peruvian agroforests, respectively*[42,44]*. Additionally, increasing distance to the forest and local management intensity are usually associated with lower richness and abundance of forest birds in cacao agroforests*[52,53]*. Regarding bats, the sole use of ultrasonic recorders to estimate their species richness prevented us from analyzing shade-tree and landscape management effects on phyllostomid bats (family Phyllostomidae)*[54]*, many of which are understory and forest-dependent species that do not thrive in simplified landscapes*[55]*. Consequently, agroforests must serve as a complement to, rather than a replacement for, remnant forests within tropical agricultural landscapes*[56]*.*" (L 224-236).

"*Higher forest cover may also facilitate the spillover of gleaning insectivorous birds and bats—many of which are forest-associated species*[58,59]*—into agroforests, ultimately enhancing arthropod suppression*[60]*. Indeed, previous studies in our study area demonstrate a progressive increase in the dominance of forest-associated insectivorous birds towards forested landscapes and lower feeding activity of aerial insectivorous bats with increasing prevalence of monocultures in the landscape*[45,61]" (L 268-273).

Regarding shade-tree diversity effects, we could include only a limited number of parameters in our models to avoid overfitting them. While shade-tree diversification is known to have positive effects on agroforest biodiversity, we prioritized including understudied variables related to shade-tree structure (e.g., cover and height heterogeneity) in our models. However, we now discuss some potential benefits of promoting shade-tree diversity:

"*Targeted replanting also offers opportunities for shade-tree diversification, which could help mitigate phytophagous insect damage through resource dilution while simultaneously promoting pest control and biodiversity conservation in agroforests by benefiting forest-associated insectivorous birds and bats*[45,51,75]" (L 423-426).

Added references:

51. Bennett, R. E., Sillett, T. S., Rice, R. A. & Marra, P. P. Impact of cocoa agricultural intensification on bird diversity and community composition. *Conserv. Biol*. 36, e13779 (2022).

52. Oliveira, I. S., Bandeira, E. C., Figueiredo, M. G. & Morante-Filho, J. C. Local management intensity and landscape forest cover affect bird community structure in shaded cocoa agroforests. *Ornithol. Res.* 34, 13 (2026).

53. Clough, Y., Dwi Putra, D., Pitopang, R. & Tscharntke, T. Local and landscape factors determine functional bird diversity in Indonesian cacao agroforestry. *Biol. Conserv.* 142, 1032–1041 (2009).

54. Yoh, N., Syme, P., Rocha, R., Meyer, C. F. J. & López-Baucells, A. Echolocation of Central Amazonian 'whispering' phyllostomid bats: call design and interspecific variation. *Mammal Res*. 65, 583–597 (2020).

55. Klingbeil, B. T. & Willig, M. R. Guild-specific responses of bats to landscape composition and configuration in fragmented Amazonian rainforest. J. Appl. Ecol. 46, 203–213 (2009).

56. Gibson, L. et al. Primary forests are irreplaceable for sustaining tropical biodiversity. *Nature* 478, 378–381 (2011).

61. Aycart-Lazo, P. et al. Tree cover loss and intensified land use simplify bat assemblages in Amazonian cacao agroforestry landscapes. *Landsc. Ecol*. 41, 115 (2026).

75. Ivañez-Ballesteros, B. et al. Local canopy cover, shade tree identity and landscape tree cover shape leaf-cutter ant nest density and herbivory in cacao agroforestry systems. *J. Appl. Ecol*. 63, e70359 (2026).

2. We are not provided with a site map or any figures of the spatial distribution of habitat and biodiversity variables. In addition, the statistical analysis section describes only GLMMs and does not mention the inclusion of spatial variables. As land use decisions are not random, there is often strong covariance between landcover types. For example, a not unusual pattern is to find that agroforests become simplified as distance to natural forests increases. If this is the case, then variables such as shade tree cover and height might be strongly correlated with distance to forest, and if birds and bats are spilling over from natural forests they might less likely in these simpler systems as a consequence of distance effects. In short, the authors should explain how they tested for and / or controlled for spatial effects.

We apologize if this was not sufficiently clear in the original version of the manuscript. To address the reviewer's point regarding the spatial context of the studied agroforests, we have moved the site map previously included in the Supplementary Figure 1 to the main text (Figure 7).

To clarify the confusion regarding the inclusion of spatial variables in the statistical analyses, we now specify that the landscape variables included in the GLMMs (tree and forest cover) were measured in 500 m buffers around the agroforests: "*Four of the eight models included one of the shade-tree structure variables (shade-tree cover, cover heterogeneity, height or height heterogeneity) and their interaction with landscape tree cover, measured in the 500 m buffers around the agroforests. The remaining four models included each shade-tree variable and its interaction with landscape forest cover in the same buffers*" (L 748-752).

Additionally, while the reviewer's concerns about the potential correlation between local management variables such as shade-tree cover and distance to forest are valid, these correlations were not observed in the studied agroforests. Although we did not include distance to forest as one of our landscape predictors, the correlations between shade-tree cover and landscape tree and forest cover were always low ($|r| < 0.2$; Supplementary Table 9). The only correlation we found between local and landscape variables was between shade-tree height and landscape forest cover in the agroforests included in the yield assessments ($r = 0.61$). In this case, we removed shade-tree height from the GLMMs to ensure all variables had VIF values below 6 (Supplementary Table 11).

Finally, we used Moran's I test to assess the spatial autocorrelation of the residuals of all GLMMs and found no signs of spatial bias in the performance of the models ($p > 0.1$; Supplementary Table 12).

3. Shade agroforestry systems often suffer from a slow deforestation, as the natural shade trees die or are harvested. This means that older plots often have lower tree cover and less diverse tree cover. To maintain shade tree diversity and shade tree cover in the system requires deliberate planting and management of diverse shade tree species. Is this being practiced? How might it affect the long term maintenance of biodiversity in the system?

This is an important, and sometimes overlooked, point about agroforest management. Despite being promoted by cacao cooperatives, active shade-tree replanting in the studied agroforests was not equally practiced, but varied depending on farmers' long-term perceptions and plans. We now highlight the importance of active shade-tree replanting to maintain agroforest long-term sustainability in section 2.4: "*Preserving the ecological and economic benefits of heterogeneous agroforest canopies over the long term requires active shade-tree replanting to offset losses from timber harvesting and natural mortality. Targeted replanting also offers opportunities for shade-tree diversification, which could help mitigate phytophagous insect damage through resource dilution while simultaneously promoting pest control and biodiversity conservation in agroforests by benefiting forest-associated insectivorous birds and bats*[45,51,75]*. Decision-support tools, such as diversityforagroforestry.org and cacaodiversity.org*[76]*, enable farmers and practitioners to identify optimal shade-tree species and cover levels tailored to specific management goals and local environmental conditions, facilitating shade-tree renewal and diversification*" (L 421-429).

**Reviewer #3 (Remarks to the Author):**

Integrating local and landscape management promotes biodiversity, multifunctionality and yields in tropical agroforests

by Pablo Aycart-Lazo et al.

Where agroforestry systems represent a technological change, to be accepted by local people, at a minimum they have to be an economically favorable alternative. For them to meet the needs of stakeholders interested in sustainable land use that promotes biodiversity, they minimally must show biodiversity benefits as compared to agriculture without trees or limited tree cover.

This study assessed multifunctionality of cacao agroforests in the northern Amazonian tropical forest at a dry-moist forest border. The authors assessed 28 agroforests split between two blocks 30 km distant. I greatly appreciate their inclusion of two distinct sites to avoid pseudo replication. The distance between agroforests is appropriate, as is their subsampling of agroforests with replicate trees per agroforest to sample for yield with and without exclosures. I have a few questions about and would like some

clarifications with regards to some of the sampling for biodiversity. Overall, I find this study to be well done, the manuscript to be well written, and the conclusions that circle back to cacao yields timely and important. I enjoyed reading this manuscript, particularly how different management affects yields as determined by enclosures, fruit set, and disease prevalence.

We appreciate the reviewer´s thoughtful comments and suggestions and have addressed them carefully and to the best of our possibilities. Below, we respond to the reviewer´s specific comments and indicate how they were implemented in the revised version of the manuscript.

Specific comments:

Lines 107-126, please see and address detailed comments on biodiversity inventories below. Please revise as appropriate.

Figures 1 and 2. As above

Please find our responses to the specific comments below.

Line 116 and paragraph beyond. For several taxa assessed, one should be able to determine if the species are forest associated species and look at species composition across strata. One could not identify specific species for the work done by molecular analysis but should be for birds, bats, butterflies, and ants. For molecular analysis one would compare species composition by looking at community differences in OTUs. I would like to see or at least understand why community composition comparisons across land use strata (agroforest structures, land context, etc) were not made in addition to species richness/diversity, I think some discussion of this needs to be made or at least an acknowledgement of the limitation of the study.

Thank you for bringing attention to this important topic. We agree that analyzing local and landscape management effects on the composition of species assemblages is highly relevant. However, we believe that introducing these additional multivariate ordinations and guild-specific GLMMs would disproportionately shift the manuscript's focus toward a detailed biodiversity assessment and may overcomplicate the paper, potentially distracting the reader from the key points of the integrated assessment. Therefore, to maintain the intended balance of the study, in which we try to give equal importance to the biodiversity component and the additional evaluations of agroforest multifunctionality and yield, we prefer to keep the focus on our primary metrics of species richness.

However, we acknowledge the relevance of changes in assemblage composition in the revised version and discuss potential implications of our results for forest-associated species based on previous findings (please see our response to point 1 of Reviewer #2 above).

Lines 186, 187. I presume you have inventory data for timber species. Can you estimate wood volumes and values?

We identified shade-tree species with the help of the local farmers and agroforest owners. Although we identified most shade trees to the species level, we could not associate the local common name with a

specific species in some cases. In addition, we did not measure the height of each individual tree, but calculated agroforest shade-tree height using drone images. Therefore, we were not able to estimate wood volumes and aboveground carbon values.

Lines 234-237. This is an important point. A mechanistic understanding would certainly advance our understanding of tradeoffs. You have inventoried birds and bats (with methods sufficient to estimate insectivores) and should have data on community composition differences. I do not completely understand why these data are not included in the analysis and discussion. It would greatly enhance the manuscript.

As mentioned in our response to the comment on Line 116 and the paragraph beyond, we would prefer to stick to our primary metrics of species richness to avoid shifting the main focus of the study towards its biodiversity component. Rather than including additional guild-specific analyses, we now discuss these results based on previous findings in our study area: "*Indeed, previous studies in our study area demonstrate a progressive increase in the dominance of forest-associated insectivorous birds towards forested landscapes and lower feeding activity of aerial insectivorous bats with increasing prevalence of monocultures in the landscape*[45,61]" (L 270-273).

Lines 253 and beyond. I really like this analysis and discussion.

Thank you very much!

Do lines 256 and 259 contradict each other with respect to yields?

Not necessarily, as our yield measurement is based on the total number of mature cacao pods harvested from the monitored cacao trees during a complete year. We argue that this discrepancy between the reduction in the total number of cacao flowers developing fruits (-1.6%) and the final yield (-76%) along the shade-tree cover gradient is likely explained by fruit abortion at later stages of pod development: "*Therefore, the observed 76% yield decline with increasing shade-tree cover was most likely driven by light competition leading to fruit abortion during pod development*[4]*, as shaded conditions did not increase pest or disease incidence and had no negative effects on soil fertility and moisture (Fig. 1)*" (L 311-314).

Lines 291-292. I agree completely.

Thanks!

Lines 295 and 296. Please see my concerns about soil fertility sampling. I would like to see a justification for the limited sampling per agroforest. Please explain the mechanism by which the treatments could change soil potassium and provide a citation. Presumably it is included in citation 24.

We appreciate the reviewer's point regarding the limitations of our soil assessments. We agree that a single sample per agroforest does not fully represent farm-scale trends and acknowledge this limitation in our discussion: "*However, as localized shade-tree effects on soil composition are not necessarily translated into agroforest-level fertility gains[70], these findings should be interpreted with caution*" (L 361-363).

Despite this limitation, we believe the soil data are valuable because we specifically paired each soil measurement with the canopy structure quantified directly above that exact sampling point (as explained in section 3.6, paragraph 4). Bulking different samples before the soil composition analyses would have homogenized these small-scale variations, limiting our ability to test for localized effects of shade-tree structure on soil composition. We now justify this decision regarding the soil sampling in section 3.6: "*Soil fertility was assessed based on the pH and potassium, phosphorus, total nitrogen and organic carbon contents of soil samples extracted at 0-10 cm depth near the center of the agroforest (one sample per agroforest). To evaluate the localized influence of shade-tree canopy structure on soil fertility, we collected a single soil sample per agroforest rather than a homogenized composite sample. This ensured the soil data remained paired with the canopy metrics quantified directly overhead*" (L 611-715).

Additionally, we now explain how higher shade-tree cover may increase soil potassium through organic matter decomposition and leaching from live shade-tree leaves: "*At higher shade-tree covers, there is enhanced input of nutrients from shade-tree leaf litter decomposition and potentially greater leaching of potassium from live leaf tissue of the shade trees, which may explain the increased potassium levels under closed-canopy conditions[68,69]. However, increases in shade-tree cover are paired with a decrease in heterogeneity as a result of more uniform canopies (Supplementary Figure 2), potentially explaining the negative effects of shade-tree cover heterogeneity on soil potassium contents (Fig. 5B)*" (L 352-357).

Added references:

69. Wood, T. E., Lawrence, D. & Clark, D. A. Variation in leaf litter nutrients of a Costa Rican rain forest is related to precipitation. *Biogeochemistry* 73, 417–437 (2005).

The study suffers from the fact that no data are presented nor is there a description of soil classification. Without background information, how can one be sure that any differences that may be present are not due to background heterogeneity? I would like some reassurance.

We regret that our dataset was not accessible at the start of the review process. To ensure full transparency and reproducibility, all raw data underlying our analyses, including the soil chemistry measurements, are now publicly available in the following Figshare repository: https://doi.org/10.6084/m9.figshare.33173981.

Additionally, we now include the soil classification in the description of the study area: "*Soils in both regions are classified as Orthic Acrisols, characterized by high acidity and low nutrient retention capacity[81]*" (L 489-490).

Added reference:

81. Food and Agriculture Organization of the United Nations (FAO). FAO-UNESCO Soil Map of the World: Revised legend with corrections and updates. (1997).

As I look at the discussion of soil carbon in relation to fertility, I am struck by the fact that no analysis was made of aboveground carbon sequestration, a very important ecosystem service. Perhaps this could be included in a revision or at least discussed in reference to other studies. This is not a requirement for me. I see it is referenced in line 385. However, it is an important component of multifunctionality.

Thank you for the suggestion. We agree that aboveground carbon storage is an important component of agroforest multifunctionality. Unfortunately, our drone-based assessments of shade-tree canopy structure prevented us from estimating the heights of specific shade trees. Therefore, we could not link wood density and shade-tree height values, a requisite for calculating the aboveground biomass of shade trees. However, we now discuss the implications of increasing shade-tree cover for aboveground carbon sequestration based on the findings of Blaser et al. (2018): "*Although we did not directly quantify carbon sequestration, previous studies have reported strong positive correlations with shade-tree cover in cacao agroforests[4]. Based on their results, maintaining 30% shade-tree cover would increase aboveground carbon by ≈200 t ha$^{-1}$ relative to unshaded monocultures, further enhancing agroforest multifunctionality[3]*" (L 416-420).

4. Blaser, W. J. et al. Climate-smart sustainable agriculture in low-to-intermediate shade agroforests. *Nat. Sustain*. 1, 234–239 (2018).

Line 426, can you give a citation for the stringent organic fair-trade standards?

Included: "*83. Fairtrade International. Fairtrade Standard for Cocoa v.2.14. (2023)*".

Section starting with line 432, can you confirm there was no bias with percent slope and measured variables? Can slope affect yield, for example?

To minimize potential slope effects and facilitate the different assessments, we prioritized flat agroforests during site selection. For those agroforests that still had some slopes, we corrected for slope when constructing the digital elevation models used to quantify shade-tree structural variables. Furthermore, all trees selected for yield monitoring were situated in flat areas of the agroforest to facilitate exclosure installation and maintain similar conditions between exclusion and control trees. Therefore, we are confident that topography did not influence our yield evaluations.

We now specify that the digital elevation models were corrected to avoid slope influence in the characterization of shade-tree structure: "*DEMs were corrected to account for agroforest slopes before calculating shade-tree variables*" (L 527-528).

Line 456, Biodiversity surveys and metrics

Overall comments on biodiversity:

The study assessed seven taxonomic groups as indicators for comparisons of biodiversity. This section combines them and assesses them as an abundance-based comparison. I don't have any objection to the analysis pe se, but I have not seen a justification as to why these seven taxonomic groups were chosen. Many are the "usual suspects" in terms of biodiversity indicators, but others are perhaps not, at least for me (and this is not my area of expertise). I would like to see somewhere in the Methods and noted in the main text why these species were chosen and how they represent multifunctionality in terms of biodiversity. I understand, for example, that some bats and even some birds and butterflies are pollinators, but they do not pollinate cacao, and they are not responsible for the majority of pollination in tropical forest tree species (I understand bees are). I would like to see the choice of taxon surveyed justified. I acknowledge that this was a huge amount of work and is very impressive!

Thank you for the suggestion. We now justify the selection of the taxa assessed at the beginning of section 3.3: "*In each of the 28 cacao agroforests, we assessed the diversity of two vertebrate and five arthropod taxa, namely: birds, aerial insectivorous bats, ants, butterflies, spiders, true bugs (order Hemiptera), and beetles. As highly mobile organisms, insectivorous birds and bats act as critical top-down regulators of arthropod populations, while frugivorous and nectarivorous species utilize agroforests as transient or complementary habitats, promoting forest regeneration through seed dispersal and pollination*[37,59,84]*. Additionally, arthropods such as ants and spiders, as well as predatory beetle and Hemiptera species, form a complex mesopredator network that may further regulate herbivore dynamics*[38,43,85]*. Conversely, specialized herbivores such as butterfly caterpillars, sap-sucking Hemiptera and leaf-cutter ants represent lower-trophic guilds that can damage cacao tissues, flowers and developing pods, potentially limiting agroforest productivity*[39,75]*. Therefore, the selected taxa serve as complementary ecological indicators for assessing how shade-tree configuration and landscape context shape agroforest biodiversity and multifunctionality*" (L 544-555).

Additionally, we mention in the main text that the selected taxa serve as valuable ecological indicators of agroforest multifunctionality: "*By participating in multiple ecosystem services and disservices, such as biological pest control and crop damage, the selected taxa serve as valuable ecological indicators of agroforest multifunctionality*[37–39]" (L 119-121).

Added references:

37. Maas, B. et al. Bird and bat predation services in tropical forests and agroforestry landscapes. *Biol. Rev.* 91, 1081–1101 (2016).

38. Philpott, S. M. & Armbrecht, I. Biodiversity in tropical agroforests and the ecological role of ants and ant diversity in predatory function. *Ecol. Entomol.* 31, 369–377 (2006).

84. Muscarella, R. & Fleming, T. H. The Role of Frugivorous Bats in Tropical Forest Succession. *Biol. Rev.* 82, 573–590 (2007).

85. Ocampo-Ariza, C. et al. Birds and bats enhance cacao yield despite suppressing arthropod mesopredation. *Ecol. Appl.* 33, e2886 (2023).

[1]The manuscript focusses on species richness but not community composition. Raw numbers of species are not the best assessment for understanding the effects of land management on biodiversity from a conservation perspective. Rare species and, in this instance, forest dependent species would be of most interest. I would appreciate a discussion of this as limitations of the study with respect to biodiversity as the analysis of species richness may be misleading with respect to understanding the conservation value of the different study units.

We now discuss the implications of agroforestry and landscape management for the conservation of forest-associated species in agricultural landscapes: "*While tropical agroforests can sustain faunal species richness comparable to adjacent forests, their conservation value is also determined by their capacity to preserve forest-associated species. Previous studies have shown that agroforests typically harbor simplified species assemblages characterized by a decrease in forest specialists and a dominance of generalist species, with implications for shade-tree and landscape management[42,51]. For instance, lower shade-tree cover was related to a decrease in the species richness of forest-associated ants and butterflies in Indonesian and Peruvian agroforests, respectively[42,44]. Additionally, increasing distance to the forest and local management intensity are usually associated with lower richness and abundance of forest birds in cacao agroforests[52,53]. Regarding bats, the sole use of ultrasonic recorders to estimate their species richness prevented us from analyzing shade-tree and landscape management effects on phyllostomid (family Phyllostomidae)[54], many of which are understory and forest-dependent species that do not thrive in simplified landscapes[55]. Consequently, agroforests must serve as a complement to, rather than a replacement for, remnant forests within tropical agricultural landscapes[56].*" (L 224-236).

Line 460, bird surveys appear to have included sufficient replication. Can you provide a methods citation?

We now provide the following citation:

86. Ralph, C. J., Geupel, G. R., Pyle, P., Martin, T. E. & DeSante, D. F. Handbook of field methods for monitoring landbirds. Albany CA US Dep. Agric. For. Serv. Pac. Southwest Res. Stn. Gen Tech Rep PSW-GTR-144 41 P 144, (1993).

Line 467, bat surveys. Can you provide a citation for details on bioacoustics analyses. Are the data used in this manuscript the same data they published elsewhere? That can be fine, but you should be clear and note how the data are used differently here.

We now provide additional information on the bioacoustics analyses and cite the reference keys used for the identification of bat species and sonotypes in the recordings: "*Bat detectors recorded in full spectrum at a sampling rate of 385 kHz. We programmed them to trigger on ultrasonic signals exceeding a minimum threshold of 12 kHz, with a five-second recording duration and a left-channel gain of 12 dB. Recordings were analyzed using Kaleidoscope Pro v.5.6.6 (Wildlife Acoustics, USA) to identify five-second audio files containing at least two bat echolocation calls with frequencies between 8 and 180 kHz, durations of 2–500 ms, and a maximum inter-pulse interval of 500 ms. All the recordings matching these characteristics*

*were classified manually to the species/sonotype level following reference keys for Amazonian bats*[88,89]" (L 574-581).

Additionally, we note that bird and bat data were published in a previous paper and reused here to assess the influence of shade-tree canopy heterogeneity:

"*Bird data was previously published in*[45]*. Here, we expand these analyses to assess the influence of understudied aspects of shade-tree spatial and vertical heterogeneity and identify management practices that potentially promote agroforest biodiversity without yield loss*" (L 563-563).

"*As for birds, the data obtained from the bat surveys were previously published in*[45] *and re-analyzed here to assess the potential of promoting shade-tree spatial and vertical heterogeneity in agroforests as a win-win strategy for biodiversity and farmers*" (L 582-584).

Added references:

88. Lopez-Baucells, A. et al. Field Guide to Amazonian Bats. (2016). doi:10.13140/RG.2.2.23475.84003.

89. Arias-Aguilar, A. et al. Who's calling? Acoustic identification of Brazilian bats. *Mammal Res. 63*, (2018).

My understanding is that acoustic methods for sampling bats can do a good job of capturing the diversity of aerial insectivores but do not do a good job of differentiating between understory bat species. For this, mist nets are preferred. If this is true, then only one group of species was adequately sampled and that the sampling methods may not have adequately captured the bat diversity, particularly the understory species or the species that one might believe to be favored by more forest cover. Please discuss this in your sampling intensity in your response and adjust your manuscript as needed. I refer you to:

Meyer, C.F.J. et al. 2011. Accounting for detectability improves estimates of species richness in tropical bat surveys. Journal of Applied Ecology 48, 777–787.

Meyer, C.F.J. et al. 2010. Long-term monitoring of tropical bats for anthropogenic impact assessment: Gauging the statistical power to detect population change. Biological Conservation 143, 2797–2807.

Indeed, bat species belonging to the family Phyllostomidae cannot be properly sampled using ultrasonic bat recorders. We now acknowledge this limitation and discuss potential implications of landscape forest cover loss for understory bat species:

*"Regarding bats, the sole use of ultrasonic recorders to estimate their species richness prevented us from analyzing shade-tree and landscape management effects on phyllostomid bats (family Phyllostomidae)*[54]*, many of which are understory and forest-dependent species that do not thrive in simplified landscapes*[55]*. Consequently, agroforests must serve as a complement to, rather than a replacement for, remnant forests within tropical agricultural landscapes*[56]*.*" (L 232-236).

"*We surveyed bats using ultrasonic bat detectors (Song Meter Mini Bat, Wildlife Acoustics, USA) positioned approximately 1.5 meters above the ground in 2022 (August to November). While acoustic*

*detectors are the standard methodology for surveying aerial insectivorous bats in the Neotropics[87], they cannot reliably assess phyllostomid species richness due to high structural and frequency overlap in their echolocation calls[54]. Consequently, our bat assessments focus exclusively on the aerial insectivorous guild*" (L 567-571).

Added references:

54. Yoh, N., Syme, P., Rocha, R., Meyer, C. F. J. & López-Baucells, A. Echolocation of Central Amazonian 'whispering' phyllostomid bats: call design and interspecific variation. *Mammal Res*. 65, 583–597 (2020).

55. Klingbeil, B. T. & Willig, M. R. Guild-specific responses of bats to landscape composition and configuration in fragmented Amazonian rainforest. J. Appl. Ecol. 46, 203–213 (2009).

87. Meyer, C. F. J. et al. Accounting for detectability improves estimates of species richness in tropical bat surveys. J. Appl. Ecol. 48, 777–787 (2011).

Line 474, ant surveys. Can you provide a citation to justify sampling efforts and methods and also discuss limitations? My understanding is that the methods are appropriate for sampling arboreal ants but would not account for ground dwelling species. I admittedly do not know the expected percent difference in species but as for bats one might consider the species of ants found on the forest floor of interest in biodiversity surveys. Please discuss in response to reviewers and adjust manuscript as needed.

We now provide a methodological reference (90) and acknowledge that the conclusions of our study regarding shade-tree and landscape effects are limited to the subset of the ant assemblages detected by our sampling methodology: "*Previous comprehensive assessments of ant diversity in primary Amazonian rainforests revealed marked differences in assemblage composition between the forest canopy and ground[91]. Consequently, as we sampled ants only on cacao trees, our conclusions about shade-tree and landscape management effects on ant richness are restricted to this subset of the ant assemblage*" (L 595-598).

Added references:

90. Agosti, D., Majer, J., Alonso, L. & Schultz, T. Ants: Standard Methods for Measuring and Monitoring Biodiversity. Smithsonian Institution Press (Smithsonian Institution Press, Washington, USA, 2000). doi:10.5281/zenodo.11736.

91. Wilkie, K. T. R., Mertl, A. L. & Traniello, J. F. A. Species Diversity and Distribution Patterns of the Ants of Amazonian Ecuador. *PLOS ONE* 5, e13146 (2010).

Line 384, butterfly surveys. It is interesting that you note that you assessed assemblages here. As I am sure you know, species composition is different from species richness, the metric reported in the

manuscript. This overall observation of the biodiversity assessment was made above. With respect to butterflies, please provide a methods citation and discuss how the difference in these metrics affects your conclusions.

We have changed the reference to 'butterfly assemblages' to clarify that we assessed species richness only: "*Butterfly species richness in agroforests was assessed in 2022 (August to November) using Van Someren-Rydon traps baited with bananas fermented for ca. 48 hours prior to deployment*[92]" (L 600-601).

Additionally, we now include the following methodological reference:

92. Freitas, A. et al. Studies with Butterfly Bait Traps: An Overview. *Rev. Colomb. Entomol*. 40, 209–218 (2014).

Line 493, Spider, true bug and beetle surveys. The sampling seems appropriate but, as with other surveys, a methods and sampling citation would be helpful and appreciated.

We have included the following reference:

93. Adis, J., Basset, Y., Floren, A., Hammond, P. & Linsenmair, K. E. Canopy fogging of an overstory tree—recommendations for standardization. *Ecotropica* 4, 93–97 (1998).

Line 511, species richness and multiversity calculation.

Please see overarching comments on biodiversity sampling above. I acknowledge that this was a huge amount of work and is very impressive!

Thank you!

Line 527 exclusion experiments and cacao yield evaluations

This is a huge and impressive amount of work. Very interesting.

Thank you very much for the acknowledgement and kind words!

Line, 566, my understanding is that cacao is pollinated by flies or midges. Is this true? This line suggests more generalist pollinators.

We now provide additional background information on the main suspected cacao pollinators in the revised version: "*Cacao pollination is a complex process involving intricate ecological interactions in which, despite the presumed importance of ceratopogonid midges, the precise identity of the primary pollinators remains a subject of ongoing debate*[100,101]*. Rather than analyzing the cascading effects of arthropod suppression by birds and bats on cacao pollination, we aimed to identify shade-tree and landscape management strategies promoting pollination under natural conditions. Therefore, we assessed pollination services in the agroforests based on the fruit-set of cacao control trees only. As cacao is*

*predominantly self-incompatible, its fruit-set is a good proxy for pollination services in agroforests*[14]" (L 690-696).

100. Van de Sande, E. et al. Revisiting cacao pollination: new behavioural and pollen-based evidence for the key role of Ceratopogonidae. *Basic Appl. Ecol.* 94, 24–33 (2026).

101. Toledo-Hernández, M., Wanger, T. C. & Tscharntke, T. Neglected pollinators: Can enhanced pollination services improve cocoa yields? A review. *Agric. Ecosyst. Environ.* 247, 137–148 (2017).

Line 580, agroforest soil fertility and microclimate

One sample per agroforest at 0-10 cm seems insufficient in terms of soil sampling. Typically, one would take multiple samples per agroforest, bulk them and then subsample if funds were limited (and one could not afford to analyze all samples individually). The sampling needs to be justified and perhaps these data should be excluded from the analysis as any given sample (or multiple single samples) could be idiosyncratic.

Please see our response to the comment on Lines 295 and 296 and our justification for the limited soil sampling above.

Line 611, statistical analyses

The statistical analyses appear to be well done to me but I defer to other reviewers with stronger analytical abilities. My concerns (which may be too strong of a word) are rather with the data that go into the analyses as noted above.

Thank you very much for your comments and thorough review. We hope we have addressed these concerns successfully.
